# Contributions to the *Inocybe umbratica–paludinella* (*Agaricales*) Group in China: Taxonomy, Species Diversity, and Molecular Phylogeny

**DOI:** 10.3390/jof10120893

**Published:** 2024-12-23

**Authors:** Xin Chen, Wen-Jie Yu, Tolgor Bau, P. Brandon Matheny, Egon Horak, Yu Liu, Li-Wu Qin, Li-Ping Tang, Yu-Peng Ge, Tie-Zhi Liu, Yu-Guang Fan

**Affiliations:** 1 Engineering Research Center of Tropical Medicine Innovation and Transformation of Ministry of Education, International Joint Research Center of Human-Machine Intelligent Collaborative for Tumor Precision Diagnosis and Treatment of Hainan Province, Hainan Provincial Key Laboratory of Research and Development on Tropical Herbs, School of Pharmacy, Hainan Medical University, Haikou 571199, China; chenxin@hainmc.edu.cn (X.C.); hy0204046@hainmc.edu.cn (W.-J.Y.); 2 Engineering Research Centre of Chinese Ministry of Education for Edible and Medicinal Fungi, Jilin Agricultural University, Changchun 130118, China; junwusuo@126.com; 3 Department of Ecology and Evolutionary Biology, University of Tennessee, Knoxville, TN 37996, USA; pmatheny@utk.edu; 4 Independent Researcher, Schlossfeld 17, AT-6020 Innsbruck, Austria; sporax@gmx.net; 5 Shandong Key Laboratory of Edible Mushroom Technology, School of Agriculture, Ludong University, Yantai 264025, China; liuyu8006@163.com (Y.L.); gaiyupeng@126.com (Y.-P.G.); 6 Jilin Provincial Joint Key Laboratory of Changbai Mountain Biocoenosis and Biodiversity, Changbai Mountain Academy of Sciences, Antu 133613, China; qinliwu2003@163.com; 7 School of Pharmaceutical Sciences and Yunnan Key Laboratory of Pharmacology for Natural Products, Kunming Medical University, Kunming 650500, China; tangliping@kmmu.edu.cn; 8 College of Chemistry and Life Sciences, Chifeng University, Chifeng 024000, China; tiezhiliu@aliyun.com

**Keywords:** Asia, systematics, *Inocybaceae*, new taxa, *rpb2*

## Abstract

*Inocybe* is the largest genus in the family *Inocybaceae*, with approximately 1000 species worldwide. Basic data on the species diversity, geographic distribution, and the infrageneric framework of *Inocybe* are still incomplete because of the intricate nature of this genus, which includes numerous unrecognized taxa that exist around the world. A multigene phylogeny of the *I. umbratica–paludinella* group, initially designated as the “*I. angustifolia* subgroup”, was conducted using the ITS-28S-*rpb2* nucleotide datasets. The seven species, *I. alabamensis*, *I. angustifolia*, *I. argenteolutea*, *I. olivaceonigra*, *I. paludinella*, *I. subangustifolia*, and *I. umbratica*, were confirmed as members of this species group. At the genus level, the *I. umbratica–paludinella* group is a sister to the lineage of the unifying *I. castanea* and an undescribed species. *Inocybe* sect. *Umbraticae* sect. nov. was proposed to accommodate species in the *I. umbratica–paludinella* group and the *I. castanea* lineage. This section now comprises eight documented species and nine new species from China, as described in this paper. Additionally, new geographical distributions of *I. angustifolia* and *I. castanea* in China are reported. The nine new species and *I. angustifolia*, *I. castanea*, *I. olivaceonigra*, and *I. umbratica* are described in detail and illustrated herein with color plates based on Chinese materials. A global key to 17 species in the section *Umbraticae* is provided. The results of the current study provide a more detailed basis for the accurate identification of species in the *I. umbratica-paludinella* group and a better understanding of their phylogenetic placement.

## 1. Introduction

*Inocybaceae* is a family of the mushroom *Agaricales*, with seven genera: *Inocybe sensu stricto* (s.s.), *Auritella* Matheny & Bougher, *Inosperma* (Kühner) Matheny & Esteve-Rav, *Mallocybe* (Kuyper) Matheny, Vizzini & Esteve-Rav, *Nothocybe* Matheny & K.P.D. Latha, *Pseudosperma* Matheny & Esteve-Rav, and *Tubariomyces* Esteve-Rav. & Matheny [1]. The fungi in this family are important because they form mycorrhizal symbiosis with a wide range of plants [1,2], which play a vital role in forest ecosystems. Many species in this family contain neuropsychotoxins [3,4,5,6,7,8], which usually lead to poisoning incidents [9,10,11,12,13,14,15]. Accordingly, the basic data on species diversity, geographic distribution, and their toxins are crucial to the poisoning prevention and utilization of this group of fungi. *Inocybe* is the largest genus, with an estimated 1000 species worldwide [16]. This genus is characterized by the presence of cheilocystidia and generally also pleurocystidia with thick walls, whereas this is not the case with the other six genera [1,17]. The existing infrageneric framework of this genus is mainly based on morphological data [18,19] and is not supported by molecular phylogenetic studies [1]. A genus-level phylogeny can hardly be achieved at present because of the huge number of documented species, and a large number of species are still being discovered from all over the world [20,21,22,23,24,25,26,27,28,29,30,31,32,33,34,35,36].

*Inocybe paludinella* (Peck) Sacc. was originally described as *Agaricus paludinellus* Peck from Sand Lake, New York in 1879 and subsequently combined into the genus *Inocybe* by Saccardo in 1887. This species has been in lowland and wet habitats (original description), as well as under frondose and coniferous forests [37,38,39]. It can be confused with white and some grayish species *I. umbratica* Quél., which were originally described from France in 1884 and widely reported in northern temperate regions, including Europe [37,40,41], East Asia [19], and North America [42]. The latter species occurs in coniferous forests under *Picea*, *Abies*, or *Larix*. These two species share a slender habit, silver–white to yellowish basidiomata, a thin pileus with narrow lamellae, angular–nodulose basidiospores, and abundant thick-walled hymenial cystidia, as well as an overlapped ecology. Stangl (1975) proposed that these two species should be united [43], a view that was accepted by Breitenbach and Kranzlin (2000) [39], but most European mycologists treated them as separate species, with the distinction of yellow-ocherish basidiomata of *I. paludinella* even when young [37,38,44]. *Inocybe paludinella* was more often placed in *I.* sect. *Marginatae* [18,45], while Bon (1998) assigned *I. paludinella* and *I. umbratica* to *I.* sect. *Petiginosae*. However, a series of molecular phylogenetic studies demonstrated that *I.* sect. *Marginatae* typified by *I. asterospora* Quél. was polyphyletic [46,47,48,49].

In North America, *Inocybe alabamensis* Kauffman is another species with a thin light silver–gray pileus, yellowish stipes, and abundant cystidia [50]. It was described from Alabama (Southeastern USA) in the 1920s. Matheny and Bougher (2017) considered this species as a taxonomic synonym of *I. paludinella*, but critical comparisons between the two species have yet to be made [51]. Furthermore, *Inocybe suaveolens* D.E. Stuntz, described from Washington (USA), and *I. alachuana* Murrill, described from Florida, were considered as synonyms of *I. umbratica*. In addition, *Inocybe angustifolia* Horak, described from Papua New Guinea, and *I. senkawaensis* Kobayasi, described from Japan, share similarities with *I. umbratica* to some extent [19,52].

In the molecular era, a three-gene phylogenetic study of *Inocybaceae* on a global scale demonstrated that *I. alabamensis* is clustered with *I. paludinella* and two tropical Asian materials in a well-supported clade. This clade is closely related to *I. torresiae* Matheny, Bougher & Barrett [47]. Subsequently, in a multigene phylogeny, *I. umbratica* and *I. argenteolutea* Vauras formed a fully supported lineage with no clear relationship with respect to other lineages of this genus [48]. Fan and Bau (2014) reported the olive-green species *I. olivaceonigra* in subtropical China [53]. This species showed close affinities with *I. umbratica* or *I. suaveolens*, as inferred by ITS BLAST results. One year later, an LSU phylogeny revealed that *I. angustifolia*, *I. umbratica*, *I. alabamensis*, and an undescribed taxon from Thailand grouped into a significant support clade [54]. Matheny and Bougher (2017) indicated that *I. subangustifolia* Matheny, Bougher & Halling from Australia, is phylogenetically close to several northern temperate species, including *I. alabamensis*, *I. argenteolutea*, *I. paludinella*, and *I. umbratica* [51].

Prior to our study, eleven of the aforementioned species were considered to be related to the *I. umbratica–paludinella* group. However, the systematic position and phylogenetic inference of this species group remained unclear. No prior work has focused on the species diversity, taxonomy, and phylogeny of the *I. umbratica–paludinella* group, especially in China. The present study proposes the recognition of nine new species and new geographical distributions for two previously recorded species within the *I. umbratica–paludinella* group, based on our collections from temperate and subtropical China. For a more comprehensive understanding of the phylogenetic inferences of this group of fungi, a summarized phylogenetic analysis of *Inocybe* as a genus was also performed.

## 2. Materials and Methods

### 2.1. Research Area and Specimen Sampling

Fresh material was collected during field expeditions in Yunnan, Jilin, Guangdong, Sichuan, Zhejiang, and Shandong Provinces in China between 2013 and 2024. The samples were dried overnight at 45 °C using an electronic dryer and then preserved in plastic bags and sealed. After the study, the specimens were deposited at the Herbarium of Changbai Mountain National Natural Reserve (ANTU) with FCAS numbers, the Mycological Herbarium of Chifeng University (CFSZ), the Mycological Herbarium of Kunming Medical University (MHKMU), the Herbarium of Mycology of Jilin Agricultural University (HMJAU), and the Mycological Herbarium of Ludong University (HMLD).

### 2.2. Morphological Study

Macroscopic characteristic descriptions were derived from field notes and color photographs. The size of each part of the basidiomata was recorded according to actual measurements in the field. Color identification was based on Kornerup and Wanscher [55]. Mushroom tissues from the pileus, lamellae, and stipe were cut into thin sections freehand with a blade under a stereoscope. Microscopic features were observed using a Olympus CX23 light microscope (Olympus Co., Ltd., Tokyo, Japan). They were examined in 5% KOH, and the sample was stained, if necessary, with a 1% aqueous solution of Congo red, at 100×, 400×, and 1000× magnifications. The sizes of all the measured elements are given as length × width. In the following descriptions, the ratio of the length to the width of the basidiospores is represented by Q, Q_m_ is the mean value, and SD is the sample’s standard deviation. The expression (n/m/p) means that n basidiospores were measured from m basidiomata of p samples. Data for basidiospores are represented as (a) b–c–d (e), where a and e are the extreme values, b and d are the values at both ends of the 90% confidence interval, and c is the average value.

### 2.3. DNA Extraction, PCR, and Sequencing

Genomic DNA was extracted from dried specimens using the NuClean plant genomic DNA kit (Cwbio, Taizhou, China). The following primers were used. Primers for ITS included ITS1-F/ITS4 for internal transcribed spacer (ITS) [56]. Primers for 28S included LR0R/LR7 for the nuclear large subunit ribosomal (LSU) [57]. Primers for *rpb2* included rpb6F and rpb7.1R for the second largest subunit of RNA polymerase II (*rpb2*) [46]. The volume of the polymerase chain reaction (PCR) mixture solution was 25 μL, containing 9.5 μL of dd H_2_O, 12.5 μL of 2× Taq Plus MasterMix with blue dye (Cwbio, Taizhou, China), 1 μL of each primer, and 1 μL of the template DNA. The PCR conditions for ITS, LSU, and *rpb2* were as follows: the PCR conditions for the three different gene regions were all the same as those of the initial denaturation at 95 °C for 1 min at first, followed by 35 cycles of denaturation at 95 °C for 30 s, annealing at 52 °C for 1 min, extension at 72 °C for 1 min, and a final extension at 72 °C for 8 min. The amplification products were then sent to Sangon Biotech (Shanghai, China) for purification and sequencing as soon as possible. BioEdit version 7.0.9.0 was used to check and verify the quality of the sequences [58].

### 2.4. Taxon Sampling, Sequence Alignment, and Phylogenetic Analyses

New sequences derived in this study were compared with closely related *Inocybe* sequences obtained from GenBank using the BLASTn search tool (https://blast.ncbi.nlm.nih.gov/Blast.cgi, accessed on 1 September 2024), and the BLAST results of close matches from GenBank were selected for phylogenetic analyses. In addition, sequences of major sections, clades, or groups in the genus *Inocybe* were selected and combined in the datasets for a genus-level phylogeny [59,60]. *Nothocybe distincta* K.P.D. Latha & Manim (CAL1310 and ZT9250) were used as outgroups [1,47]. The dataset for each locus was aligned using the MAFFT v. 7 online service (https://mafft.cbrc.jp/alignment/server/, accessed on 14 September 2024) [61] and manually adjusted using BioEdit version 7.0.9.0 [58]. The three datasets were concatenated using MEGA 5.0 [62]. Maximum likelihood (ML) analyses were performed in the W-IQ-TREE web service (http://iqtree.cibiv.univie.ac.at/, accessed on 14 September 2024) with 1000 replicates [63]. The results were processed using FigTree v. 1.4.3 software. The ML bootstrap proportions (ML-BP) ≥ 70% were considered as evidence of strong support for any given internode. The phylogenetic tree was refined using the online tool TVBOT 2.3.6 (https://www.chiplot.online/tvbot.html, accessed on 26 September 2024).

## 3. Results

As shown in Table 1, sequences from 295 taxa (269 ITS, 209 LSU, and 111 *rpb2*), including 162 new sequences (81 ITS, 47 LSU, and 34 *rpb2*) that were submitted to GenBank, were analyzed in this study. The final alignment has 295 taxa with 2865 columns, 1028 parsimony-informative, 293 singleton sites, 1544 constant sites (975 ITS, 1331 LSU, and 559 *rpb2*), including gaps, and has been deposited in TreeBase (ID31709). A final ML optimization likelihood value of −45,463,800 was achieved. There were 1669 unique patterns in the combined alignment. The ML analysis of the combined dataset resulted in a single best ML tree of −LnL = 45733.677. The following models, selected by BIC, were used for the ML analysis: TVM + F + I + G4 for ITS, GTR + F + I + G4 for LSU, and TIM3e + I + G4 for *rpb2*.

**Figure 1:**
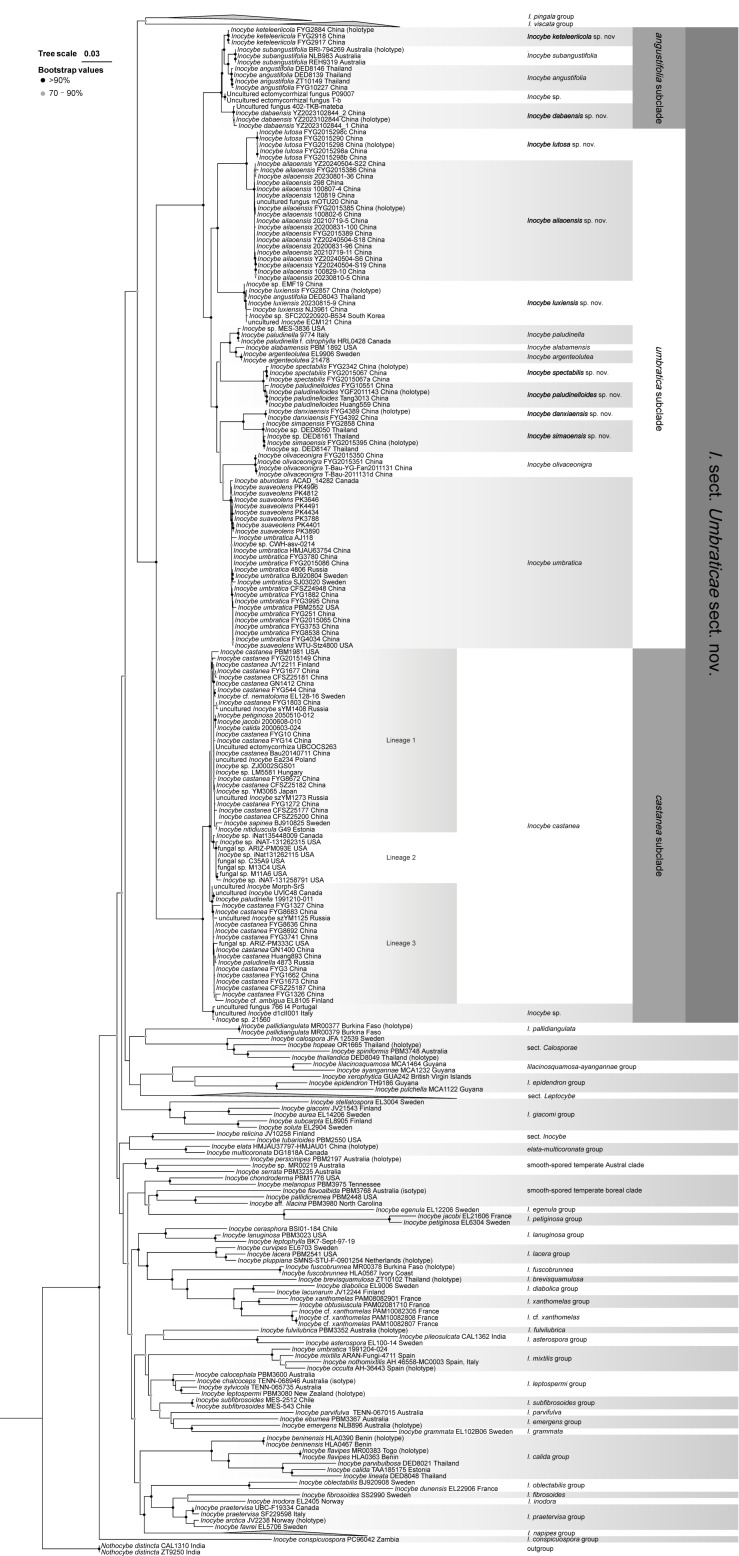
Maximum likelihood phylogenetic tree of a combined dataset of *Inocybe* nuclear gene sequences (ITS, LSU, and *rpb2*), with *Nothocybe distincta* (ZT9250 and CAL1310) used as outgroups. ML bootstrap percentages (ML-BP) ≥ 70 are shown at each internode. New species are in bold.

In the three-gene phylogeny (Figure 1), the genus *Inocybe* was fully supported with numerous clades, which relationships are not clearly resolved. The *I. umbratica–paludinella* group was fully supported and was a sister to the *I. castanea* lineage; these taxa clustered with the lineage unifying the *I. pingala* group and the *I. viscata* group in a well-supported clade (BP = 76%). The nine newly identified species received high support and were placed in the *I. umbratica–paludinella* group. Within the *I. umbratica–paludinella* group, two major subgroups were recovered, namely, the *angustifolia* subgroup (BP = 100%) and the *umbractica* subgroup (BP = 99%). The *angustifolia* subgroup comprised two documented species, *I. angustifolia* and *I. subangustifolia*, which were in a sister relationship with each other, with this lineage, in turn, being a sister to the new species *I. keteleeriicola*; another new species, *I. dabaensis*, is a sister to the remainder in this subgroup. The *umbratica* subgroup included twelve species, seven of which were newly identified species in this study. *Inocybe olivaceonigra* is a sister to *I. umbratica*, these two species, in turn, are sisters to the lineage united by *I. simaoensis* and *I. danxiaensis*. Unexpectedly, the white species, *I. simaoensis*, is a sister to the yellow species, *I. danxiaensis. Inocybe paludinelloides* and *I. spectabilis,* both of which share yellow basidiomata, are sisters to each other. *Inocybe lutosa* and *I. ailaoensis* are in a sister relationship with each other, with this lineage, in turn, being a sister to *I. luxiensis*; these three species have an earthy yellow-tinged pileus. The *I. castanea* subclade is composed of the north temperate species *I. castanea* Peck and an undescribed phylogenetic taxon. Three well-supported phylogenetic lineages were identified in *I. castanea* and designated as Lineage 1 to Lineage 3.

## 4. Taxonomy

***Inocybe* sect. *Umbraticae*** Y.G. Fan, Matheny & T. Bau, sect. nov.

**Chinese name**. 丝盖伞属荫生组

MycoBank. MB855894.

**Etymology**. Umbraticus (Latin), means in the shadow, in reference to the habitat where most species occur.

**Type**. *Inocybe angustifolia* (Corner & Horak, E.) *Garrido*, *Biblthca Mycol*. 1988, *120*, 176.

**Diagnosis**. Basidiomata small, slender, white, grayish-white, yellowish, or reddish-brown; Pileus hemispherical, campanulate, convex to applanate, without veil remnants, surface glabrous, silky smooth, appressed-fibrillose to fibrillose-rimulose; lamellae mostly crowded to rather crowded, occasionally moderately crowded, edge finely serrate but non-fimbriate; stipe cylindrical, solid, central with a swollen but non-bulbose base, entirely pruinose or pruinose-furfuraceous. Basidiospores angular–nodulose with small rounded nodules and usually an elongate knob at the apex; hymenial cystidia abundant, fusiform to broadly fusiform with noticeably thickened yellowish walls, cheiloparacystidia abundant, clavate to obovoid; caulocystidia descending to stipe base. Pileipellis a cutis of two layers, the upper layer colorless, more or less gelatinized in some species, hyphae cylindrical, smooth, lower layer yellowish, hyphae inflated, finely encrusted. Odor spermatic, aromatic, sweet, fungoid, or acidulous. Occurs in North temperate, subtropical to tropical Asia, tropical America, and Australia. Forming plant associations with *Betulaceae*, *Fagales*, *Myrtaceae*, and *Pinaceae*.

**Included species**—*I. alabamensis*, *I. angustifolia*, *I. argenteolutea*, *I. castanea*, *I. dabaensis* sp. nov., *I. danxiaensis* sp. nov., *I. keteleeriicola* sp. nov., *I. lutosa* sp. nov., *I. luxiensis* sp. nov., *I. paludinella*, *I. paludinelloides* sp. nov., *I. simaoensis* sp. nov., *I. spectabilis* sp. nov., *I. subangustifolia*, *I. umbratica*.

**Species of uncertain position**—*I. senkawaensis*, *I. straminea*.

**Notes:** Species in *I.* sect. *Umbraticae* were separated from *I.* sect. *Marginate*, which is not monophyletic. Species in *I.* sect. *Albodiscae* Matheny, recently cleaved from *I.* sect. *Marginatae* differs by larger and more robust basidiomata and bi-colored pileus [101]. Members in *I.* sect. *Petiginosae* Heim has conspicuously smaller basidiomata, usually pinkish stipe, and thick-walled epicutis hyphae in pileus; species of *I.* sect. *Calosporae* J.E. Lange have spinose basidiospores; species of *I*. sect. *Rubellae* Kühner & Bousier have reddened flesh [18]. Among the species groups assigned in *I*. sect. *Marginatae*, the type species *I. asterospora* and most taxa in this section have stellate basidiospores. Species in the *I. praetervisa* group, also known as *I*. subsect. *Praetervisae* Bon differs by larger basidiomata, more prominent nodules in basidiospores, and abundant caulocystidia in upper part but sparse or absent in lower part of stipe [88]. Species of the *I*. *xanthomelas* group or *xanthomelas* clade differ either by their darkening stipe and/or elongate or slender and nearly lageniform cystidia characterized by a protruding neck [68,88]. Species in the *I. mixitilis* group differs by more robust stipes with more or less bulbose marginate bases, yellow to brown pileus, and more pronounced nodules in basidiospores [83]. *Inocybe umbratica* f. *aurantiaca* Takah. Kobay. is not included in sect. *Umbraticae* because of its close relationship with *I. caroticolor* T. Bau & Y.G. Fan [53].

**Figure 2:**
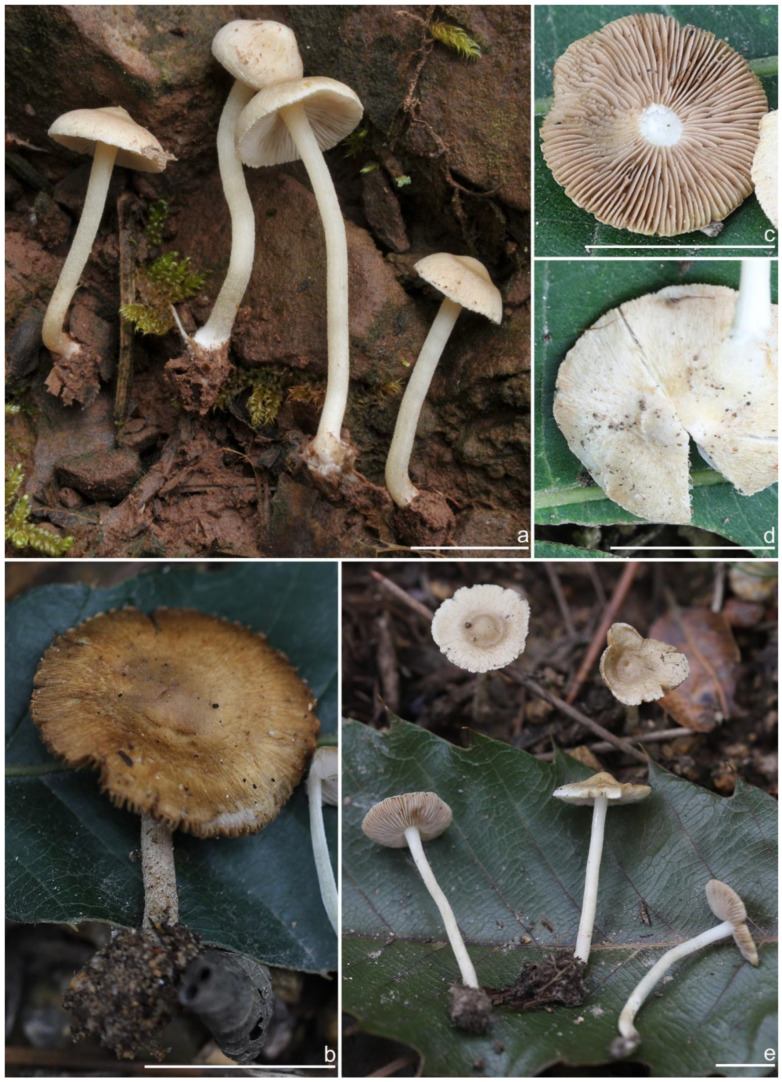
Basidiomata of *Inocybe ailaoensis*. (**a**) FYG2015385 (FCAS3848, holotype); (**b**) HMLD4191; (**c**–**e**) HMLD4188; Scale bars: 10 mm. (**a**) Photo by Y.-G. Fan. (**b**–**e**) Photos by Y. Liu.

**Figure 3:**
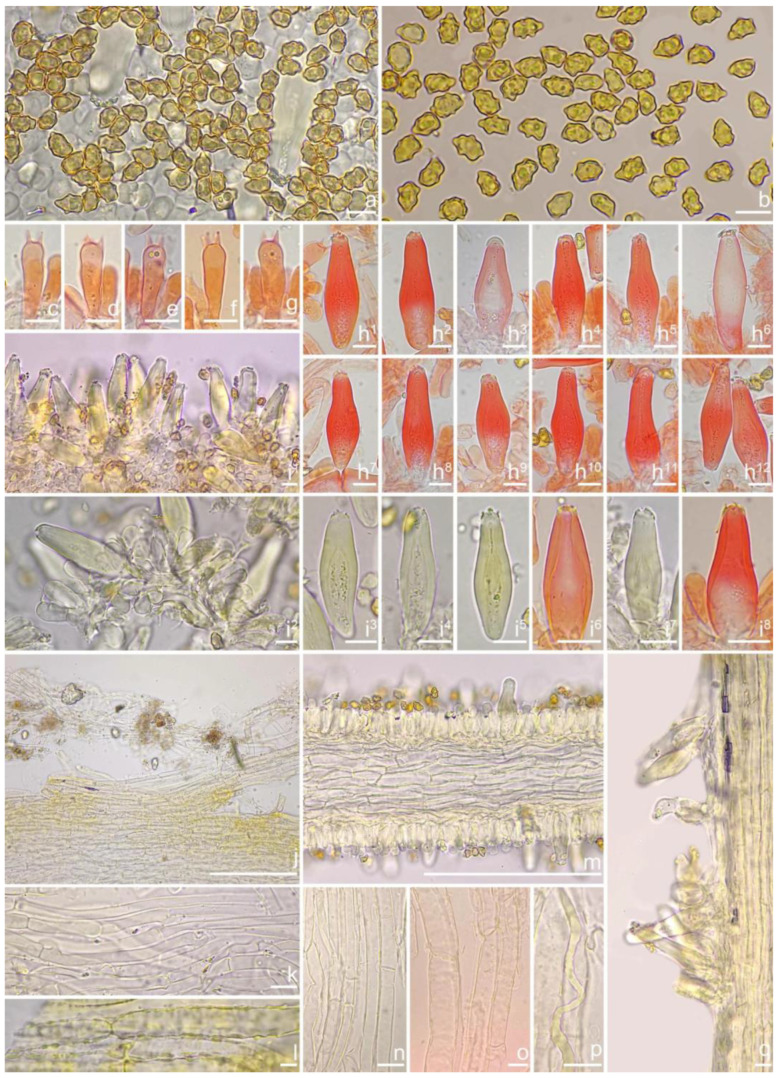
Microscopic features of *Inocybe ailaoensis* (FCAS3848, holotype). (**a**,**b**) Basidiospores. (**c**–**g**) Basidia. (**h^1^**–**h^12^**) Pleurocystidia. (**i^1^**–**i^8^**) Cheilocystidia. (**j**) Pileipellis. (**k**) Pileipellis upper layer hyphae. (**l**) Pileipellis lower layer hyphae. (**m**) Cross-section of lamellae. (**n**) Stipitipellis hyphae. (**o**) Stipe trama hyphae. (**p**) Oleiferous hyphae. (**q**) Caulocystidia. Scale bars: (**a**–**i^8^**,**k**,**l**,**n**–**q**) 10 μm, (**j**,**m**) 100 μm. Photos by X. Chen.

***Inocybe ailaoensis*** Y.G. Fan, X. Chen & W.J. Yu, sp. nov. Figure 2 and Figure 3

**Chinese name**. 哀牢山丝盖伞

**MycoBank**. MB 855820

**Etymology**. In reference to Ailao Mountain (Yunnan Province, China), where the type specimen was collected.

**Diagnosis**. *Inocybe ailaoensis* has small, slender basidiomata, ivory white to grayish-white, obtusely umbonate pileus, fusiform hymenial cystidia with noticeably thickened walls. Most similar to *I. senkawaensis* but differs in higher Q-values of basidiospores and fusiform hymenial cystidia. Phylogenetically, it is a sister to *I. lutosa* but differs in pileus color, more prominent nodulose basidiospores, obtuse, non-pedicellate hymenial cystidia, and a 5.17% difference in ITS sequence.

**Holotype**. China, Yunnan Province, Pu’er City, Jingdong Yi Autonomous County, Ailao Mountain, occurring in fagaceous forests, 24°32′36″ N, 101°1′29″ E, 22 September 2015, Y.G. Fan, FYG2015385 (FCAS3848), GenBank accession number: ITS (PP217739); LSU (PP230780) and RPB2 (PP238456).

*Basidiomata* small-sized. *Pileus* 6–23 mm in diameter, hemispherical when young, campanulate-convex to plano-convex with a broad low umbo when mature, margin crenulate, depressed when young, later straight, uplifted when mature, longer than lamellae; surface dry, glabrous toward the center, finely subtometose outward when young, silky smooth to radically fibrillose upon maturity; uniformly cream white (2A1), ivory white (23A1) to buff white (1A2). *Lamellae* 0.5–2.0 mm deep, adnexed, crowded, often with unequal lamella, edges paler, crenulate to slightly serrate when mature; cream white (1A1) when young, then pale pinkish (13A1) to fawn (2A2) upon maturity. *Stipe* 16–42 × 1–3 mm, central, solid, terete, equal with a slightly swollen base, densely pruinose along the entire length; grayish-white (1A1) to beige (1B1). *Context* solid, fleshy in pileus, 0.5–0.8 mm thick at mid-radius, up to 1.5 mm thick under the umbo, fibrillose and striate in the stipe, cream white (2A1); base often fleshy, beige (1B1) or white (1A1). *Odor* spermatic or somewhat aromatic.

*Basidiospores* [*n* = 100/2/2] (6.0) 6.1–7.1–8.1 (8.9) × (4.1) 4.2–5.1–5.8 (6.2) μm, Q = (1.13) 1.20–1.42–1.64 (1.77), Q_m_ ± SD = 1.42 ± 0.126, angular–nodulose with 7–15 small nodules, pale yellow to goldish-yellow, with an inconspicuous subconical knob at apex, apiculus indistinct, with intracellular circular to amorphous oil inclusions. *Basidia* 18–26 × 6–8 μm, mostly claviform to narrowly claviform, hyaline, thin-walled, mostly 4-sterigmate, but 2-sterigmate basidia occasionally observed, sterigmata 2–4 μm in length. *Pleurocystidia* 33–**45**–53 × 12–**14**–17 μm (*n* = 40), abundant, mostly fusiform and broadly fusiform, occasionally lageniform, crystallization present at the obtuse apices, base nearly rounded to obtuse, thick-walled, walls up to 3 μm wide at the middle and strikingly thickened near the apex (up to 5 μm), pale yellowish. *Cheilocystidia* 28–34–47 × 10–14–16 μm (*n* = 40), abundant, resemble pleurocystidia, mostly fusiform to broadly fusiform, at times sublageniform. *Cheiloparacystidia* 8–17 × 4–7 μm, clavate to oblong, hyaline, colorless, thin-walled, mixed with cheilocystidia. *Caulocystidia* 34–45–56 × 10–15–19 μm (*n* = 30), descending to stipe base, fusiform, lageniform, or broadly lageniform, thick-walled, crystallization at apices. *Cauloparacystidia* 10–16 × 6–10 μm, abundant, pyriform, ovoid, clavate, or oblong, thin-walled, colorless, mixed with caulocystidia. *Hymenophoral trama* 40–70 μm thick, regularly arranged, hyphae thin-walled, colorless, smooth, slightly inflated, 3–15 μm wide, cylindrical hyphae occasionally observed. *Pileipellis* divided into two layers, upper layer interwoven, colorless, 65–125 μm thick, hyphae cylindrical, smooth, thin-walled, 2–6 μm wide; lower layer regularly arranged, hyphae slightly inflated, 5–13 μm wide, yellowish to yellowish-brown, indistinctly encrusted or rough. *Pileal trama* 350–500 μm thick, subregularly arranged, colorless, hyphae 5–16 μm wide, smooth, thin-walled, inflated. *Stipitipellis* regularly arranged, pale yellowish in color, composed of cylindrical hyphae, 5–16 μm wide, thin-walled, smooth. *Oleiferous hyphae* 1–4 µm wide, yellowish, diverticulate, mostly present in the pileal and the stipe trama. *Clamp connections* present and common in all tissues.

**Habitat**. Single or in small groups under fagaceous trees.

**Distribution**. China (Yunnan, Shandong, Sichuan).

**Additional specimens examined**. China, Yunnan Province: Pu’er City, Jingdong Yi Autonomous County, Ailao Mountain, in *Fagaceae* forests, 24°32′36″ N, 101°1′29″ E, 22 September 2015, Y.G. Fan, FYG2015386 (FCAS3849), FYG2015389 (FCAS3607); Shandong Province: Yantai City, Haiyang City, Xujiadian, Laozhai Mountain, in broad-leaved forests, 1 August 2023, Y. Liu, 20230801-36 (FCAS4062), Yunding Scenic Spot, 26 July 2009, T. Bau, HMLD298; Zhifu District, Jinkuang Mountain, in *Fagaceae* forests, 10 August 2023, Y. Liu, 20230810-5 (FCAS4063); Nanshan Park, in broad-leaved forests, 2 August 2010, Y. Liu & J.R. Wang, 100802-6 (HMLD1233); Ta Mountain, in mixed forest, 7 August 2010, Y. Liu & J.R. Wang, 100807-4 (HMLD1321); Ludong University, Ruzi Mountain, in mixed forests, 29 August 2010, Y. Liu, 100829-10 (HMLD1345); Linyi City, Mengyin County, Meng Mountain, 19 August 2010, J.R. Wang, 120,819 (HMLD1988); Zhaoyuan City, Luo Mountain, Xiaoyuanmiao Forest Farm, roadside near mixed forests, 7 September 2020, Y. Liu, 20200831-96 (HMLD3572), in broad-leaved forests, 19 July 2021, Y. Liu, 20210719-5 (HMLD4188), 20210719-11 (HMLD4191); Great Falls Scenic Area, in mixed forest, 22 July 2020, Y. Liu, 20200831-100 (HMLD3576); Sichuan Province: Dazhou City, Xuanhan County, Wanqiao Village, in mixed-forests, 31°23′44″ N, 107°34′37″ E, 4 May 2024, X.M. Yang, YZ20240504-S6 (FCAS4016), YZ20240504-S18 (FCAS4017), YZ20240504-S19 (FCAS4018), YZ20240504-S22 (FCAS4019).

**Remarks**. *Inocybe ailaoensis* was found in four provinces in warm temperate to subtropical China. It is characterized by the small white basidiomata, subfusiform hymenial cystidia with strikingly thickened walls. *Inocybe senkawaensis* Kobayasi, described from Japan is superficially similar to *I. ailaoensis*, but the former has less pronounced nodules, lower Q values of basidiospores, and mostly cylindrical to subcylindrical hymenial cystidia [19,102]. *Inocybe ailaoensis* is phylogenetically a sister to *I. lutosa*, but the latter species has earthy yellow pileus, less tapered ends at the apices of basidiospores, fusiform to subfusiform hymenial cystidia usually with tapered bases. The comparison of the sequences between the two species reveals 4.89% differences (31/634) in ITS, including twelve base pairs and six insertion–deletion events (2–10 bp in length), 0.15% differences (2/1301) in LSU, and 1.32% differences (8/602) in *rpb2*.

***Inocybe angustifolia*** (Corner & Horak, E.) Garrido, Biblthca Mycol. 1988, 120, 176

Figure 4

*Astrosporina angustifolia* Corner & Horak, E. Persoonia 1979, *10*, 195.

**Chinese name**. 密褶丝盖伞.

*Basidiospores* [*n* = 100/1/1] (7.1) 7.2–8.0–9.0 (10.1) × (4.8) 4.9–5.6–6.1 (6.5) μm, Q = (1.18) 1.24–1.44–1.67 (1.83), Q_m_ ± SD = 1.44 ± 0.13, with 8–16 small nodules on an angular outline, yellowish or greenish-yellow under 5% KOH, apiculus indistinct, at times with intracellular circular to oblong oil inclusions. *Basidia* 18–25 × 7–10 μm, mostly claviform, broadly claviform, hyaline, mostly 4-spored, but 2-spored basidia occasionally observed, sterigmata 2–5 μm in length. *Pleurocystidia* 44–52–63 × 15–20–20 μm (*n* = 40), mostly fusiform to broadly fusiform, at times lageniform, crystallization present at the obtuse or rounded apices, base tapered but never form a distinct pedicel, thick-walled, walls up to 4 μm thick toward the apices, thinner downward, pale yellowish or greenish, extremely abundant. *Cheilocystidia* 41–47–64 × 12–14–17 μm (*n* = 40), resemble pleurocystidia, mostly fusiform or broadly fusiform, occasionally lageniform, walls strikingly thickened near the apices (up to 4 μm), thinner downward, pale greenish. *Cheiloparacystidia* 10–17 × 6–9 μm, clavate to ovoid, hyaline, thin-walled, smooth, mixed with numerous metuloidal cheilocystidia. *Caulocystidia* resemble pleurocystidia, descending to stipe base. *Hymenophoral trama* 90–175 μm thick, subregularly arranged, colorless, composed of thin-walled, smooth, mostly inflated or cylindrical hyphae, 4–23 μm wide. *Pileipellis* divided into two layers, 90–180 μm wide, upper layer interwoven, colorless, hyphae cylindrical, smooth, thin-walled, hyaline 3–5 μm wide; lower layer regularly arranged, subinflated hyphae, yellow to yellowish-brown, encrusted, hyphae 9–22 μm wide. *Pileal trama* regularly arranged, composed of inflated and cylindrical hyphae up to 25 μm in diameter, colorless, smooth, thin-walled. *Oleiferous hyphae* 3–6 µm wide, pale yellowish, smooth, mostly present in pileal trama. *Clamp connections* present and common in all tissues.

**Habitat**. On humid soil, under fagaceous trees.

**Distribution**. Papua New Guinea (type), Indonesia, Malaysia, northwestern Thailand, China (Yunnan).

**Specimens examined**. China, Yunnan Province, Pu’er City, Jingdong Yi Autonomous County, Ailao Mountain, in *Fagaceae* forests, 22°49′47″ N, 100°58′37″ E, 23 September 2015, Y.G. Fan, FYG10227 (FCAS4010), GenBank accession numbers: ITS (PP217734); LSU (PP230773) and RPB2 (PP238451).

**Remarks**. *Inocybe angustifolia* is considered as a widespread species in Indomalaya and Southeast Asia [103], where it occurs in association with *Dipterocarpus* or *Castanopsis*, *Lithocarpus* and *Quercus* with scattered *Pinus* in the tropical montane rainforest [52,54]. The most distinctive feature of *I. angustifolia* are a pileus (and stipe) at first whitish, becoming pale yellow or fawn with age, narrow, densely crowded lamellae, and an entirely pruinose stipe that is cylindrical-equal or with a swollen to submarginate base. The basidiospores are relatively small, with numerous subconical or hemispherical nodules. Cheilocystidia and pleurocystidia often have yellowish parietal pigment (in 5% KOH).

Currently available sequence data for *I. angustifolia* in GenBank were generated from Thai materials by Horak et al. (2015), but no material from Papua New Guinea (type locality) has been sequenced [54]. We obtained one specimen of *I. angustifolia* from central Yunnan but with insufficient macroscopic data. The identification of this specimen was mainly based on molecular phylogeny and micro-examinations. Microscopically, The Chinese materials agree well with the original description and the redescription of this species [52,54]. Phylogenetically, *I. angustifolia* is a sister to *I. subangustifolia*, a dull yellow to greenish-yellow species described from Australia which occurs under *Eucalyptus* and *Acacia* [51]. *Inocybe keteleeriicola* and *I. dabaensis* are phylogenetically close to *I. angustifolia*, but *I. keteleeriicola* differs in having less crowded lamellae, smaller basidiospores, and occurring in *Keteleeria* forests; *I. dabaensis* differs from *I. angustifolia* by the longer basidiospores with more prominent nodules and thinner walled hymenial cystidia with a nearly thin-walled base.

**Figure 6:**
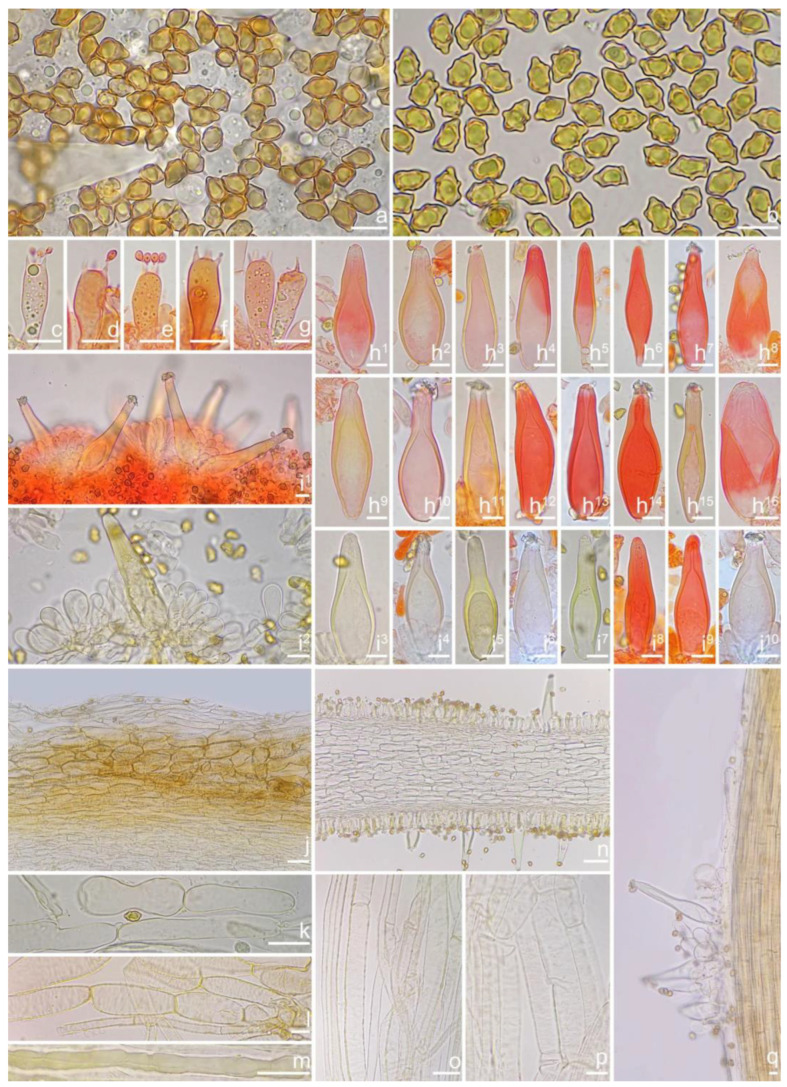
Microscopic features of *Inocybe castanea* (FCAS3865). (**a**,**b**) Basidiospores. (**c**–**g**) Basidia. (**h^1^**–**h^16^**) Pleurocystidia. (**i^1^**–**i^10^**) Cheilocystidia. (**j**) Pileipellis. (**k**) Pileipellis upper layer hyphae. (**l**) Pileipellis lower layer hyphae. (**m**) Oleiferous hyphae. (**n**) Cross-section of lamellae. (**o**) Stipitipellis hyphae. (**p**) Stipe trama hyphae. (**q**) Stipitipellis with caulocystidia and cauloparacystidia. Scale bars: (**a**–**m**,**o**–**q**) 10 μm; (**n**) 100 μm. Photos by X. Chen.

**Figure 7:**
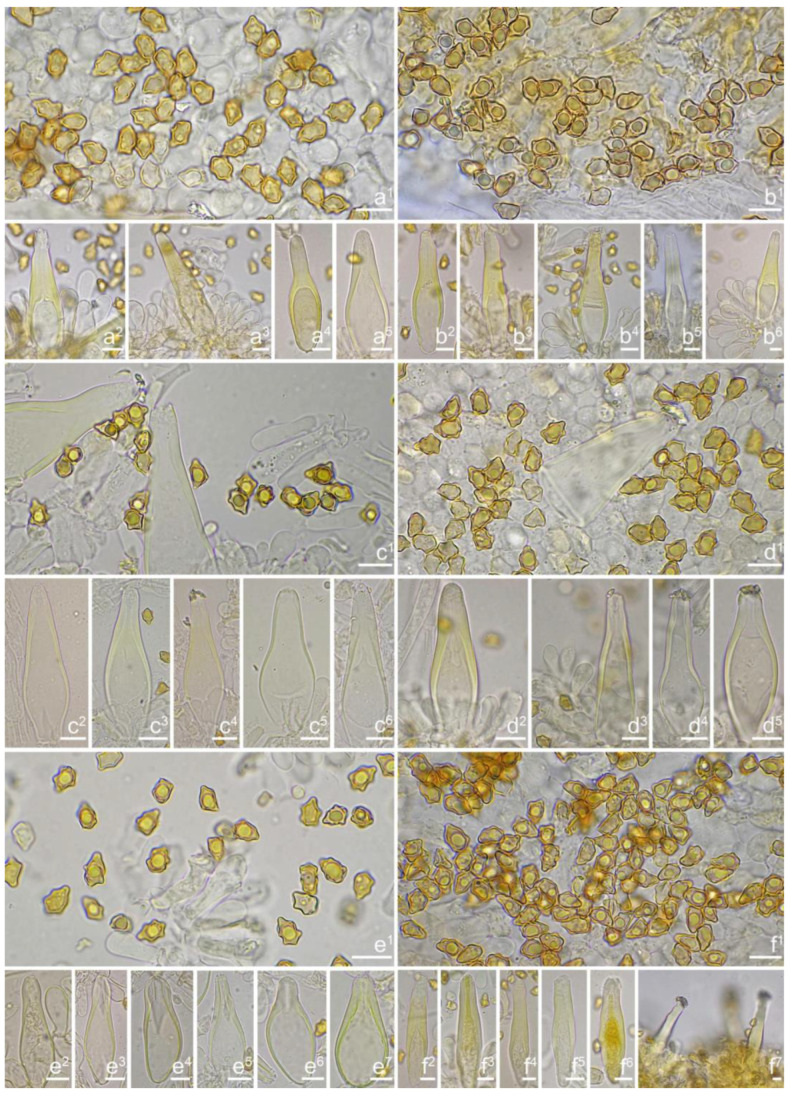
Basidiospores and hymenial cystidia of *Inocybe castanea.* (**a^1^**–**a^5^**) FYG3741 (FCAS3859); (**b^1^**–**b^6^**) FYG1677 (FCAS3857); (**c^1^**–**c^6^**) FYG1327 (FCAS3863); (**d^1^**–**d^5^**) FYG1803 (FCAS3856); (**e^1^**–**e^7^**) FYG1400 (FCAS3860); (**f^1^**–**f^7^**) FYG544-2020 (FCAS3854). Scale bars: 10 μm. Photos by X. Chen.

***Inocybe castanea*** Peck, Bull. N.Y. St. Mus. 75: 16, 1904 Figure 5, Figure 6 and Figure 7

*Basidiomata* small-sized. *Pileus* 13–20 mm in diameter, hemispherical when young, expanding to paraboloid to convex or plano-convex to applanate with a small umbo, margin initially decurved, then straight; surface dry, glabrous at first, then smooth-fibrillose to appressed-fibrillose or appressed scaly; uniformly brown (6C5) when young, then yellowish-brown (4B4), reddish-brown (6C8), paler toward the margin. *Lamellae* 1.8–2.2 mm broad, crowded, adnexed, length varies, alternatively distributed with 3–4 layers of lamellulae, edge uneven, not fimbriate; cream white (2A1) at first, then grayish-white (1C1) to yellowish-brown (5C7). *Stipe* 26–35 × 2–2.6 mm, central, solid, terete, equal with a slightly swollen base, surface covered with a layer of longitudinally arranged whitish (1A1) furfuraceous fibrils those easily fugacious when touched, pruinose at the stipe apex, pruinose-furfuraceous downward; brownish (5A4) to reddish-brown (6C7), pallid at the base. *Context* solid, fleshy in pileus, white (1A1) or cream white (2A1); fibrillose and striate in stipe, pinkish (11A2) or pinkish–brown (12B2), color unchanging where cut. ***Odor*** raw oil or mild.

*Basidiospores* [*n* = 100/15/15] (6.0) 6.1–7.3–8.1 (8.9) × (3.8) 4.2–5.1–6.0 (6.2) μm, Q = (1.08) 1.20–1.43–1.72 (1.86), Q_m_ ± SD = 1.43 ± 0.15, with 10–18 small nodules and a subconical knob at apices about an angular outline, pale yellow to yellowish-brown, apiculus indistinct, with intracellular circular to oblong oily inclusions. *Basidia* 23–29 × 7–9 μm, mostly claviform to broadly claviform, mostly 4-sterigmate, but 2-sterigmate basidia occasionally observed, sterigmata 2–5 μm in length. *Pleurocystidia* 47–64–88 × 13–19–25 μm (*n* = 40), abundant, mostly fusiform and broadly fusiform, occasionally lageniform, crystallization present at the apices, apices subconical to subobtuse, often covered with dense granules, base rounded to obtuse, occasionally tapering, thick-walled, walls up to 2.5 μm thick at the middle and strikingly thickened near the apex (up to 5 μm), pale yellow. *Cheilocystidia* 45–59–75 × 15–18–23 μm (*n* = 40), abundant, resemble pleurocystidia, mostly lageniform, fusiform or broadly fusiform, thick-walled, walls colorless to yellowish. *Cheiloparacystidia* 13–22 × 7–13 μm, broadly clavate or clavate, hyaline, colorless, thin-walled, smooth, mixed with numerous metuloidal cheilocystidia. *Caulocystidia* 58–65–79 × 15–17–20 μm (*n* = 20), abundant in stipe apex, sparser in the middle to stipe base, fusiform to lageniform, thick-walled, apices crystalliferous. *Cauloparacystidia* 10–24 × 10–22 μm, circular to ovoid, colorless, hyaline, doped growth with caulocystidia. *Hymenophoral trama* 400–530 μm thick, regularly arranged, colorless in mass, consisting of thin-walled, colorless, smooth, mostly inflated hyphae 7–16 μm wide, cylindrical hyphae occasionally observed. *Pileipellis* a cutis consisting of two layers, the upper layer made up of cylindrical hyphae, smooth, colorless, 3–9 μm wide, and lower layer brownish in color, subregularly arranged, composed of inflated pale yellowish, encrusted hyphae 11–25 μm wide. *Pileal trama* 450–630 μm thick, subregularly arranged, colorless, hyphae 8–25 μm wide, thin-walled, inflated. *Stipitipellis* regularly arranged, pale yellowish in color, composed of cylindrical hyphae, 3–10 μm wide, thin-walled, smooth. *Oleiferous hyphae* 1.5–6 µm wide, yellowish, diverticulate, mostly present in the pileal and the stipe trama. *Clamp connections* present and common in all tissues.

**Habitat**. in coniferous forests, mixed forests or in alpine *Dryas* communities.

**Distribution**. China (Jilin, Nei Mongol, and Yunnan), Europe, North America (type).

**Additional specimens examined**. China, Jilin Province: Yanbian Korean Autonomous Prefecture, Antu County, Erdaobaihe Town, Changbai Mountain Nature Reserve, 421′19″ N, 128°4′25″ E, in coniferous forests, 2 August 2017, Y.G. Fan, FYG1326 (FCAS3862), FYG1327 (FCAS3863); in mixed forests, 30 July 2019, Y.G. Fan & L.W. Qin, FYG3741 (FCAS3859); in coniferous forests, 17 August 2021, Y.P. Ge, GN1400 (FCAS3860), GN1412 (FCAS3855); Fushi Forest, in coniferous forests, 14 August 2017, Y.G. Fan & W.J. Yu, FYG1662 (FCAS3864), FYG1673 (FCAS3865), FYG1677 (FCAS3857); Bailong Power Station, in *Quercus* forests, 19 August 2017, Y.G. Fan & W.J. Yu, FYG1803 (FCAS3856); Shuangmufeng, 42°9′47″ N, 127°38′43″ E, 21 August 2020, Y.G. Fan & W.J. Yu, FYG3-2020 (FCAS3866), 25 August 2020, Y.G. Fan & W.J. Yu, FYG10-2020 (FCAS3853), FYG14-2020 (FCAS3858), 41°59′35″ N, 128°1′4″ E, 30 August 2020, Y.G. Fan & W.J. Yu, FYG544-2020 (FCAS3854); Nei Mongol Autonomous Region: Hulunbuir City, Genhe, Aoluguya Mountain, in *Larix* forests, 8 August 2022, T.Z. Liu & Y.M. Gao, CFSZ 25181, CFSZ 25182, CFSZ 25200, CFSZ 25177, CFSZ 25187; Yunnan Province, Chuxiong Yi Autonomous Prefecture, Chuxiong City, Zixi Mountain, 25°0′35″ N, 101°23′51″ E, 13 September 2015, in *Pinus* forests, Y.G. Fan, FYG2015149 (FCAS3852); Diqing Tibetan Autonomous Prefecture, Shangri La City, Habaxueshan Yi Ethnic Village, 27°23′4″ N, 100°6′22″ E, alt. 3214 m, in mixed forests dominated by *Pinus armandii* Franch., *Quercus*, and *Rhododendron decorum* Franch., 25 August 2020, H.Y. Huang & L.P. Tang, (Huang893-MHKMU).

**Remarks**. *Inocybe castanea* was originally discovered under spruce and balsam fir trees in New York (Lake Pleasant) [104] and has been found to be widespread in northern Europe and northeast Asia [41,45]. This species is characterized by a brown to reddish-brown pileus, whitish furfuraceous-pruinose stipes with a brownish background, yellowish lamellae before overmaturing, angular basidiospores with weak nodules, lageniform hymenial cystidia often with obtuse or rounded bases, and caulocystidia present at the stipe apex and descending to the stipe base. Phylogenetically, three phylogenetic lineages can be found in *I. castanea*, the Chinese materials nested in two of the three lineages, but no stable morphological characters were observed to distinguish the two lineages. The microscopic characteristics show some divergence in different specimens (Figure 7), e.g., the basidiospores of FYG3741 have no oily droplets, GN1400 has shorter hymenial cystidia, the hymenial cystidia of FYG544-2020 are subcylindrical with yellow walls; the apices of the hymenial cystidia are crystalliferous and show dense granular remnants after the dissolution of the crystals in 5% KOH, as shown in the line drawing of this species in Jacobsson (2008) [45], but this feature is not always obvious when examining more Chinese collections. Furthermore, *I. castanea* has been reported to occur in *Picea* forests and in subalpine *Betula* forests in Europe [41,45], whereas most Chinese specimens were collected in coniferous forests dominated by *Larix* or *Pinus*, with the exception of FYG544-2020, which occurred in *Dryas octopetala* var. *asiatica* vegetation in the alpine zone. In this study, a new distribution of *I. castanea* in Yunnan Province was confirmed based on two collections from subtropical mountainous mixed forests dominated by *Pinus*. One specimen BJ910825 labeled as *I. sapinea* by Ryberg et al. (2008) [72], clustered in the *I. castanea* clade in our phylogeny, which was obviously a misapplied name.

**Figure 8:**
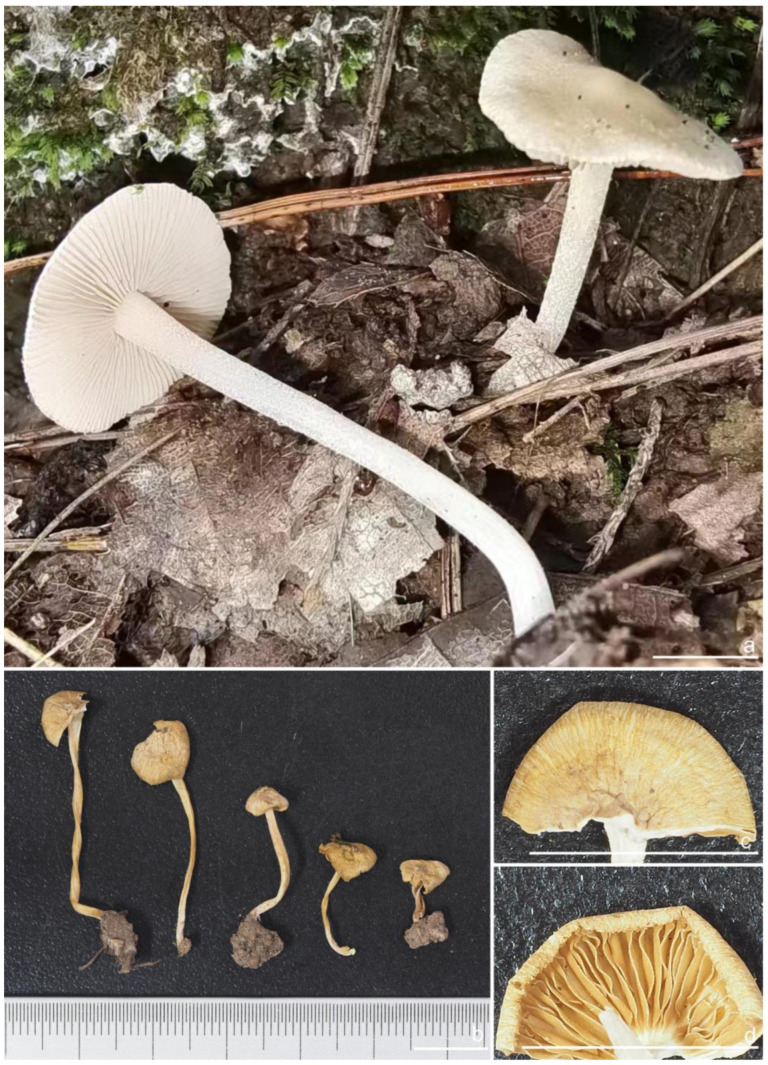
Basidiomata of *Inocybe dabaensis*. (**a**–**d**) YZ2023102844 (FCAS4009, holotype). Scale bars: 10 mm. Photos by X.-M. Yang and X. Chen.

**Figure 9:**
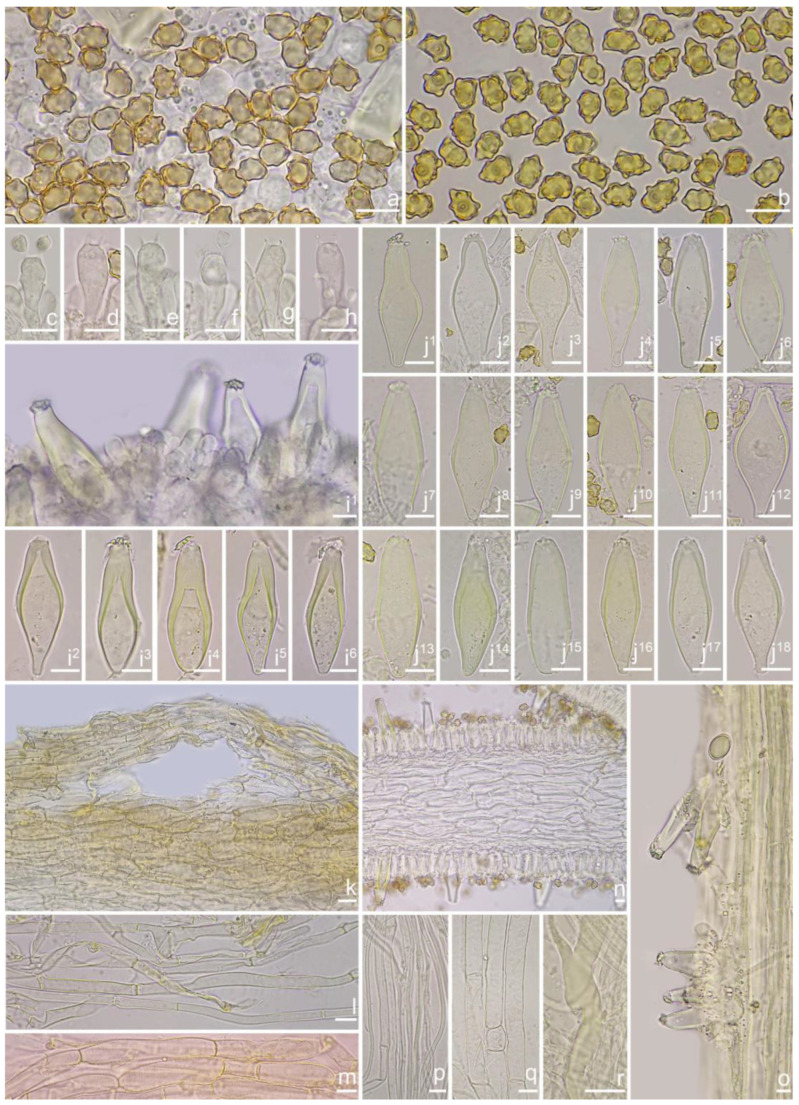
Microscopic features of *Inocybe dabaensis* (FCAS4009, holotype). (**a**,**b**) Basidiospores. (**c**–**h**) Basidia. (**i^1^**–**i^6^**) Cheilocystidia. (**j^1^**–**j^18^**) Pleurocystidia. (**k**) Pileipellis. (**l**) Pileipellis upper layer hyphae. (**m**) Pileipellis lower layer hyphae. (**n**) Cross-section of lamellae. (**o**) Stipitipellis with caulocystidia. (**p**) Stipitipellis hyphae. (**q**) Stipe trama hyphae. (**r**) Oleiferous hyphae. Scale bars: 10 μm. Photos by X. Chen.

***Inocybe dabaensis*** Y.G. Fan, X. Chen & X.M. Yang, sp. nov. Figure 8 and Figure 9.

**Chinese name**. 大巴丝盖伞

**MycoBank**. MB 855821.

**Etymology**. In reference to Daba Mountain (Sichuan Provence) where the type specimen was collected.

**Diagnosis**. *Inocybe dabaensis* have white to silver white basidiomata changing to straw color upon drying, close lamellae, angular–nodulose basidiospores, and broadly fusiform hymenial cystidia with tapered bases. Similar to *I. angustifolia* and *I. keteleeriicola*, but differs from the former by the less crowded lamellae, less distinct nodules in basidiospores, thinner walled cystidia and a different phylogenetic placement; differs from the latter by the longer basidiospores, two-spored basidia, thinner walls toward the base of the cystidia, and a different ecology.

**Holotype**. China, Sichuan Province: Dazhou City, Xuanhan County, Juntang Town, Xuanhan Village, in mixed forests dominated by *Pinus* and fagaceous trees, 1 November 2023, Xian-Mei Yang, YZ2023102844 (FCAS4009), GenBank accession number: ITS (PP993809); LSU (PP993852); RPB2 (PQ001020).

*Basidiomata* small to medium-sized. *Pileus* 18–30 mm in diameter, conic-convex to hemispherical when young, then plano-convex with indistinct umbo, applanate with a broad and obtuse umbo when mature, margin decurved when young, becoming depressed to straight upon maturity; surface dry, glabrous near the center, fibrillose to fibrillose-rimulose outward, finely subtomentose near the margin, margin often split or strongly split when overmature; uniformly ivory (1A2) to white (1A1), or grayish-white (1A3) when young, then yellowish white (2A2) to earthy yellow (2B2) or dirty white (1A2). *Lamellae* 1.5–2.5 mm deep, adnexed, close, alternately distributed with 3–4 layers of lamellulae, initially whitish (1A1), becoming yellowish-white (1A3) when mature, edge entire to finely crenulated. *Stipe* 30–65 × 3–5 mm, central, solid, terete, often slightly swollen at the base, white (1A1) pruinose along the entire length, longitudinally striate; surface white (1A1), ivory white (23A1) to yellowish-white (1A3). *Context* fleshy in pileus, 0.5 mm thick at mid-radius, up to 3 mm thick under the umbo, cream white (2A1), fibrillose in stipe, striate, cream white (2A1). Odor not recorded.

*Basidiospores* [*n* = 100/3/3] (7.2) 7.5–8.5–9.2 (10.1) × (5.0) 5.1–5.8–6.2 (7.1) μm, Q = (1.11) 1.24–1.43–1.71 (1.87), Qm ± SD = 1.43 ± 0.13, with 8–16 small nodules on an angular outline, pale yellowish to yellowish-brown in 5% KOH, apiculus indistinct, at times with one intracellular circular to oblong oil inclusions. *Basidia* 16–28 × 6–9 μm, mostly claviform to broadly claviform, hyaline, mostly 2-spored, but 3-spored basidia occasionally observed, sterigmata 2–5 μm in length. *Pleurocystidia* 42–50–64 × 13–16–21 μm (*n* = 40), mostly fusiform to broadly fusiform, at times cylindrical, apically crystalliferous, apices obtuse or subobtuse, base rounded to obtuse, occasionally tapered into small pedicel, thick-walled, walls up to 3 μm thick at the middle and noticeably thickened near the apex (up to 4), pale greenish-yellow, extremely abundant. *Cheilocystidia* 38–45–52 × 12–14–17 μm (*n* = 40), resemble pleurocystidia, mostly fusiform or broadly fusiform, occasionally lageniform, thick-walled, walls less than 4 μm thick. *Cheiloparacystidia* 12–17 × 5–7 μm, clavate to ovoid, hyaline, thin-walled, smooth, mixed with numerous metuloidal cheilocystidia. *Caulocystidia* 35–45–54 × 12–14–20 μm (*n* = 40), mostly fusiform, lageniform or broadly fusiform, abundant and descending the entire length of the stipe, thick-walled, walls up to 3 μm thick, colorless to pale yellowish, crystalliferous at the apices. *Cauloparacystidia* 12–25 × 5–12 μm, oblong, ovoid or clavate, thin-walled, colorless, mixed with caulocystidia. *Hymenophoral trama* 85–130 μm thick, subregularly arranged, colorless, composed of thin-walled, smooth, mostly inflated and cylindrical hyphae, 3–18 μm wide. *Pileipellis* divided into two layers, 100–150 μm wide, upper layer interwoven, pale yellow to yellow, hyphae cylindrical, smooth, thin-walled, 3–7 μm wide; lower layer regularly arranged, composed of yellow to yellowish-brown, encrusted, subinflated hyphae 9–20 μm wide. *Pileal trama* regularly arranged, composed of inflated and cylindrical hyphae up to 20 μm in diameter, colorless, smooth, thin-walled. *Stipitipellis* a cutis composed of pale yellowish, cylindrical hyphae 2–5 μm wide, thin-walled, smooth. *Oleiferous hyphae* 1.5–9 µm wide, nearly colorless, smooth, mostly present in stipe trama. *Clamp connections* present and common in all the tissues.

**Habitat**. In mixed forests dominated by fagaceous trees.

**Distribution**. China (Sichuan), Japan.

**Additional specimens examined**. China, Sichuan Province: Dazhou City, Xuanhan County, Juntang Town, Xuanhan Village, in mixed forests dominated by fagaceous trees and *Pinus,* 1 November 2024, Xian-Mei Yang, YZ2023102844-1 (FCAS4020), YZ2023102844-2 (FCAS4021).

**Remarks**. *Inocybe dabaensis* is characterized by its small, white basidiomata, crowded lamellae, slender stipes, angular–nodulose basidiospores, two-spored basidia, and fusiform hymenial cystidia. It occurs in October at the type locality. The is morphologically highly similar to *I. angustifolia*, but the latter has more crowded lamellae [54], less prominent nodules in basidiospores and thicker cystidia walls (inferred from our observations based on Chinese collections), and a different phylogenetic placement. *Inocybe keteleeriicola* is phylogenetically close to *I. dabaensis*, but the former has less prominent nodules in basidiospores and thicker-walled hymenial cystidia, and occurs in *Keteleeria* forests. Among the nodulose-spored *Inocybe* species, only two species with two-spored basidia are known, viz. *I. aberrans* (E. Horak) Garrido described from Papua New Guinea, and *I. miyiensis* T. Bau & Y.G. Fan described from subtropical China. *Inocybe aberrans* has campanulate pileus covered with squarrose scales, larger stellate basidiospores, thin-walled hymenial cystidia, and occurring in Nothofagus forests in Papua New Guinea [52]; *I. miyiensis* has more yellow tint in the pileus, pallid stipes, conspicuously enlarged and papilla-like sterigmata, and stellate to substellate basidiospores. An unpublished ITS sequence generated from ectomycorrhizal root tip (402 TKB mateba) clusters in *I. dabaensis*, indicates its ecological association with *Lithocarpus* in Japan.

**Figure 10:**
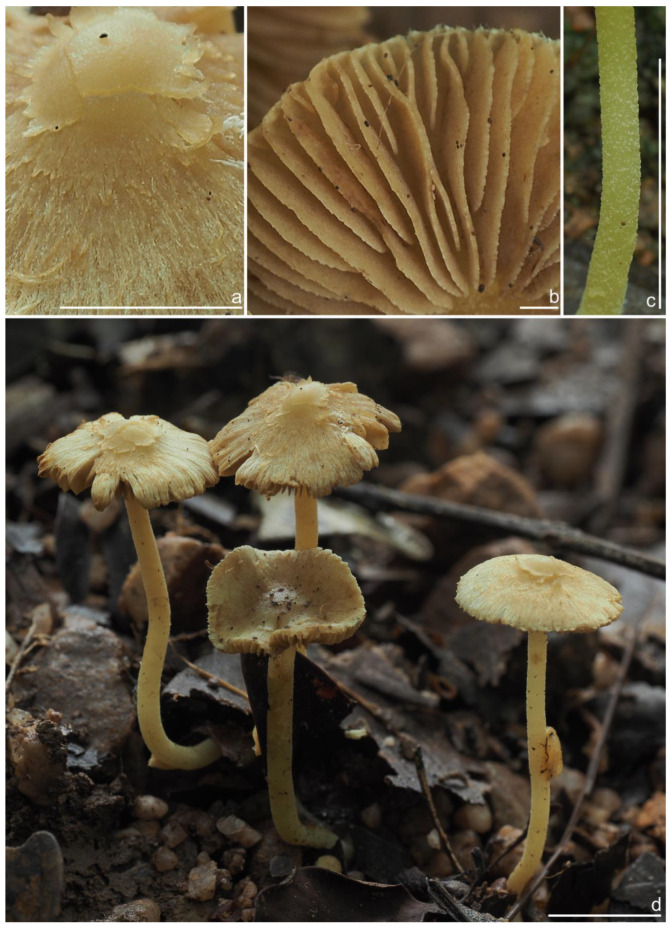
Basidiomata of *Inocybe danxiaensis*. (**a**,**b**,**d**) FYG4389 (FCAS3837, holotype); (**c**) FYG4392 (FCAS3838). Scale bars: (**a**) 0.5 mm; (**b**) 1 mm; (**c**,**d**) 10 mm. Photos by Y.-G. Fan.

**Figure 11:**
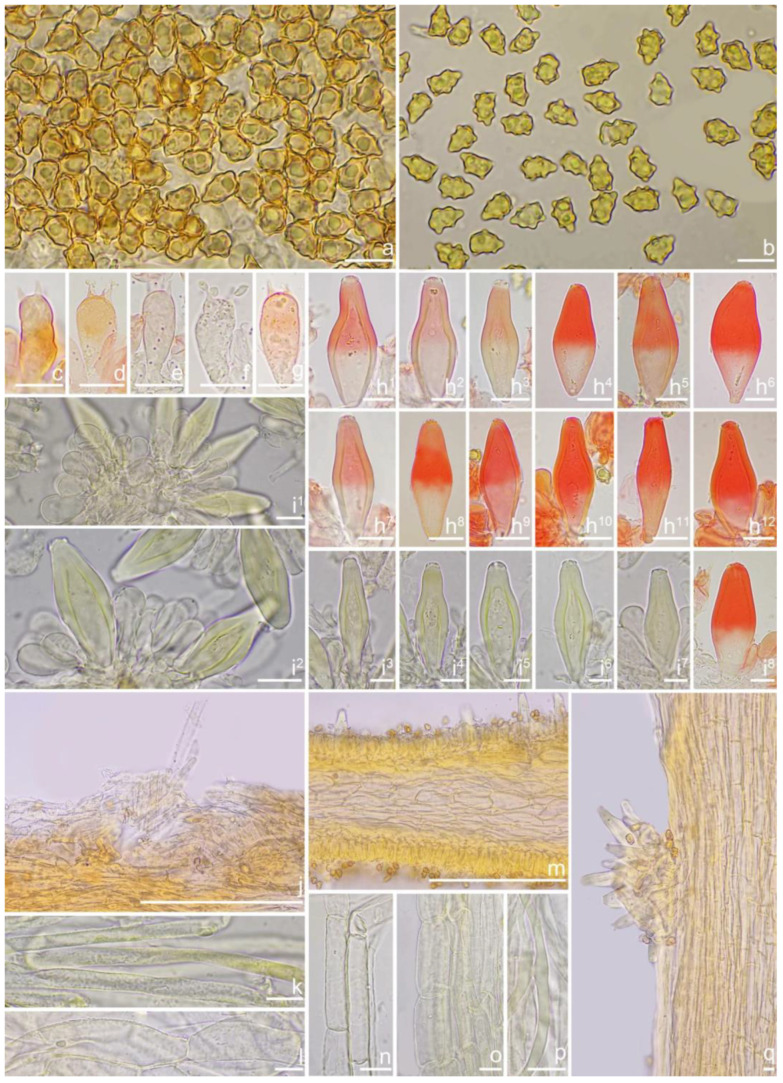
Microscopic features of *Inocybe danxiaensis* (FCAS3837, holotype). (**a**,**b**) Basidiospores. (**c**–**g**) Basidia. (**h^1^**–**h^12^**) Pleurocystidia. (**i^1^**–**i^8^**) Cheilocystidia and cheiloparacystidia. (**j**) Pileipellis. (**k**) Pileipellis upper layer hyphae. (**l**) Pileipellis lower layer hyphae. (**m**) Cross-section of lamellae. (**n**) Stipitipellis hyphae. (**o**) Stipe trama hyphae. (**p**) Oleiferous hyphae. (**q**) Stipitipellis with caulocystidia. Scale bars: (**a**–**i^8^**,**k**–**l**,**n**–**q**) 10 μm, (**j**,**m**) 100 μm. Photos by X. Chen.

***Inocybe danxiaensis*** Y.G. Fan, W.J. Yu & X. Chen, sp. nov. Figure 10 and Figure 11.

**Chinese name**. 丹霞丝盖伞.

**MycoBank**. MB 855822.

**Etymology**. In reference to Danxia Mountain (Guangdong Province), where the type specimen was collected.

**Diagnosis**. *Inocybe danxiaensis* has noticeable dull yellow to greenish-yellow basidiomata with a slender habit, angular–nodulose basidiospores, and thick-walled hymenial cystidia. Most similar to *I. paludinelloides*, but differs from it by the fusiform hymenial cystidia with thicker walls and a sweet smell.

**Holotype**. China, Guangdong Province, Shaoguan City, Danxia Mountain, in *Fagaceae* forests, 25°1′45″ N, 113°43′35″ E, 3 June 2019, Y.G. Fan, FYG4389 (FCAS3837), GenBank accession number: ITS (PP217735); LSU (PP230774).

*Basidiomata* small, slender. *Pileus* 13–17 mm in diameter, hemispherical at first, then plano-convex with indistinct umbo, uplifted and non-umbonate when overmature; margin initially depressed, then straight; surface dry, glabrous when young, becoming radially fibrillose to fibrillose–rimulose, rimulose or cracked toward the margin when overmature; yellowish (3A3) to yellow (3A7) when young, dirty yellow (2A3) to brownish-yellow (5B6) when mature. *Lamellae* 1.0–2.5 mm deep, adnexed, subcrowded to crowded, unequal in length, alternately distributed with 2–3 tiers of lamellulae; cream (1A2) to cream yellow (3A2) at first, yellow (1A7) to brownish (5A2) with age; edge finely crenulated. *Stipe* 23–55 × 1.2–1.8 mm, terete, equal, solid, terete, equal or swollen at base, pruinose along the entire length of the stipe, yellowish-white (2A2) to greenish-yellow (27A4) when young, pale yellow (2A3) to brownish (5B3) when overmature. *Context* fleshy in pileus, cream white (23A1) to lightly yellowish (1A7), 1.0–1.5 mm thick under the umbo, 0.3–0.7 mm thick at mid-radius; shiny and fibrillose in stipe, striate. *Odor* fungoid or sweet.

*Basidiospores* [100/2/2] (5.9) 6.9–7.9–8.6 (9.5) × (4.2) 4.8–5.1–5.9 (6.5) μm, Q = (1.21) 1.32–1.47–1.71 (1.93), Q_m_ ± SD = 1.47 ± 0.12, angular with 10–14 small nodules, apiculus indistinct, with a prominent conical knob at apex, pale yellowish to yellow, with one or more intracellular circular to oblong oil inclusions. *Basidia* 20–28 × 6–11 μm, mostly claviform, broadly claviform or ventricose, hyaline, mostly 4-spored, sterigmata 2–5 μm in length. *Pleurocystidia* 35–45–54 × 11–14–19 μm (*n* = 40), mostly fusiform, broadly fusiform, or lageniform, crystallization present at the obtuse apices, base nearly rounded to subtruncate, thick-walled, walls up to 3 μm wide, yellowish, extremely abundant. ***Cheilocystidia*** 34–45–61 × 11–17–22 μm (*n* = 40), resemble pleurocystidia, mostly fusiform, lageniform or broadly fusiform. *Cheiloparacystidia* 13–20 × 6–10 μm, clavate to broadly clavate, smooth, hyaline, thin-walled, mixed with numerous metuloidal cheilocystidia. *Caulocystidia* 30–46–68 × 12–15–17 μm (*n* = 20), mostly fusiform, lageniform or broadly fusiform, at times ventricose, distributed the entire stipe length, thick-walled, walls up to 3 μm thick, pale yellow. *Cauloparacystidia* 13–21 × 7–12 μm, oblong or clavate, thin-walled, colorless, mixed with caulocystidia. *Hymenophoral trama* 50–113 μm thick, subregularly arranged, colorless, composed of thin-walled, colorless, smooth, mostly inflated and cylindrical hyphae, 4–21 μm wide. *Pileipellis* divided into two layers, 90–180 μm wide, upper layer interwoven, colorless, hyphae cylindrical, rough, thin-walled, not hyaline, at times oily yellow, 3–7 μm wide; lower layer regularly arranged, pale yellow–brownish, rough, hyphae 8–17 μm wide. *Pileal trama* regularly arranged, composed of inflated hyphae up to 20 μm in diameter, colorless, smooth, thin-walled. *Stipitipellis* a cutis composed of pale yellow, cylindrical hyphae, thin-walled 2–9 μm wide, smooth. *Oleiferous hyphae* 2–5 µm wide, colorless, diverticulate, mostly present in stipe and hymenophoral trama. *Clamp connections* present and common in all tissues.

**Habitat**. On sandy soil in *Fagaceae* forest.

**Distribution**. China (Guangdong).

**Additional specimens examined**. China, Guangdong Province, Shaoguan City, Renhua County, Danxia Mountain, in *Fagaceae* forests, 3 June 2019, Y.G. Fan, FYG4392 (FCAS3838).

**Remarks**. *Inocybe danxiaensis* has small slender and yellowish basidiomata, angular–nodulose basidiospores with a prominent apical knob, thick-walled hymenial cystidia, and a sweet smell. It occurs singly or in clusters in early June under *Fagaceae* forests in southern China. The yellowish basidiomata make the new species impressive in the field. *Inocybe agenteolutea* Vauras described from Finland is similar to the new species in having yellowish-tinged basidiomata, nodulose basidiospores, and thick-walled hymenial cystidia, but the former has abundant silvery velipellis, shorter stipes, larger basidiospores with fewer (7–9) nodules, longer and narrower hymenial cystidia, occurs in mountain birch forest and probably associated with *Betula* or *Salix* [105]. *Inocybe paludinella* shares yellowish basidiomata, but it differs by the white to straw-yellow pileus, thicker walled hymenial cystidia, and an ecology under conifers or *Alnus* and *Salix* [37,38,39]. *Inocybe subangustifolia* is highly similar to the new species in external appearance; but it has basidiospores with less pronounced knobs, narrower hymenial cystidia, an ecology on sand or soil under *Eucalyptus* or *Acacia* [51]*,* and a different phylogenetic position*. Inocybe palludinelloides* and *I. spectabilis*, both described in the present work, are superficially similar to *I. danxiaensis*, but both species have sublageniform to lageniform hymenial cystidia. Phylogenetically, *I. danxiaensis* is a sister to *I. luxiensis*, which also has a sweet smell, but the latter has grayish-white to ivory white basidiomata, more pronounced nodules in basidiospores, and thicker-walled hymenial cystidia.

**Figure 12:**
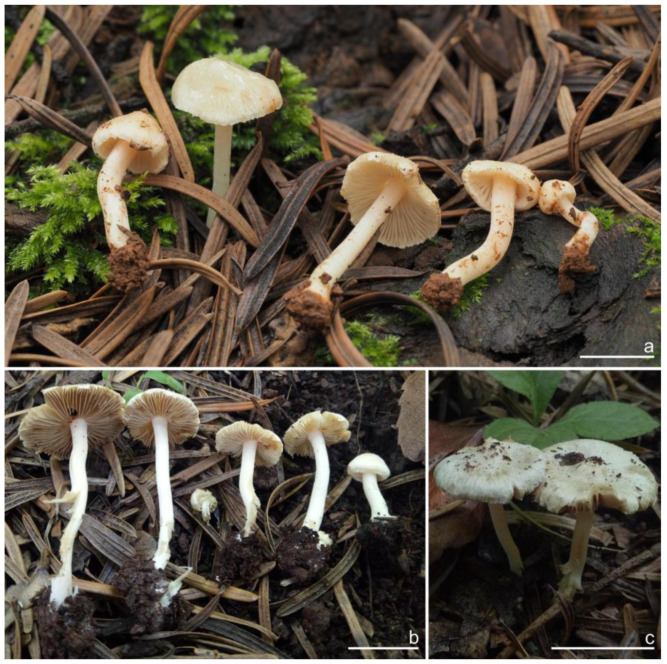
Basidiomata of *Inocybe keteleeriicola*. (**a**) FYG2917 (FCAS4008); (**b**,**c**) FYG2884 (FCAS3831, holotype); Scale bars: 10 mm, Photos by Y.-G. Fan.

**Figure 13:**
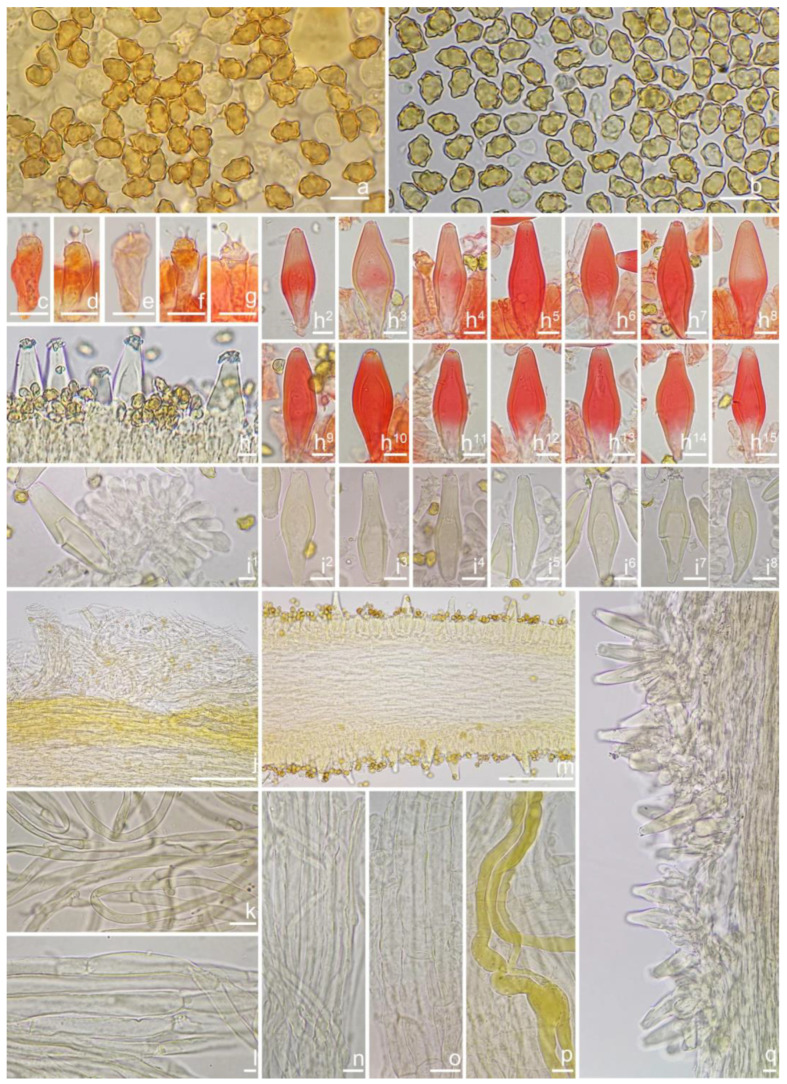
Microscopic features of *Inocybe keteleeriicola* (FCAS3831, holotype). (**a**,**b**) Basidiospores. (**c**–**g**) Basidia. (**h^1^**–**h^15^**) Pleurocystidia. (**i^1^**–**i^8^**) Cheilocystidia. (**j**) Pileipellis. (**k**) Pileipellis upper hyphae. (**l**) Pileipellis lower layer hyphae. (**m**) Cross-section of lamellae (**n**) Stipitipellis hyphae. (**o**) Stipe trama hyphae. (**p**) Oleiferous hyphae. (**q**) Stipitipellis with numerous caulocystidia. Scale bars: (**a**–**i^8^**,**k**–**l**,**n**–**q**) 10 μm, (**j**,**m**) 100 μm. Photos by X. Chen.

***Inocybe keteleeriicola*** Y.G. Fan, W.J. Yu & X. Chen, sp. nov. Figure 12 and Figure 13.

**Chinese name**. 油杉丝盖伞.

**MycoBank**. MB 855823.

**Etymology**. In reference to its putative host plant *Keteleeria.*

**Diagnosis**. *Inocybe keteleeriicola* has small white basidiomata, crowded lamellae, entirely pruinose stipe, nodulose basidiospores, and thick-walled hymenial cystidia. Most similar to *I. angustifolia*, but differs by smaller basidiomata, conspicuously less crowded lamellae, an equal stipe with non-marginate base, and an association with *Keteleeria*.

**Holotype**. China, Yunnan Province, Kunming City, Xi Mountain, in *Keteleeria* forests, 24°51′24″ N, 102°37′28″ E, 12 July 2018, Y.G. Fan & W.J. Yu FYG2884 (FCAS3831), GenBank accession number: ITS (PP217732); LSU (PP230769) and RPB2 (PP238448).

*Basidiomata* small-sized. *Pileus* 7–17 mm in diameter, hemispherical to conic-convex when young, plano-convex applanate with inconspicuous umbo when mature; margin initially somewhat incurved, then decurved for a long time, crenulated and straight when mature; surface dry, viscid when moist, wooly fibrillose to fibrillose-rimulose; uniformly dirty-white (1A2) to ivory white (23A1), at times ochraceous white (5A2). *Lamellae* 1.2–2.2 mm deep, adnexed to sinuate, crowded, unequal in length, alternately distributed with 3–4 tiers of lamellulae, white (1A1) or cream white (2A1) when young, beige (1B1), brownish-gray (5B2) to yellowish-brown (5A2) upon maturity. *Stipe* 20–37 × 2–3 mm, terete, solid at first and tending to be fistulose when mature, equal with occasionally slightly swollen base, base with white tomentose hyphae, entirely pruinose, longitudinally striate; white (1A1) to cream white (2A1), ivory white (23A1). *Context* fleshy in pileus, 0.2–1 mm thick at mid-radius, up to 2 mm thick under the umbo, white (1A1) or dirty white (1A2), becoming slightly brownish (4B3) with age; fibrillose and shiny in the stipe, whitish (1A1) to beige (1B1). *Odor* spermatic or somewhat sweet.

*Basidiospores* [100/3/3] (5.6) 6.2–7.5–8.2 (9.0) × (4.1) 4.2–5.2–6.1 (6.9) μm, Q = (1.12) 1.21–1.42–1.63 (1.81), Q_m_ ± SD = 1.42 ± 0.13, with 8–12 small nodules on an angular outline, pale yellowish to yellow–brown, apiculus indistinct, at times with one intracellular circular to oblong oil inclusions. *Basidia* 18–27 × 7–9 μm, mostly claviform, broadly claviform or ventricose, hyaline, mostly 4-sterigmate, but 2-sterigmate basidia occasionally observed, sterigmata 2–6 μm in length. *Pleurocystidia* 42–48–56 × 12–15–18 μm (*n* = 40), mostly fusiform to lageniform, or utriform, crystallization present at the apices, thick-walled, walls up to 3 μm thick, colorless to pale yellowish, extremely abundant. *Cheilocystidia* 34–44–55 × 12–15–19 μm (*n* = 40), resemble pleurocystidia, mostly fusiform, lageniform or broadly fusiform, occasionally (sub)utriform, tapering toward the base, base sometimes nearly rounded to subtruncate. *Cheiloparacystidia* 15–21 × 6–9 μm, clavate to ovoid, hyaline, thin-walled, smooth, mixed with numerous metuloidal cheilocystidia. *Caulocystidia* 36–50–71 × 13–16–19 μm (*n* = 20), mostly fusiform, lageniform or broadly fusiform, abundant, descending to the entire stipe length, thick-walled, walls up to 3 μm thick, colorless, crystallization present at the apices. *Cauloparacystidia* 9–25 × 6–13 μm, oblong, ovoid or clavate, thin-walled, colorless, mixed with caulocystidia. *Hymenophoral trama* 75–160 μm thick, subregularly arranged, colorless, composed of thin-walled, colorless, smooth, mostly inflated and cylindrical hyphae 3–18 μm wide. *Pileipellis* divided into two layers, 80–160 μm wide, upper layer interwoven, colorless but not hyaline, hyphae cylindrical, smooth, thin-walled, 3–8 μm wide; lower layer regularly arranged, goldish-yellow in mass, hyphae inflated, finely encrusted, 8–17 μm wide, yellowish. *Pileal trama* regularly arranged, composed of inflated hyphae up to 27 μm in diameter, colorless, smooth, thin-walled. *Stipitipellis* a cutis composed of thin-walled, cylindrical hyphae, 2–10 μm wide, rough. *Oleiferous hyphae* 3–10 µm wide, yellowish to yellow, diverticulate, mostly present in stipe trama. *Clamp connections* present and common in all tissues.

**Habitat and Phenology**. In natural and urban *Keteleeria* (*Pinaceae*) forests, late June to October in subtropical China.

**Distribution**. China (Yunnan).

**Additional specimens examined**. China, Yunnan Province, Kunming City, Xishan District, Xi Mountain, under *Keteleeria* trees, 24°51′24″ N, 102°37′28″ E, 12 July 2018, Y.G. Fan & W.J. Yu, FYG2917 (FCAS4008), FYG2918 (FCAS3832); Longquan Mountain, Heilongtan Park, under *Keteleeria* trees, 26 September 2015, Y.G. Fan & W.J. Yu, FYG2015409 (FCAS4011).

**Remarks**. *Inocybe keteleeriicola* was found at two sites in Kunming City in *Keteleeria* forests. This species is characterized by the small white basidiomata, crowded lamellae, entirely pruinose stipe, nodulose basidiospores, and thick-walled hymenial- and caulocystidia. In the three-gene phylogeny, *I. keteleeriicola* is closely related to *I. angustifolia* and *I. subangustifolia*. *Inocybe angustifolia*, originally described from Papua New Guinea (type) and found in tropical Asia (Indonesia, Malaysia, northwestern Thailand) is superficially similar to *I. keteleeriicola*, but the former has larger basidiomata, more crowded lamellae, more prominent nodules in basidiospores, and an ecology under fagaceous trees [52,54]. *Inocybe subangustifolia* is a small dull-yellow to greenish-yellow colored species described from Australia that is highly similar to *I. keteleeriicola* in basidiospores shape and size, but the former has yellowish basidiomata, less fusiform hymenial cystidia, and an association with *Eucalyptus* or *Acacia* [51].

**Figure 14:**
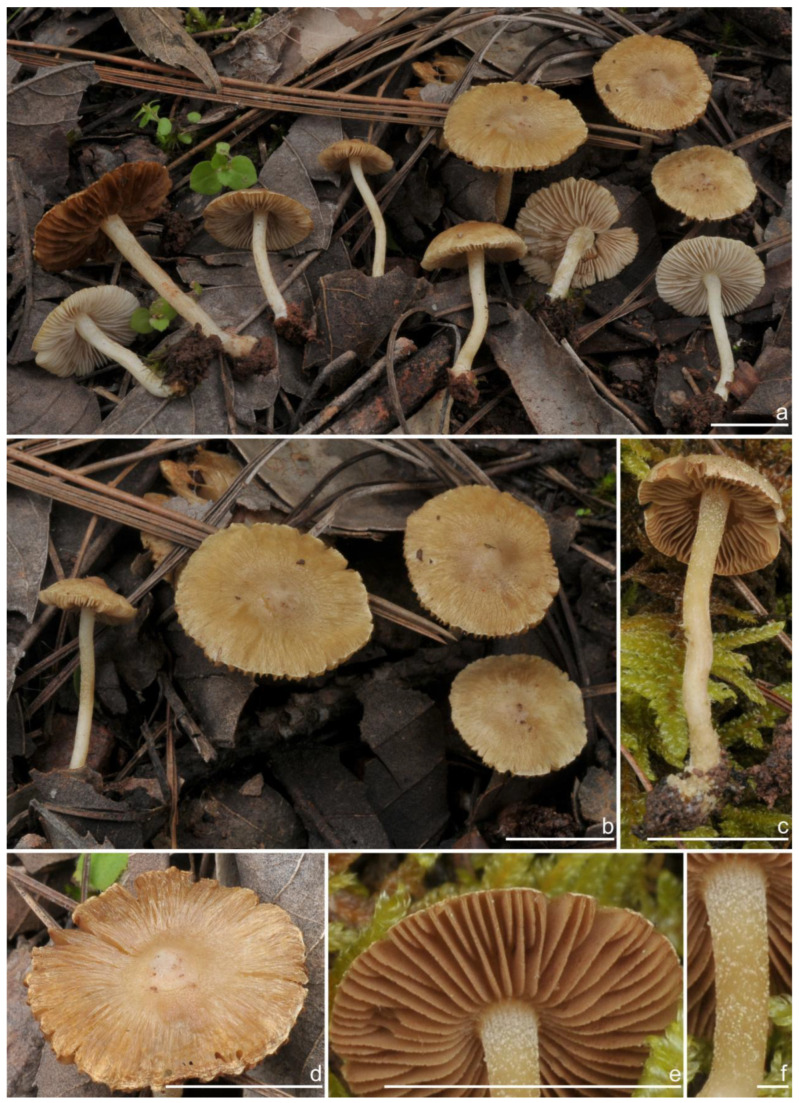
Basidiomata of *Inocybe lutosa.* (**a**,**b**,**d**) FYG2015298 (FCAS4042, holotype); (**c**,**e**,**f**) FYG2015290 (FCAS4043). Scale bars: (**a**–**e**) 10 mm; (**f**) 1 mm. Photos by Y.-G. Fan.

**Figure 15:**
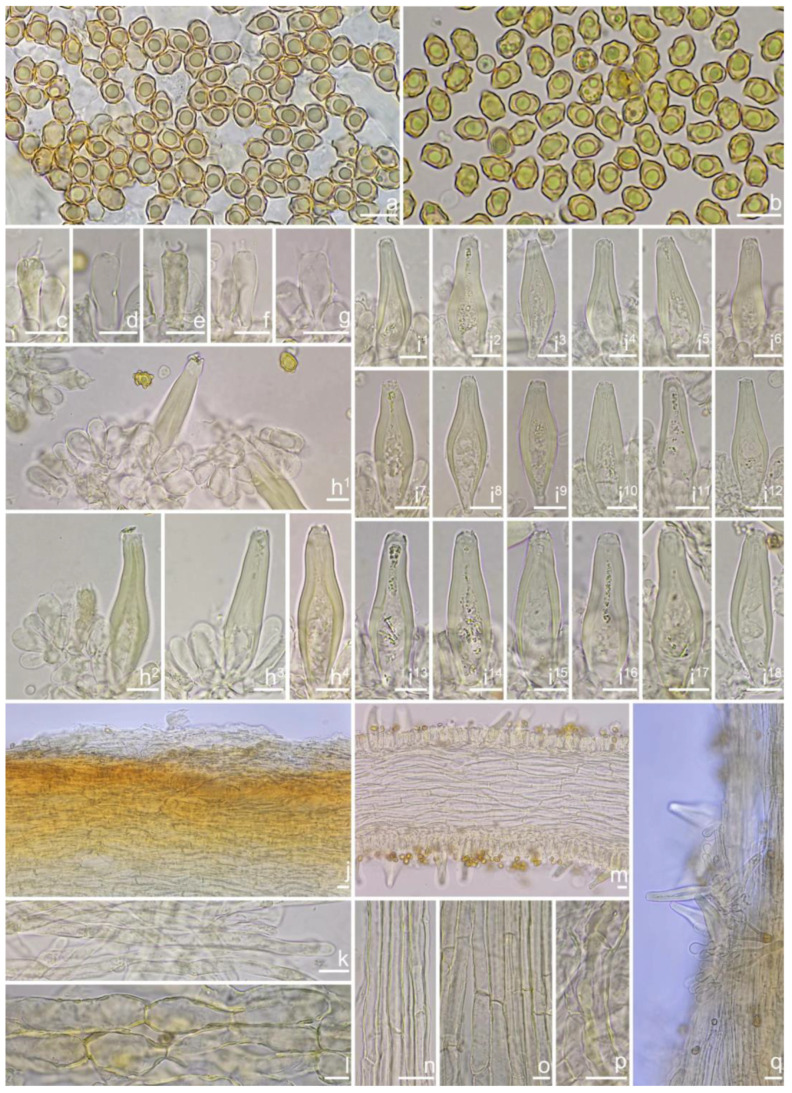
Microscopic features of *Inocybe lutosa* (FCAS4042, holotype). (**a**,**b**) Basidiospores. (**c**–**g**) Basidia. (**h^1^**–**h^4^**) Cheilocystidia and cheiloparacystidia. (**i^1^**–**i^18^**) Pleurocystidia. (**j**) Pileipellis. (**k**) Pileipellis upper hyphae. (**l**) Pileipellis lower hyphae. (**m**) Cross-section of lamellae (**n**) Stipitipellis hyphae. (**o**) Stipe trama hyphae. (**p**) Oleiferous hyphae. (**q**) Stipitipellis with caulocystidia. Scale bars: 10 μm. Photos by X. Chen.

***Inocybe lutosa*** Y.G. Fan, W.J. Yu & X. Chen, sp. nov. Figure 14 and Figure 15.

**Chinese name**. 泥泞丝盖伞.

**MycoBank**. MB 855824.

**Etymology**. Lutosus (Latin) means dirty or muddy, in reference to the earthy yellow color of the pileus color.

**Diagnosis***. Inocybe lutosa* has small basidiomata, earthy yellow pileus with cracked margin, subcrowded to crowded lamellae, entirely pruinose stipe, angular–nodulose basidiospores, and thick-walled hymenial cystidia. Most similar to *I. ailaoensis*, but differs from it by the earthy yellow pileus, indistinct nodules in basidiospores, and non-hyaline hyphae in the upper layer of pileipellis.

**Holotype**. China, Yunnan Province, Dali Yi Autonomous Prefecture, roadside of Highway 277, in fagaceous forests, 19 September 2015, Y.G. Fan & W.J. Yu, FYG2015298 (FCAS4042), GenBank accession number: ITS (PP993814); LSU (PP993851).

*Basidiomata* small-sized. *Pileus* 8–20 mm in diameter, conical to hemispherical when young, then expanded to plano-convex with indistinct umbo when mature; margin initially depressed, then straight; surface dry, near smooth at the disk, fibrillose to radially fibrillose-rimulose outward, usually finely split or strongly so toward the margin; earthy yellow (4C4) to pale yellowish (4B4) when young, then straw yellow (3A3) to ginger (4B4). *Lamellae* 1.5–2.0 mm broad, crowded to subcrowded, adnexed, length varies, alternatively distributed with 3–4 layers of lamellulae, edge uneven to crenulated, at times with pallid edges; yellowish-white (2A2) at first, then brownish-yellow (5D6). *Stipe* 22–34 × 1.2–2.2 mm, central, solid, equal with a swollen base, surface entirely pruinose, densely so at apices, sparser downward; pallid (1A1) at first, pale earthy yellowish (4C4) to beige (1B1) when mature. *Context* fleshy in pileus, 0.5 mm thick at mid-radius, up to 2.5 mm thick under the umbo, white (1A1); fibrillose and striate in stipe, cream white (2A1) to beige (1B1). *Odor* spermatic and more or less aromatic.

*Basidiospores* [100/3/3] (6.1) 6.5–7.1–7.9 (8.1) × (4.2) 4.8–5.0–5.5 (6.0) μm, Q = (1.13) 1.19–1.42–1.64 (1.77), Q_m_ ± SD = 1.42 ± 0.08, angular–nodulose with 6–12 small and inconspicuous nodules, apiculus small and indistinct, yellowish to dull goldish-yellow, with one intracellular circular to amorphous oily droplet. *Basidia* 17–23 × 6–8 μm, mostly claviform to narrowly claviform, thin-walled, mostly 4-spored, but 2-spored basidia occasionally observed, sterigmata 3–6 μm long. *Pleurocystidia* 42–46–54 × 10–13–16 μm (*n* = 40), abundant, mostly fusiform and lageniform, occasionally broadly fusiform, crystallization present at the obtuse apices, base nearly rounded to obtuse, occasionally tapered into small pedicel, thick-walled, walls up to 2.5 μm wide at the middle and strikingly thickened near the apex (up to 4 μm), colorless to pale yellowish. *Cheilocystidia* 37–45–55 × 12–14–15 μm (*n* = 40), abundant, resemble pleurocystidia, mostly fusiform, broadly fusiform and less often sublageniform; *Cheiloparacystidia* 12–17 × 6–8 μm, clavate to oblong, hyaline, colorless, thin-walled, mixed with cheilocystidia and occasionally basidia. *Caulocystidia* 37–45–61 × 10–13–18 μm (*n* = 40), abundant, descending to stipe base, fusiform, sublageniform, broadly lageniform or subcylindrical, thick-walled, apically crystalliferous; *Cauloparacystidia* 10–20 × 6–9 μm, abundant, ovoid, clavate, or oblong, thin-walled, colorless, mixed with caulocystidia. *Hymenophoral trama* 100–200 μm thick, regularly arranged, hyphae thin-walled, colorless, smooth, slightly inflated, 5–25 μm wide, cylindrical hyphae occasionally observed. *Pileipellis* a cutis of two layers, 100–240 μm wide, upper layer interwoven, colorless to pale greenish, hyphae 3–7 μm wide, cylindrical, smooth, thin-walled, not hyaline; lower layer subregularly arranged, hyphae inflated to subcylindrical, 5–17 μm wide, yellowish, inner walls unevenly thickened, up to 1 μm thick. *Pileal trama* 230–430 μm thick, subregularly arranged, colorless, hyphae 8–22 μm wide, smooth, thin-walled, inflated. *Stipitipellis* a cutis regularly arranged, pale yellowish in color, composed of cylindrical hyphae, 3–5 μm wide, thin-walled, smooth. *Oleiferous hyphae* 1–5 µm wide, greenish-yellow, diverticulate, mostly present in stipe trama. *Clamp connections* present and common in all tissues.

**Habitat**. Scattered in fagaceous forests, around September in subtropical China.

**Distribution**. China (Yunnan).

**Additional specimens examined**. China, Yunnan Province, Dali Yi Autonomous Prefecture, roadside of Highway 227, in fagaceous forests, 19 September 2015, Y.G. Fan & W.J. Yu, FYG215290 (FCAS4043), FYG215298a (FCAS4047), FYG215298b (FCAS4048), FYG215298c (FCAS4049).

**Remarks**. *Inocybe lutosa* is found in Yunnan Province in fagaceous forests. It is characterized by a small earthy-yellow pileus, entirely pruinose stipe, angular–nodulose basidiospores with indistinct nodules, and thick-walled hymenial cystidia. Phylogenetically, *I. lutosa* is a sister to *I. ailaoensis*, but the latter has white to silver–white pileus, more pronounced nodules on basidiospores, hyaline pileipellis hyphae in the upper layer, and more obtuse bases in hymenial cystidia. Sequence comparison of between the two species revealed 4.89% differences (31/634) in ITS including 12 base pairs and 6 insertion-deletion events (2–10 bp in length), 0.15% differences (2/1301) in LSU, and 1.32% differences (8/602) in *rpb2.* Another close relative is *Inocybe luxiensis*, which has indistinct yellowish basidiomata, larger and more pronounced rounded nodules in basidiospores, and thinner-walled hymenial cystidia.

**Figure 16:**
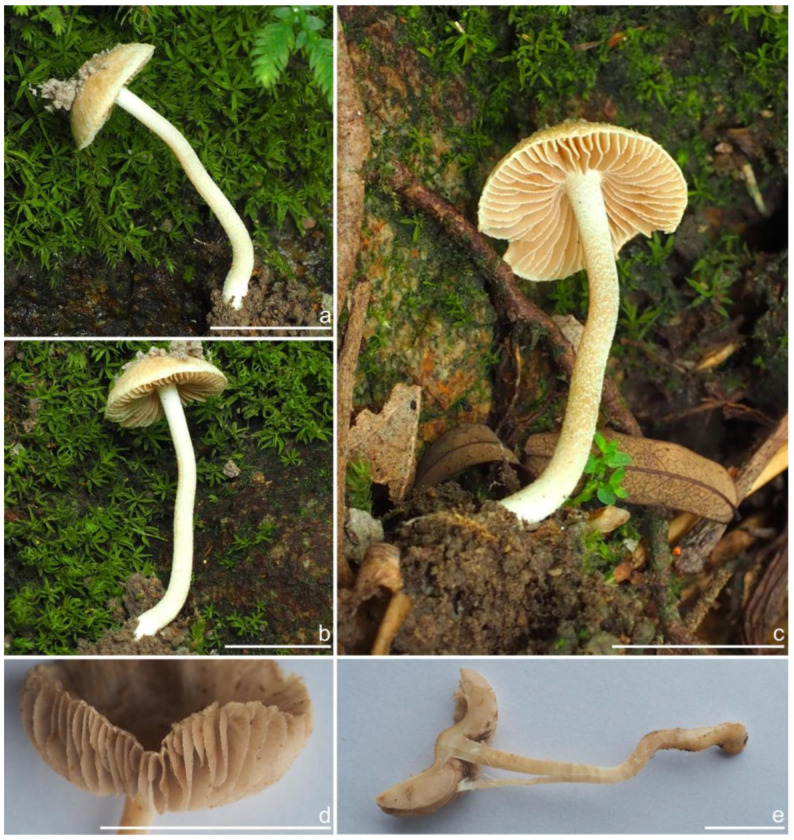
Basidiomata of *Inocybe luxiensis*. (**a**–**c**) NJ3961 (FCAS3834); (**d**,**e**) FYG2857 (FCAS3833, holotype); Scale bars: 10 mm. Photos by (**a**–**c**) Y.-P. Ge; (**d**,**e**) Y.-G. Fan.

**Figure 17:**
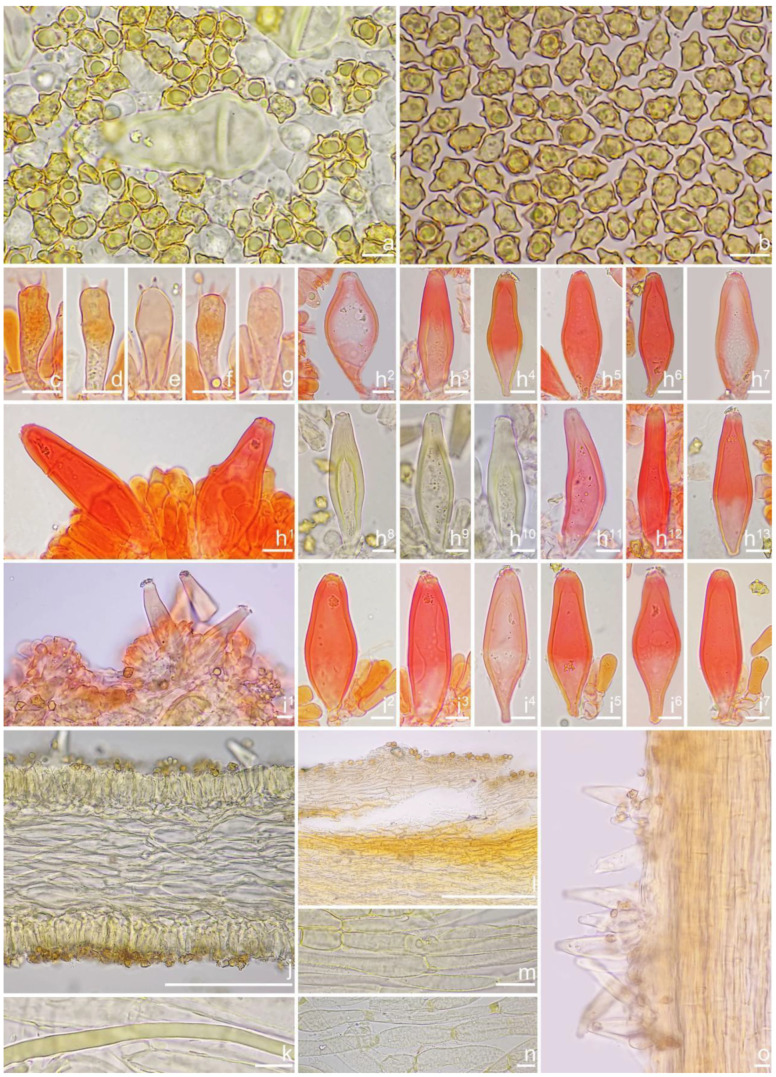
Microscopic features of *Inocybe luxiensis* (FCAS3833, holotype). (**a**,**b**) Basidiospores. (**c**–**g**) Basidia. (**h^1^**–**h^13^**) Pleurocystidia. (**i^1^**–**i^7^**) Cheilocystidia. (**j**) Cross-section of lamellae. (**k**) Oleiferous hyphae. (**l**) Pileipellis. (**m**) Pileipellis upper hyphae. (**n**) Pileipellis lower hyphae. (**o**) Stipitipellis with caulocystidia. Scale bars: (**a**–**i^7^**,**k**,**m**–**o**) 10 μm; (**j**,**l**) 100 μm. Photos by X. Chen.

***Inocybe luxiensis*** Y.G. Fan, Y.P. Ge & X. Chen sp. nov. Figure 16 and Figure 17

**Chinese name**. 潞西丝盖伞

**MycoBank**. MB 855826

**Etymology**. Luxi is the old name of Mang City (Yunnan Province), in reference to its type locality.

**Diagnosis**. *Inocybe luxiensis* has small basidiomata, cream-white to earthy yellow pileus, subcrowded lamellae, entirely pruinose stipe, angular–nodulose basidiospores, and thick-walled hymenial cystidia. Most similar to *I. ailaoensis*, but differs from it by the less crowded lamellae, more prominent nodules in basidiospores, thinner walled hymenial cystidia usually tapering into small pedicel, and typically encrusted hyphae in the lower layer of pileipellis.

**Holotype**. China, Yunnan Province, Dehong Dai-Jingpo Autonomous Prefecture, Mang City, Santai Mountain, in mixed forests dominated by fagaceous trees, 24°20′12″ N, 98°23′56″ E, 10 July 2018, Y.G. Fan, FYG2857 (FCAS3833), GenBank accession number: ITS (PP217733); LSU (PP230771) and RPB2 (PP238450).

*Basidiomata* small-sized. *Pileus* 10–18 mm in diameter, hemispherical when young, then convex to plano-convex, applanate to uplifted with a small umbo when overmature, margin depressed to straight; surface dry, wooly fibrillose when young, fibrillose to rimulose, crenulate toward the margin; pale yellowish-white (2A4) to dirty yellowish (3A5), color deepened around the center or when overmature. *Lamellae* 2–3 mm deep, moderately crowded, adnexed, initially whitish (1A1) to yellowish-white (1A4), becoming yellowish, brownish (4B4) to brownish-yellow (5B3) when old, often with unequal lamella, edge fimbriate or slightly serrate to wavy when mature. *Stipe* 22–31 × 1.5–2 mm, central, solid, terete, often slightly swollen at the apex and base, pruinose throughout the stipe surface; white (1A1) at the stipe apex, yellowish (3B6) downward, longitudinally striate. *Context* solid, fleshy in pileus, 0.2 mm thick at mid-radius, up to 3 mm thick under the umbo, uniformly yellowish (2B4) to pale yellow–brownish (3B3), fibrillose and striate in the stipe, concolorous with the pileus context. *Odor* spermatic.

*Basidiospores* [100/3/3] (6.1) 6.2–7.9–8.9 (10.0) × (4.1) 4.8–5.5–6.6 (7.0) μm, Q = (1.01) 1.13–1.42–1.71 (1.80), Q_m_ ± SD = 1.42 ± 0.17, angular with 8–15 small protruding nodules, subconical at apices, apiculus indistinct, pale yellow to yellow, at times with one or more intracellular circular to oblong oil inclusions. *Basidia* 17–26 × 6–9 μm, mostly claviform, broadly claviform, hyaline, mostly 4-sterigmate, sterigmata 3–5 μm in length. *Pleurocystidia* 40–51–66 × 11–15–27 μm (*n* = 40), abundant, mostly fusiform to lageniform, at times utriform or broadly claviform, crystallization present at the apices, base tapered into small pedicel, at times nearly rounded to subtruncate, thick-walled, walls pale yellowish-green, up to 2–3 (5) μm thick. *Cheilocystidia* 45–55–68 × 15–17–25 μm (*n* = 40), resemble pleurocystidia, fusiform, broadly fusiform, or lageniform, thick-walled, walls up to 3 μm wide. *Cheiloparacystidia* 14–19 × 5–7 μm, clavate to oblong, hyaline, smooth, mixed with numerous metuloidal cheilocystidia. *Caulocystidia* 40–51–76 × 13–15–21 μm (*n* = 20), mostly fusiform, lageniform or broadly fusiform, at times narrowly lageniform, abundant, thick-walled, walls up to 3 μm thick, colorless. *Cauloparacystidia* 10–23 × 6–11 μm, oblong, ovoid or clavate, thin-walled, colorless, mixed with caulocystidia. *Hymenophoral trama* 68–120 μm thick, subregularly arranged, colorless, composed of thin-walled, colorless, smooth, mostly inflated and cylindrical hyphae, 3–19 μm wide. *Pileipellis* a cutis of two layers, 100–160 μm wide, upper layer interwoven, pale yellow to brownish, hyphae cylindrical, encrusted, thin-walled, hyaline 3–6 μm wide; lower layer regularly arranged, inflated hyphae, yellow to yellowish-brown, encrusted, hyphae 5–17 μm wide. *Pileal trama* regularly arranged, composed of inflated hyphae up to 27 μm in diameter, colorless, smooth, thin-walled. *Stipitipellis* a cutis composed of yellowish, cylindrical, thin-walled hyphae 4–12 μm wide, smooth. *Oleiferous hyphae* 2–6 µm wide, yellowish, smooth, mostly present in stipe and pileal trama, at times in hymenophoral trama. *Clamp connections* present and common in all tissues.

**Habitat and Phenology**. From late June to August, in broad-leaved forests dominated by fagaceous trees in subtropical to warm temperate China, also occurs in tropical montane *Castanopsis*-dominated forests in Thailand and on the Korean peninsula.

**Distribution**. China (Yunnan, Zhejiang, Shandong, Sichuan), Thailand (Chiang Mai), South Korea.

**Additional specimens examined**. China, Zhejiang Province: Lishui City, Songyang County, under fagaceous forests, 16 October 2022, Y.P. Ge & Q. Na, NJ3961 (FCAS3834); Shandong Province: Qingdao City, Laoshan District, Lao Mountain, under broad-leaved forest, 15 August 2023, G.H. Liu, 20230815-9 (FCAS4022).

**Remarks**. *Inocybe luxiensis* is found in Yunnan and Zhejiang Provinces in fagaceous forests mixed with coniferous trees in subtropical China. It is characterized by the single habit, small basidiomata, tan to yellowish-white pileus, moderately crowded lamellae, entirely pruinose stipe with longitudinal stripes, angular–nodulose basidiospores, and thick-walled hymenial cystidia. *Inocybe angustifolia* is similar to *I. luxiensis* in having white basidiomata, entirely pruinose stipes, nodulose basidiospores, and thick-walled hymenial cystidia, but the former has larger and slender basidiomata, conspicuously more crowded lamellae, and more prominent nodules in basidiospores [52,54]. *Inocybe luxiensis* is phylogenetically close to *I. ailaoensis* and *I. lutosa*. However, *I. ailaoensis* has a grayish-white pileus, shorter hymenial cystidia usually with a blunt or subtruncate base, and less prominent nodules in basidiospores; *I. lutosa* has more earthy yellow pileus and less pronounced nodules in basidiospores.

According to the LSU phylogeny generated by Horak et al. (2015) [54], The Thai material DED8043 appeared to be a sister to the core group of *I. angustifolia*, which was placed in the lineage of *I. luxiensis* in our phylogeny. The color photograph of DED8043 has white basidiomata with moderately crowded lamellae which agrees well with Chinese collections. Three additional sequences from the environmental sample or root tips strongly suggest the occurrence of this fungus in evergreen broad-leaved forest in Sichuan and Zhejiang Provinces and a sea sand habit in South Korea.

***Inocybe olivaceonigra*** (Horak, E.) Garrido, Biblthca Mycol. 1988, *120*, 177

***Astrosporina olivaceonigra*** Horak, E. Persoonia 1979, *10*, 194

**Figure 18:**
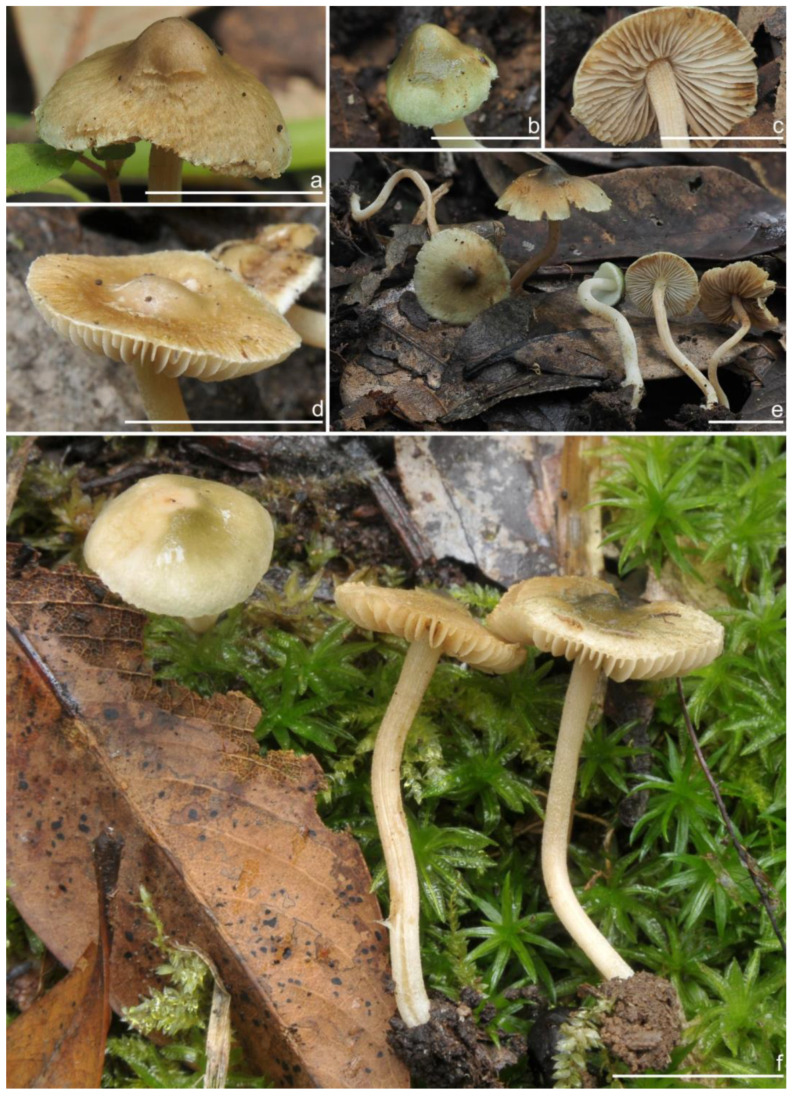
Basidiomata of *Inocybe olivaceonigra*. (**a**,**c**) FYG10675 (FCAS4068), (**b**,**e**) FYG10673 (FCAS4067), (**d**,**f**) FYG2015350 (FCAS3850); Scale bars: (**a**–**f**) 10 mm. Photos by Y.-G. Fan.

**Figure 19:**
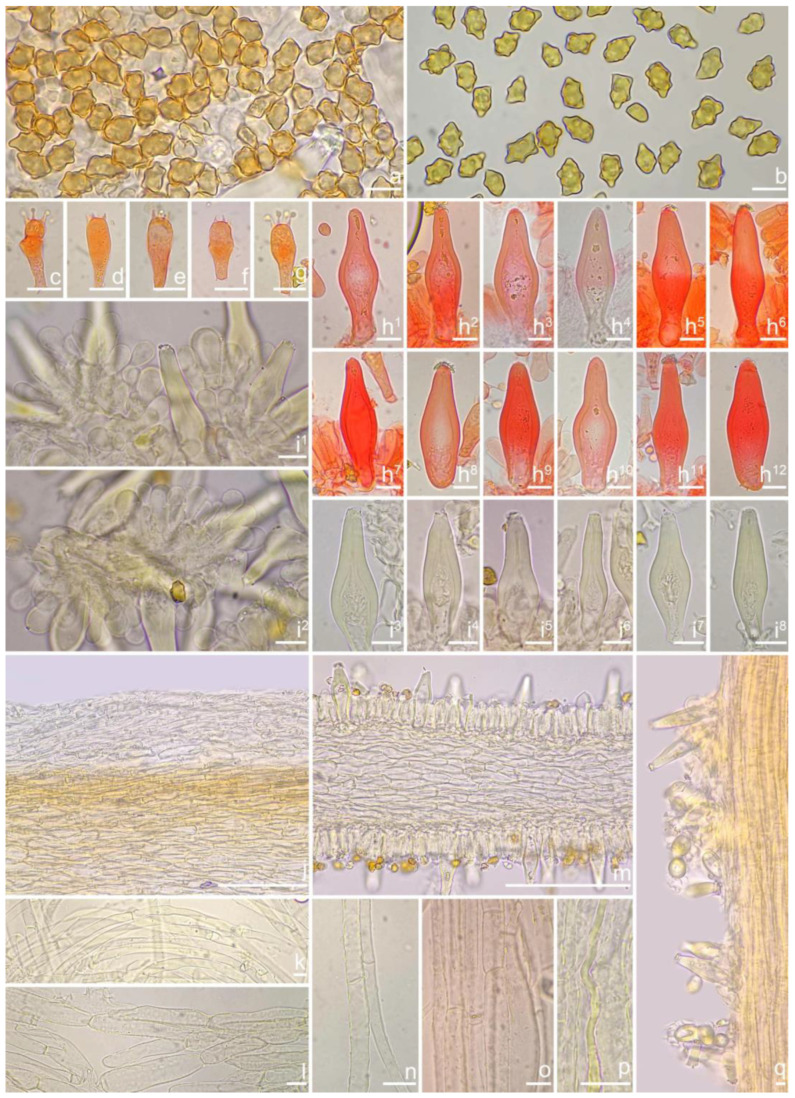
Microscopic features of *Inocybe olivaceonigra* (FCAS3850). (**a**,**b**) Basidiospores. (**c**–**g**) Basidia. (**h^1^**–**h^12^**) Pleurocystidia. (**i^1^**–**i^8^**) Cheilocystidia and cheiloparacystidia. (**j**) Pileipellis. (**k**) Pileipellis upper layer hyphae. (**l**) Pileipellis lower layer hyphae. (**m**) Cross-section of lamellae. (**n**) Stipitipellis hyphae. (**o**) Stipe trama hyphae. (**p**) Oleiferous hyphae. (**q**) Caulocystidia on stipe surface. Scale bars: 10 μm (**a**–**i^8^**,**k**–**l**,**n**–**q**), 100 μm (**j**,**m**). Photos by X. Chen.

Figure 18 and Figure 19

*Basidiomata* small-sized. *Pileus* 11–23 mm in diameter, conical when young, then convex to applanate with a blunt umbo, margin initially depressed, expanded when mature; surface dry, mostly silky smooth, rarely scaly, usually rimulose or uplifted in age; entirely dark green (27A7) at first, then fading to olivaceous (28B7) with fuliginous tinge (4C5) toward center, ochraceous (1B6) outward, the olivaceous (28B7) color stays around the center or completely disappears after rain. *Lamellae* 2.5–4.5 mm broad, subcrowded, adnexed, often with unequal lamellae, edge finely crenulate; cream white (2A1) when young, then grayish-white (1B3) and discoloring to brown (4B1) with whitish (1A1) edge in age. *Stipe* 22–50 × 1.5–3 mm, central, solid, terete, equal with a swollen base, longitudinally pruinose over the entire length; pale cream color (2A1), whitish (1A1) at the apex and base. *Context* fleshy in pileus, 0.2 mm thick at mid-radius, up to 3 mm thick under the umbo, white (1A1), greenish (27A2) near the pileipellis fibrillose and striate in the stipe, white (1A1), pinkish (5A2) near the surface. *Odor* spermatic or indistinct.

*Basidiospores* [100/3/3] (7.1) 7.2–8.5–9.8 (10.5) × (4.2) 5.0–5.8–6.3 (7.0) μm, Q = (1.23) 1.31–1.49–1.82 (1.91), Q_m_ ± SD = 1.49 ± 0.12, angular with 7–12 small nodules, yellow to yellowish-brown, apiculus indistinct, subconical at apex, at times with intracellular circular to oblong oil inclusions. *Basidia* 17–26 × 6–9 μm, mostly claviform, broadly claviform or ventricose, hyaline, mostly 4-sterigmate, but 2-sterigmate basidia present, sterigmata 3–5 μm in length. *Pleurocystidia* 41–52–66 × 13–16–26 μm (*n* = 40), abundant, mostly fusiform to lageniform, at times broadly fusiform, crystallization present at the apices, lower part tapering but usually has a swollen, blunt, rounded to nearly truncate base, thick-walled, walls up to 4 μm thick, yellowish. *Cheilocystidia* 37–50–59 × 11–17–21 μm (*n* = 40), resemble pleurocystidia, fusiform, broadly fusiform, or lageniform, thick-walled, walls up to 4 μm thick, yellowish. *Cheiloparacystidia* 14–20 × 5–10 μm, clavate to oblong, hyaline, smooth, mixed with numerous metuloidal cheilocystidia. *Caulocystidia* 33–49–62 × 10–15–20 μm (*n* = 35), abundant, descending to stipe base, mostly fusiform, lageniform or broadly fusiform, at times narrowly lageniform, thick-walled, walls up to 3 μm thick, colorless. *Cauloparacystidia* 10–23 × 6–11 μm, oblong, ovoid or clavate, thin-walled, walls yellowish, mixed with caulocystidia. *Hymenophoral trama* 68–120 μm thick, subregularly arranged, colorless, composed of thin-walled, colorless, smooth, mostly inflated and cylindrical hyphae, 3–19 μm wide. *Pileipellis* divided into two layers, 100–155 μm wide, upper layer interwoven, colorless, gelatinized, hyphae cylindrical, smooth, thin-walled, hyaline 3–7 μm wide; lower layer regularly arranged, pale yellow to yellow, smooth to encrusted, slightly inflated hyphae 7–13 μm wide. *Pileal trama* regularly arranged, composed of inflated hyphae up to 23 μm in diameter, pale yellowish, smooth, thin-walled. *Stipitipellis* a cutis composed of pale yellow, cylindrical, thin-walled hyphae 3–9 μm wide, smooth. *Oleiferous hyphae* 1.5–4 µm wide, yellowish, diverticulate, mostly present in stipe and pileal trama, at times in hymenophoral trama. *Clamp connections* present and common in all tissues.

**Habitat**. Scattered or in small groups in *Castanopsis* forests.

**Distribution**. China (Yunnan).

**Additional specimens examined**. China, Yunnan Province: Pu’er City, Jingdong Yi Autonomous County, ecological station, in *Fagaceae* forests, 24°32′36″ N, 101°1′29″ E, 20 September 2015, Y.G. Fan, FYG2015350 (FCAS3850), FYG2015351 (FCAS3851); Kunming City, Qiongzhu temple, alt. 2100 m, under *Castanopsis*, 26 July 2011, T. Bau & Y.G. Fan 2,011,131 (HMJAU 24616); 26 July 2011, X. Jin 2011131a (HMJAU 24617); 26 July 2011, Q.X. Guo 2011131b (HMJAU 24618); 26 July 2011, S.S. Yang 2011131c (HMJAU 24619); 26 July 2011 T. Bau 2011131d (HMJAU 24620), 21 September 2006, Y.C. Li 717 (HKAS 51154); 1 August 2024, Y.G. Fan & W.J. Yu FYG10673 (FCAS4067), FYG10675 (FCAS4068).

**Remarks**. *Inocybe olivaceonigra* was described from Papua New Guinea in 1979 and subsequently found in the Yunnan Province of China in 2013 [52,53]. It occurs in *Castanopsis* forests. This olive-green-colored species can be easily recognized in the field. The Chinese material agreed well with the type specimen of this species [53]. In 2015 and 2024, we obtained four additional specimens of *I. olivaceonigra* in Yunnan Province. During microscopic observation, we observed more hymenial cystidia with a tapering but swollen bases. A more detailed description and color plates of basidiomata and microcharacters were provided in this paper. Phylogenetically, *I. olivaceonigra* is a sister to the north temperate species *I. umbratica*.

**Figure 20:**
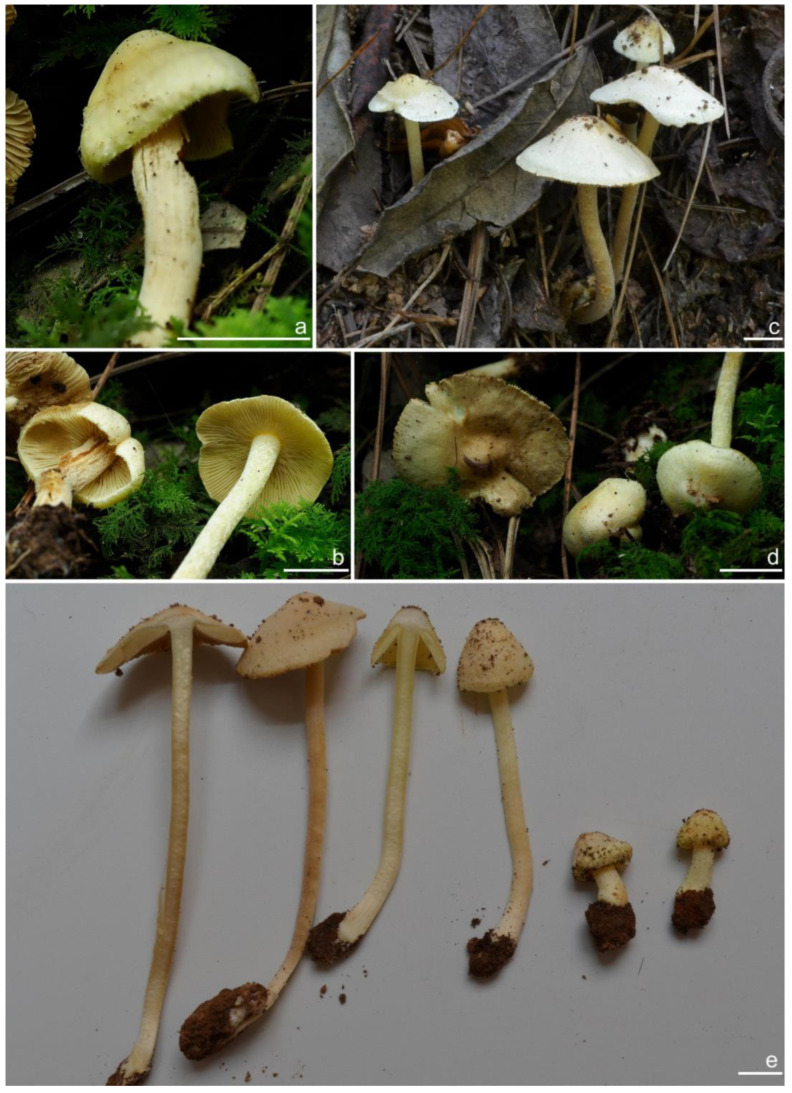
Basidiomata of *Inocybe paludinelloides*. (**a**,**b**,**d**) FYG10551 (FCAS4066). (**c**,**e**) YGF2011143 (HMJAU25956, holotype). Scale bars: 10 mm (**a**–**e**). Photos by Y.-G. Fan.

**Figure 21:**
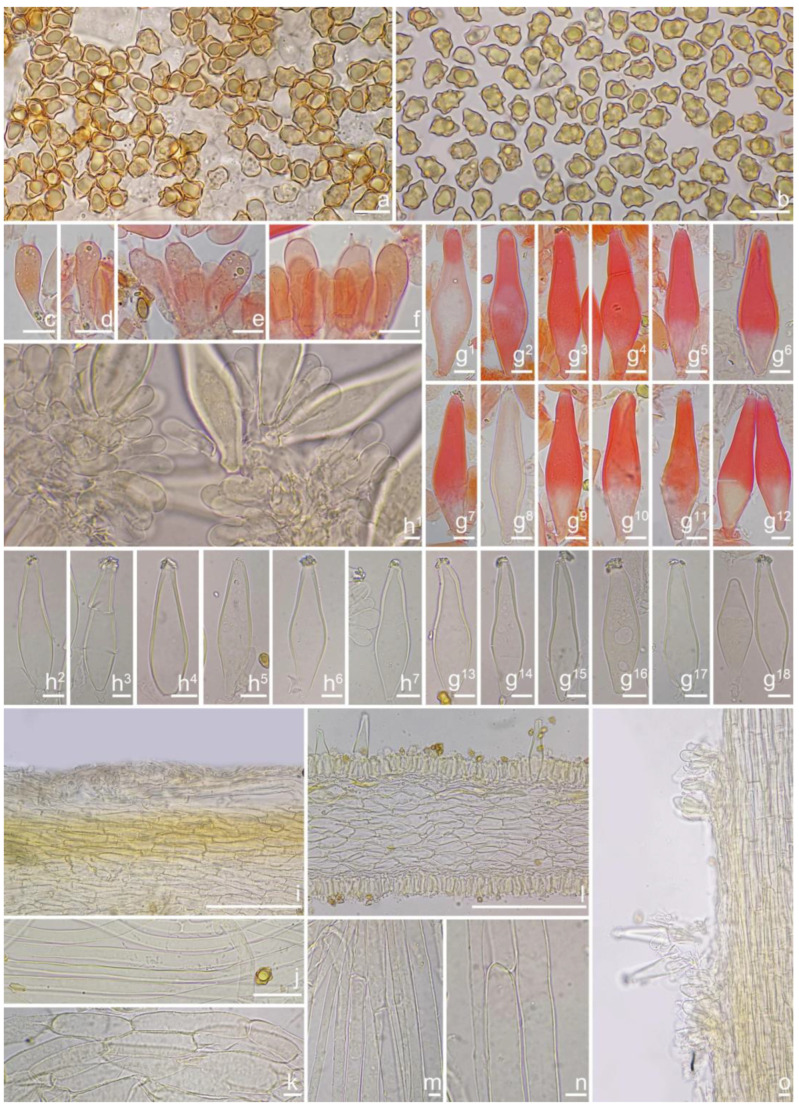
Microscopic features of *Inocybe paludinelloides* (HMJAU25956, holotype). (**a**,**b**) Basidiospores. (**c**–**f**) Basidia. (**g^1^**–**g^1^^8^**) Pleurocystidia. (**h^1^**–**h^7^**) Cheilocystidia and cheiloparacystidia. (**i**) Pileipellis. (**j**) Pileipellis upper layer hyphae. (**k**) Pileipellis lower layer hyphae. (**l**) Cross-section of lamellae. (**m**) Stipitipellis hyphae. (**n**) Stipe trama hyphae. (**o**) Stipitipellis with caulocystidia and cauloparacystidia. Scale bars: (**a**–**h^7^**,**j**–**o**) 10 μm, (**i**,**l**) 100 μm. Photos by X. Chen.

***Inocybe paludinelloides*** Bau, T.; Fan, Y.G., sp. nov. Figure 20 and Figure 21

**Chinese name**. 微黄丝盖伞

**MycoBank**. MB 855828

**Etymology**. In reference to its similarity with *I. paludinella.*

**Diagnosis**. The new species is characterized by small basidiomata with bright yellow to pale yellow or dirty white colors, becoming orange to reddish after bruising, non-marginate bulbose stipe base, angular–nodulose basidiospores, and bright yellow walls of the cystidia. Most similar to *I. spectabilis*, but differs from it by the smaller basidiospores, shorter hymenial cystidia, and its ecology usually under *Castanopsis* and *Pinus*.

**Holotype**. China, Yunnan Province: Kunming City, Yeya Lake, in mixed forests dominated by *Castanopsis* and *Pinus*, 25°08′56″ N, 102°52′01″ E, 2190 m, 27 July 2011, Tolgor Bau & Yu-Guang Fan YGF2011143 (HMJAU 25956), GenBank accession number: ITS (MG938541); LSU (MG825002).

*Basidiomata* small-sized. *Pileus* 18–32 mm, conical when young, expanding to paraboloid to convex or plano-convex to applanate with a small umbo, margin initially inrolled, then depressed and becoming straight; surface dry, glabrous with hairy margin, then smooth-fibrillose to fibrillose-rimulose, fibrillose, distinctly so toward pileus margin; bright yellow (1A6) when young, then discoloring to pale yellow (2A3) when mature or dirty whitish-yellow (4A2) in overmature individuals, cortina absent. *Lamellae* up to 1–2 mm deep, crowded, adnexed, unequal in length, alternately distributed with 2–3 tiers of lamellulae, pale yellow (2A2) with a greenish (27A2) tinge at first, becoming yellowish (2B4) or brown (5B4), edges paler, fimbriate. *Stipe* 55–75 × 2.5–5.0 mm, solid, terete, occasionally flexuous equal with a swollen base, densely pruinose along the entire length, beige (1B1), pale to bright yellow (4A3), base covered by yellowish-tinged (1A2) tomentose hyphae, staining orange (5A3) to reddish (8A2) after bruising. *Context* white (1A1), pale to bright yellow (2A3), becoming orange (5A3) after cutting, fleshy, 1.5–2.5 mm thick under the pileus center; context fibrillose in stipe, beige (1B1), pale yellow (2A3) to bright yellow (2A6), paler toward base, striate. *Odor* acidulous.

*Basidiospores* [*n* = 100/3/3] (6) 6.4–**7.8**–9.2 (9.5) × (4.5) 4.5–5.5–6.9 (7) μm, Q = (1.14) 1.18–1.42–1.70 (1.78), Q_m_ ± SD = 1.42 ± 0.15, with 8–16 small nodules about an angular outline, apiculus indistinct, at times with one intracellular circular to oblong oil inclusions; pale yellow to yellowish-brown. ***Basidia*** 26–32 × 7–12 μm, mostly claviform to broadly claviform, hyaline, mostly 4-sterigmate, but 2-sterigmate basidia occasionally observed, sterigmata 2–5 μm in length. *Pleurocystidia* 50–69–87 × 16–19–23 (*n* = 40) μm, abundant, mostly fusiform, at times broadly lageniform, apices obtuse or rounded, crystallization present at the apices, base usually tapered into small pedicel, at times obtuse and non-pedicellate toward the base, thick-walled, walls up to 2 μm thick, colorless to pale yellow. *Cheilocystidia* 50–65–86 × 11–17–26 μm (*n* = 40), abundant, resemble pleurocystidia, mostly fusiform, and broadly fusiform, occasionally sublageniform, thick-walled, walls yellowish. *Cheiloparacystidia* 12–20 × 5–12 μm, broadly clavate, hyaline, colorless, thin-walled, smooth, mixed with numerous metuloidal cheilocystidia. *Caulocystidia* 48–65–80 × 12–**17**–22 μm (*n* = 20), mostly fusiform or lageniform, abundant, descending the entire length of the stipe, thick-walled, walls up to 3 μm thick, pale yellowish. *Cauloparacystidia* 15–25 × 7–13 μm, clavate, thin-walled, colorless, mixed with caulocystidia. *Hymenophoral trama* 75–113 μm thick, regularly arranged, composed of colorless, thin-walled, colorless, smooth, slightly inflated hyphae 6–25 μm wide, cylindrical hyphae occasionally observed. *Pileipellis* a cutis divided into two layers, 75–138 μm thick, upper layer interwoven, colorless to pale yellowish, hyphae cylindrical, smooth, thin-walled, 3–6 μm wide; lower layer regularly arranged, yellow to yellowish-brown in color, smooth, hyphae 5–17 μm wide. *Pileal trama* subregularly arranged, colorless, hyphae 3–26 μm wide, smooth, thin-walled, inflated. *Stipitipellis* regularly arranged, colorless or pale yellowish in color, composed of cylindrical hyphae, 7–26 μm wide, thin-walled, smooth. *Oleiferous hyphae* 3–8 µm wide, yellowish, diverticulate, mostly present in the pileal and the stipe trama. *Clamp connections* present and common in all tissues.

**Habitat**. In evergreen broad-leaved forests dominated by fagaceous trees mixed with *Ericaceae* or *Pinus*.

**Distribution**. China (Yunnan).

**Additional specimens examined**. China, Yunnan Province, Kunming City, Yeya Lake, in mixed forests dominated by *Castanopsis* and *Pinus*, 25°08′56″ N, 102°52′01″ E, 26 July 2024, Yu-Guang Fan FYG10551 (FCAS4066); Qujing City, Shizong County, Niusu Village, al. 1820 m, in ever-green broad-leaved forest dominated by fagaceous trees mixed with *Ericaceae* or *Pinus,* 21 July 2020, Li-Ping Tang (MHKMU Tang-3013, duplicated FCAS3847); Diqing Tibetan Autonomous Prefecture, Shangri-La City, Haba Snow Mountain Yi Village, under ever-green broad-leaved forest dominated by fagaceous trees mixed with *Ericaceae* or *Pinus*, 27°23′4″ N, 100°6′22″ E, alt. 3214 m, 25 August 2020, Hong-Yan Huang (MHKMU T. Huang-559, duplicated, FCAS3846).

**Remarks**. *Inocybe paludinelloides* is an uncommon yellow species occurring in rich soil under evergreen broad-leaved forests in southwestern China. The color of this species changes to brick-red when bruised or becoming yellowish-white when overmature. *Inocybe subangustifolia* shares yellowish basidiomata, but has shorter stipes, shorter hymenial cystidia, an ecology under *Eucalyptus* spp. or *Acacia* spp., and a southern hemisphere distribution [51]. *Inocybe paludinella* has whitish to straw-colored pileus, shorter stipe, smaller basidiospores with less pronounced knobs at apex, mostly fusiform to lageniform hymenial cystidia without an elongated neck, and an ecology with *Salix*, *Alnus* or spruce [37,45].

*Inocybe paludinelloides* is phylogenetically a sister to *I. spectabilis*. Indeed, these two species shares highly similar macromorphologies. However, *I. paludinelloides* has smaller basidiospores with a less pronounced knob at apex, sublageniform to narrowly fusiform hymenial cystidia, narrower cheiloparacystidia, an ecology under *Castanopsis* and *Pinus*, and a geographic distribution in southwestern China. Sequence comparison between the two species reveal 0.91% differences (5/556) in ITS, 0.15% differences (2/1323) in LSU, and 1.03% differences (7/681) in *rpb2.*

**Figure 22:**
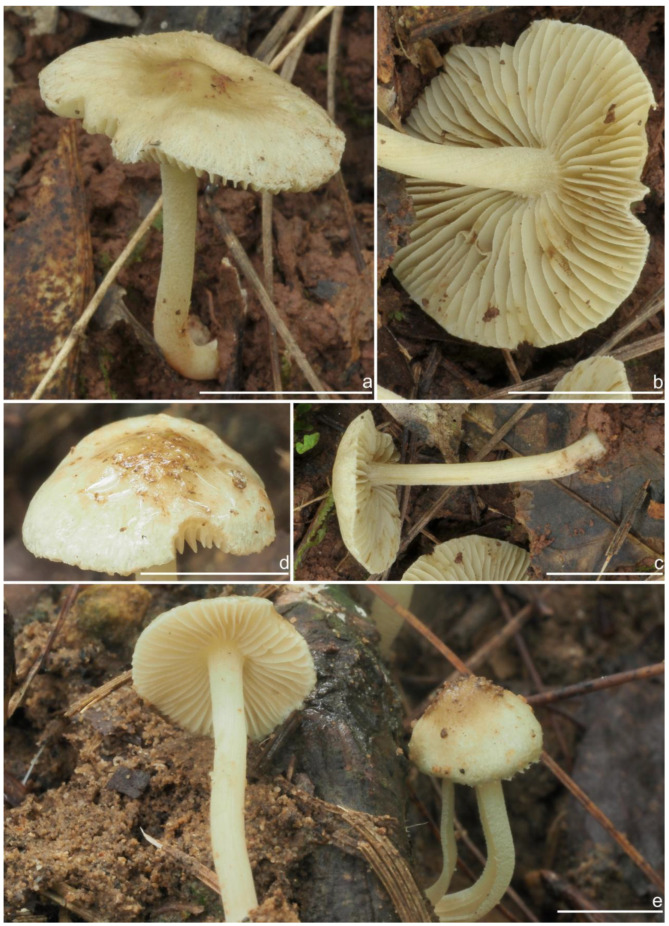
Basidiomata of *Inocybe simaoensis*. (**a**–**c**) FYG2015395 (FCAS3835, holotype); (**d**,**e**) FYG2858 (FCAS3836). Scale bars: 10 mm. Photos by Y.-G. Fan.

**Figure 23:**
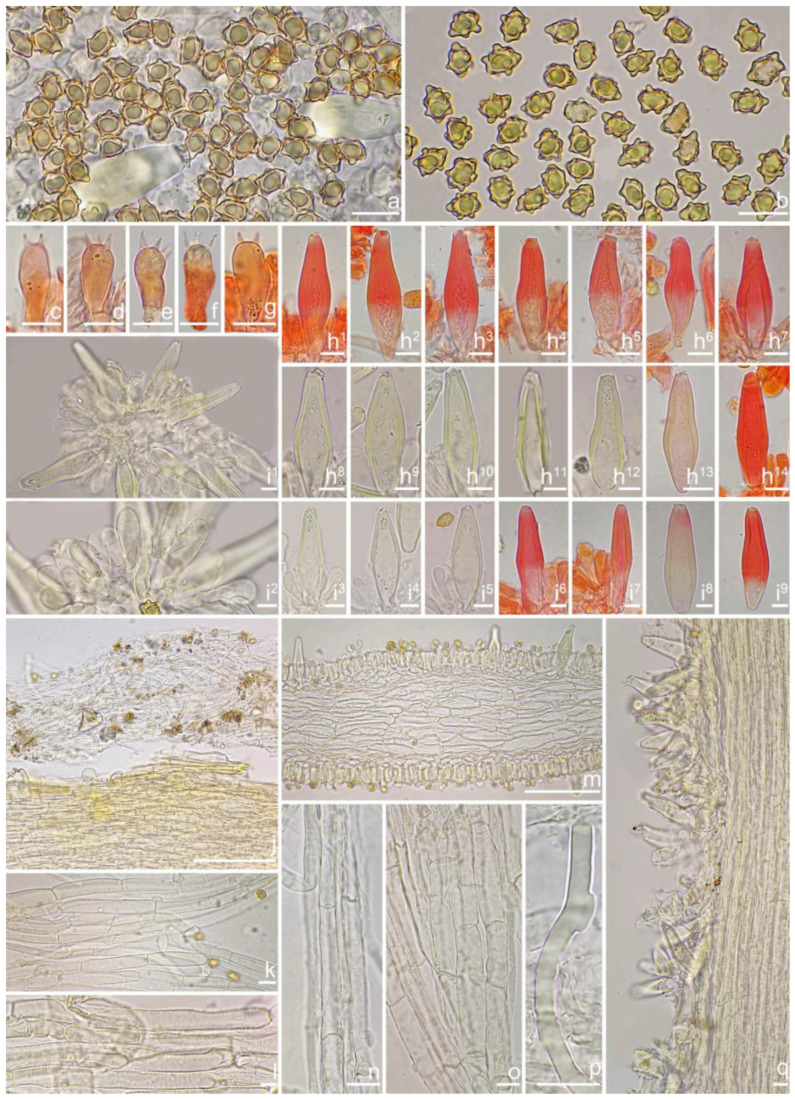
Microscopic features of *Inocybe simaoensis* (FCAS3835, holotype). (**a**,**b**) Basidiospores. (**c**–**g**) Basidia. (**h^1^**–**h^14^**) Pleurocystidia. (**i^1^**–**i^9^**) Cheilocystidia and cheiloparacystidia. (**j**) Pileipellis. (**k**) Pileipellis upper layer hyphae. (**l**) Pileipellis lower layer hyphae. (**m**) Cross-section of lamellae. (**n**) Stipitipellis hyphae. (**o**) Stipe trama hyphae. (**p**) Oleiferous hyphae. (**q**) Stipitipellis with numerous caulocystidia. Scale bars: (**a**–**i^9^**,**k**–**l**,**n**–**q**) 10 μm; (**j**,**m**) 100 μm. Photos by X. Chen.

***Inocybe simaoensis*** Y.G. Fan, W.J. Yu & X. Chen, sp. nov. Figure 22 and Figure 23.

**Chinese name**. 思茅丝盖伞.

**MycoBank**. MB 855830.

**Etymology**. Simao is the old name of Pu’er City (Yunnan Province), in reference to its type locality.

**Diagnosis**. *Inocybe simaoensis* has small white basidiomata, glabrous pileus with woolly margin, moderately crowded lamellae, entirely pruinose stipe, angular–nodulose basidiospores, and thick-walled hymenial cystidia. Most similar to *I. keteleeriicola*, but differ from it by the fuliginosus-tinged pileus center, moderately crowded lamellae, enlarged stipe base, more prominent nodules in basidiospores, thicker walls of hymenial cystidia, ecology in fagaceous forests, and different phylogenetic placement.

**Holotype**. China, Yunnan Province, Pu’er City, Roadside 323 highway, in fagaceous forests, 22°49′47″ N, 100°58′37″ E, 23 September, Y.G. Fan, 2015, FYG2015395 (FCAS3835), GenBank accession number: ITS (PP217734).

***Basidiomata*** small-sized. ***Pileus*** 15–18 mm in diameter, hemispherical when young, then campanulate to plano-convex with indistinct umbo when mature, margin crenulate with indistinct hairs when young, fugacious; surface dry or viscid when moist, nearly glabrous toward the center, but finely subtomentose near the margin when young, then wooly–fibrillose to fibrillose–rimulose; ivory (1A2) to grayish-white (1A3) with pale fuliginosus (4C5) tinge around the center. ***Lamellae*** 2–3 mm deep, adnexed, moderately crowded, alternately distributed with 3–4 layers of lamellulae, initially whitish (1B2), becoming grayish to yellowish when mature, edges entire to finely crenulate but not fimbriate. ***Stipe*** 27–35 × 2–2.8 mm, central, solid, terete, base slightly enlarged but not bulbous, white (1A1) pruinose along the entire length, longitudinally striate; surface white (1A1), ivory white (23A1) to beige (1B1). ***Context*** fleshy in the pileus, 0.5 mm thick at mid-radius, up to 3 mm thick under the umbo, cream white (2A1) to very pale cream yellow (2A2) in the pileus, fibrillose and striate in stipe cream white (2A1). ***Odor*** aromatic and sweet.

***Basidiospores*** [*n* = 100/2/2] (5.2) 6.9–**7.7**–8.8 (9.2) × (4.8) 5.0–**5.6**–6.5 (7.1) μm, Q = (1.02) 1.15–**1.36**–1.61 (1.78), Q_m_ ± SD = 1.37 ± 0.15, ellipsoid with 9–15 small protruding nodules, apiculus indistinct, with a prominent subconical knob at the apex, pale yellowish to yellow, with one intracellular circular to oblong oil inclusion. ***Basidia*** 18–27 × 7–9 μm, mostly claviform to broadly claviform, hyaline, mostly 4-sterigmate, but 2-sterigmate basidia occasionally observed, sterigmata 2–4 μm in length. ***Pleurocystidia*** 41–**52**–65 × 12–**13**–18 μm (*n* = 40), mostly fusiform, lageniform, at times ventricose, crystallization present at the apices, thick-walled, walls up to 4 μm wide, pale yellow to pale yellow–green, extremely abundant. ***Cheilocystidia*** 40–**48**–63 × 11–**14**–22 μm (*n* = 40), resemble pleurocystidia, mostly fusiform to lageniform, occasionally (sub)utriform, lower part tapering and tending to be pedicellate, occasionally with obtuse to sessile bases. ***Cheiloparacystidia*** 12–23 × 6–12 μm, clavate to ovoid, hyaline, thin-walled, smooth, mixed with numerous metuloidal cheilocystidia. ***Caulocystidia*** 36–**59**–68 × 9–**12**–14 μm (*n* = 20), mostly fusiform or lageniform, abundant, distributed through the entire length of the stipe, thick-walled, walls up to 3 μm thick, colorless, crystallization present at the apices. ***Cauloparacystidia*** 12–19 × 8–17 μm, pyriform, ovoid or clavate, thin-walled to slight thin- to thick-walled, walls up to 1 μm thick, colorless, mixed with caulocystidia. ***Hymenophoral trama*** 83–175 μm thick, regularly arranged, colorless to yellowish in color, composed of thin-walled, colorless, smooth, mostly inflated and cylindrical hyphae, 4–13 μm wide. ***Pileipellis*** divided into two layers, 230–380 μm wide, upper layer interwoven, colorless, hyphae cylindrical, more or less gelatinized, smooth, thin-walled, hyaline 3–10 μm wide; lower layer regularly arranged, yellowish in color, hyphae 5–19 μm wide, hyaline, nearly colorless, slightly inflated, encrusted. ***Pileal trama*** arranged of regularly hyphae up to 22 μm wide, inflated, colorless, smooth, thin-walled. ***Stipitipellis*** a cutis composed of yellowish, cylindrical hyphae, thin-walled, 2–10 μm wide. ***Oleiferous*** hyphae 2–6 µm wide, pale yellowish or nearly colorless, diverticulate, mostly present in stipe trama. ***Clamp connections*** present and common in all tissues, but more common in pileipellis and stipitipellis.

**Habitat**. In the fagaceous forest, around September in China.

**Distribution**. China (Yunnan), Thailand (Chiang Mai).

**Additional specimens examined**. China, Yunnan Province, Dehong Dai-Jingpo Autonomous Prefecture, Mang City, Santai Mountain, in fagaceous forests, 10 July 2018, Y.G. Fan, FYG2858 (FCAS3836).

**Remarks**. *Inocybe simaoensis* has small white basidiomata, glabrous pileus with woolly margin when young. There is always a fuliginosus tinge toward the disk and the lamellae are only moderately crowded or subcrowded. According to our phylogeny, three specimens (DED8147, DED8050, DED8161), which were identified as “*Inocybe* sp. DED8147” [54] are grouped together with the two Chinese materials of *I. simaoensis,* indicating a distribution of this species in northwestern Thailand. The Thai material DED8050 has a more pronounced umbo in the pileus whose margin is becoming uplifted when overmature [54]. The hymenial cystidia of *I. simaoensis* vary in the wall thickness and transparency, with relatively young stage specimens tending to have more translucent and thinner walls (Figure 23(h^8^–h^10^,i^3^–i^5^)), while fully or overmature specimens have more thickened, yellow walls (Figure 23(h^1^–h^7^,i^6^–i^9^)). These conspicuously thickened-walled cystidia of *I. simaoensis* are reminiscent of the cylindrical hymenial cystidia of *I. senkawaensis*, but differ in smaller size measuring 32–52 μm in length and broader basidiospores with only inconspicuous nodules [19]. The young specimens of *Inocybe simaoensis* is superficially similar to *I. keteleeriicola*, but differs from it by the fuliginosus-tinged pileus center, more prominent nodules in basidiospores, thicker walls of hymenial cystidia, and occurs in fagaceous forests. Phylogenetically, *I. simaoensis* is a sister to *I. danxiaensis*, but the latter has dull yellowish basidiomata, less pronounced nodules in basidiospores, thinner walled hymenial cystidia, and a sweet smell.

**Figure 24:**
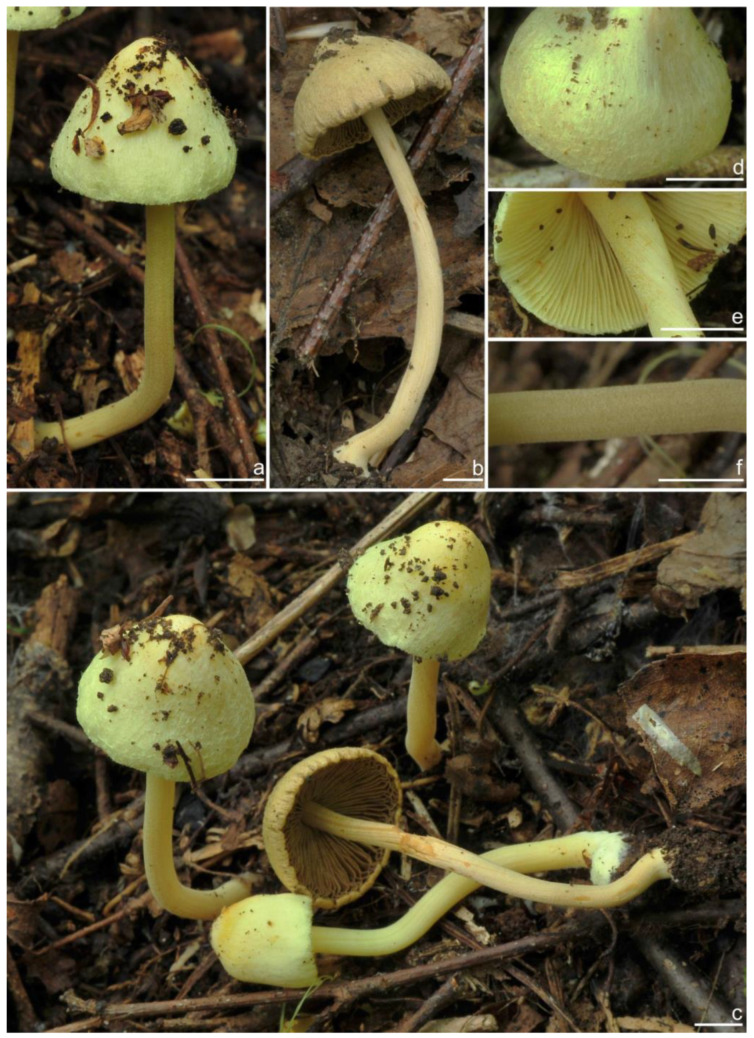
Basidiomata of *Inocybe spectabilis*. (**a**–**f**) FYG2342 (FCAS3844*,* holotype). Scale bars: 10 mm. Photos by Y.-G. Fan.

**Figure 25:**
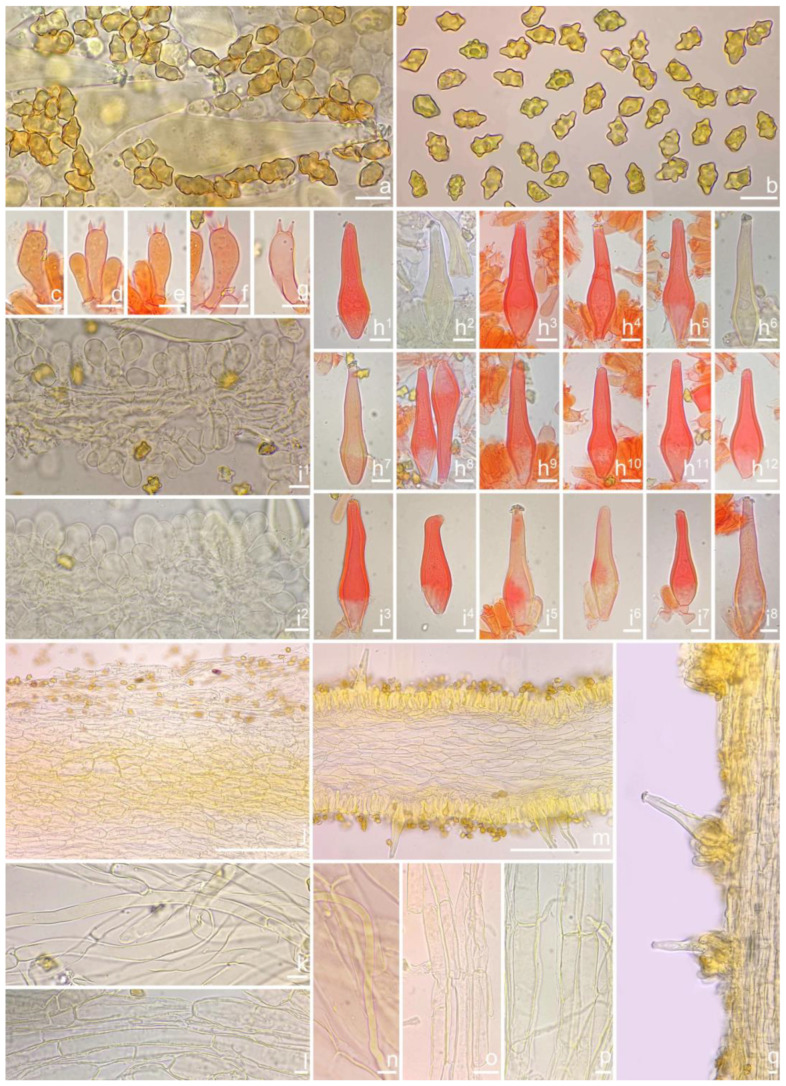
Microscopic features of *Inocybe spectabilis* (FCAS3844, holotype). (**a**,**b**) Basidiospores. (**c**–**g**) Basidia (**h^1^**–**h^12^**) Pleurocystidia. (**i^1^**–**i^8^**). Cheilocystidia and cheiloparacystidia. (**j**) Pileipellis. (**k**) Pileipellis upper layer hyphae. (**l**) Pileipellis lower layer hyphae. (**m**) Cross-section of lamellae. (**n**) Oleiferous hyphae. (**o**) Stipitipellis hyphae. (**p**) Stipe trama hyphae. (**q**) Stipitipellis. Scale bars: (**a**–**i^8^**,**k**,**n**–**q**) 10 μm; (**j**,**m**) 100 μm. Photos by X. Chen.

***Inocybe spectabilis*** Y.G. Fan, L.W. Qin & B. Wang, sp. nov. Figure 24 and Figure 25

**Chinese name**. 黄美丝盖伞

**MycoBank**. MB 855832

**Etymology**. In reference to its bright-yellow colored basidiomata.

**Diagnosis**. *Inocybe spectabilis* has bright yellow to dirty yellow basidiomata, subtomentose pileus, crowded to rather crowded lamellae, slender and entirely pruinose stipes, angular–nodulose basidiospores with a prominent knob at the apex, and abundant lageniform hymenial cystidia. Most similar to *I. paludinelloides* but differs from it by the larger basidiospores, longer hymenial cystidia with apparently elongated necks, and its association with *Betula*.

**Holotype**. China, Jilin Province, Yanbian Korean Autonomous Prefecture, Antu county, Erdaobaihe Town, Changbai Mountain Nature Reserve, under a Birch forest, 41°41′49″ N, 127°42′55″ E, 10 August 2017, Y.G. Fan, FYG2342 (FCAS3844), GenBank accession number: ITS (PP217738); LSU (PP230779) and RPB2 (PP238455).

*Basidiomata* small, slender. *Pileus* 11–20 mm in diameter, hemispherical, subconical to subcylindrical convex at first, then campanulate, convex when mature, non-umbonate, margin decurved to straight; surface dry, initially glabrous toward the center and subtomentose outward, silky smooth to fibrillose-rimulose when mature; dirty yellow (3B4), pale bright yellow (1A7) to yellowish-white (1A3). *Lamellae* 1–1.5 mm deep, adnexed, rather crowded, unequal in length, alternately distributed with 3–4 tiers of lamellulae, greenish-yellow (29A3) to pale bright yellowish (1A5) at first, brownish-yellow (5B3) to brown (4D4) upon maturity; edge finely crenulated. *Stipe* 55–75 × 2–2.5 mm, terete, equal, solid, entirely pruinose, longitudinally striate, at times twisted, slightly swollen at base, base covers with yellow tomentose hyphae, pale bright yellow (2A5) to yellowish-white (2A3). *Context* fleshy in pileus, yellowish (1A2) to oily yellow (3A3), 2–3 mm thick under the umbo, 0.3–0.5 mm thick at mid-radius; fibrillose and striate in stipe, yellowish (1A2) to oily yellow (3A3). *Odor* grassy.

*Basidiospores* [*n* = 100/3/3] (7.1) 7.5–9.1–11.5 (15.8) × (4.5) 4.9–**5**.7–7.2 (8.1) μm, Q = (1.25) 1.37–1.59–1.86 (2.55), Q_m_ ± SD = 1.59 ± 0.16, angular with 10–13 small nodules, with a prominent subconical knob at apices, apiculus indistinct; yellowish to brownish-yellow, at times with one intracellular circular to oblong oil inclusions. *Basidia* 19–30 × 7–10 μm, mostly claviform to narrowly claviform, thin-walled, mostly 4-sterigmate, at times with 2-sterigmate, sterigmata 2–4 μm in length, at times yellow tinge inclusions. *Pleurocystidia* 59–69–86 × 14–16–18 μm (*n* = 40), mostly lageniform to broadly lageniform, with an elongated neck, but occasionally fusiform, crystallization present at the apices, lower part tapering with a subtruncate base, thick-walled, walls yellowish, up to 2 μm thick at the middle and thicker near the apex (up to 3 μm). *Cheilocystidia* 53–67–80 × 15–17–22 μm (*n* = 40), resemble pleurocystidia, mostly lageniform with a long neck usually flexuous. *Cheiloparacystidia* 10–15 × 8–13 μm, rather abundant, pyriform, ovoid, hyaline, colorless, thin-walled, mixed with cheilocystidia. *Caulocystidia* 40–63–82 × 15–20–29 μm (*n* = 20), lageniform, or broadly lageniform, thick-walled, apices crystalliferous, descending to stipe base. *Cauloparacystidia* 14–20 × 6–10 μm, pyriform, ovoid, broadly clavate, or oblong, thin-walled, colorless, mixed with caulocystidia. *Hymenophoral trama* 72–120 μm thick, regularly arranged, colorless, hyphae, thin-walled, colorless, smooth, hyphae inflated 5–17 μm wide, cylindrical hyphae occasionally observed. *Pileipellis* divided into two layers, 50–120 μm wide, upper layer interwoven, colorless, hyphae cylindrical, smooth, thin-walled, hyaline 3–8 μm wide; lower layer regularly arranged, yellowish to greenish-yellow, smooth, hyphae 9–20 μm wide. *Pileal trama* 450–800 μm thick, subregularly arranged, colorless, hyphae 7–20 μm wide, smooth, thin-walled, inflated. *Stipitipellis* regularly arranged, pale yellowish to brownish, composed of cylindrical hyphae, 4–12 μm wide, thin-walled, smooth. *Oleiferous hyphae* 3–5 µm wide, yellowish, smooth, mostly present in the stipe trama. *Clamp connections* present and common in all tissues.

**Habitat**. On rich soil in mixed forests dominated by *Betula* and *Acer*.

**Additional specimens examined**. China, Jilin Province, Tonghua City, Baijiyao Forest Park, in mixed forests dominated by *Betula* and *Acer*, 18 August 2015, Y.G. Fan, L.W. Qin & B. Wang, FYG2015067 (FCAS3845), FYG2015067a (FCAS4015).

**Remarks**. *Inocybe spectabilis* is an uncommon species found in mixed forests dominated by *Betula* and *Acer*. The new species is characterized by small bright yellow basidiomata, fibrillose-tomentose pileus, slender and pruinose stipe, angular–nodulose basidiospores, and thick-walled hymenial cystidia usually having a long neck. In the *I. umbratica* group, *I. paludinella* and *I. subangustifolia* also exhibit a yellowish tint throughout the fruiting bodies. However, *I. paludinella* has whitish to straw-colored pileus, shorter stipe, smaller basidiospores with less pronounced knobs at apex, mostly fusiform to lageniform hymenial cystidia without an elongated neck, and an ecology with *Salix*, *Alnus* or spruce [37,45]; *I. subangustifolia* has shorter stipes, smaller basidiospores, fusiform to sublageniform or subcylindrical hymenial cystidia, an ecology under *Eucalyptus* spp. or *Acaccia* spp., and a southern geographical distribution [51]. *Inocybe spectabilis* is phylogenetically a sister to *I. paludinelloides*. Indeed, these two species share highly similar macromorphologies. However, *I. spectabilis* has larger basidiospores with a more pronounced knob at the apex, lageniform hymenial cystidia with a conspicuous elongated neck and an obtuse base, pyriform cheiloparacystidia, ecology under *Betula*, and a geographical distribution in northeastern China. Sequence comparison between the two species revealed 0.91% differences (5/556) in ITS, 0.15% differences (2/1323) in LSU, and 1.03% differences (7/681) in *rpb2.*

***Inocybe umbratica*** Quél., Assoc. Franç. Avancem. Sci., Congr. Rouen 1883, *12*, 500, 1884

=*Inocybe suaveolens* Stuntz, D.E. Mycologia 1950, *42*, 110.

**Figure 26:**
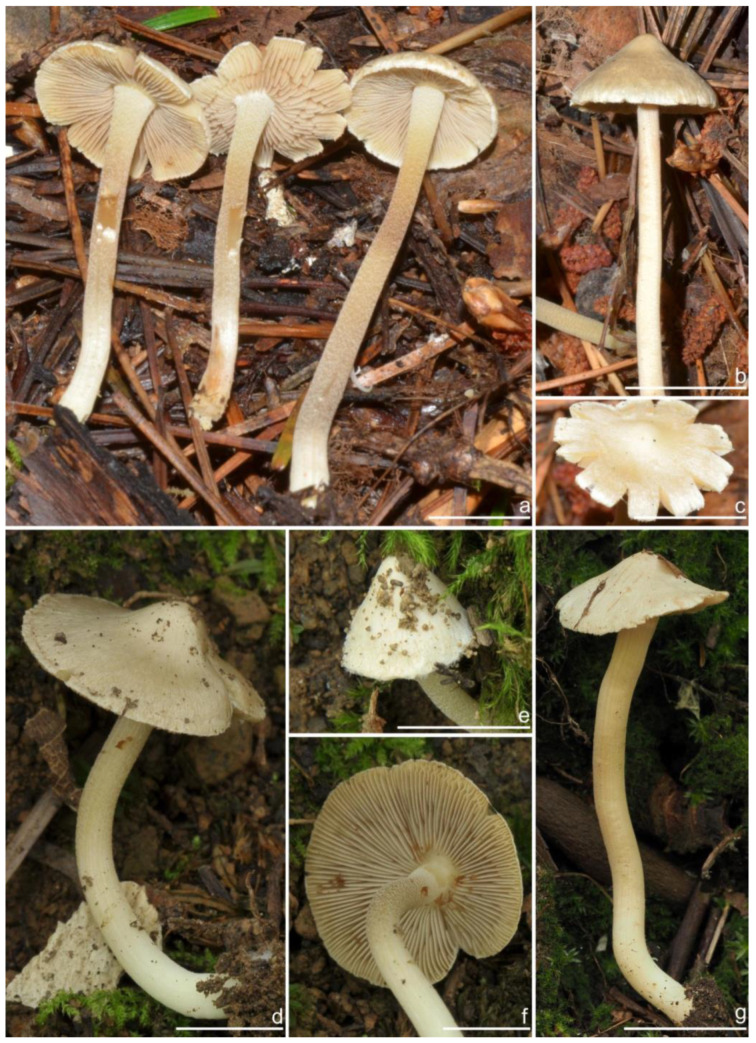
Basidiomata of *Inocybe umbratica*. (**a**,**c**) fan3753 (FCAS3840); (**b**) FYG3995 (FCAS3842); (**d**–**f**) FYG2015065 (FCAS4061); (**g**) FYG3780 (FCAS3839). Scale bars: 10 mm (**a**–**g**). Photos by Y.-G. Fan.

**Figure 27:**
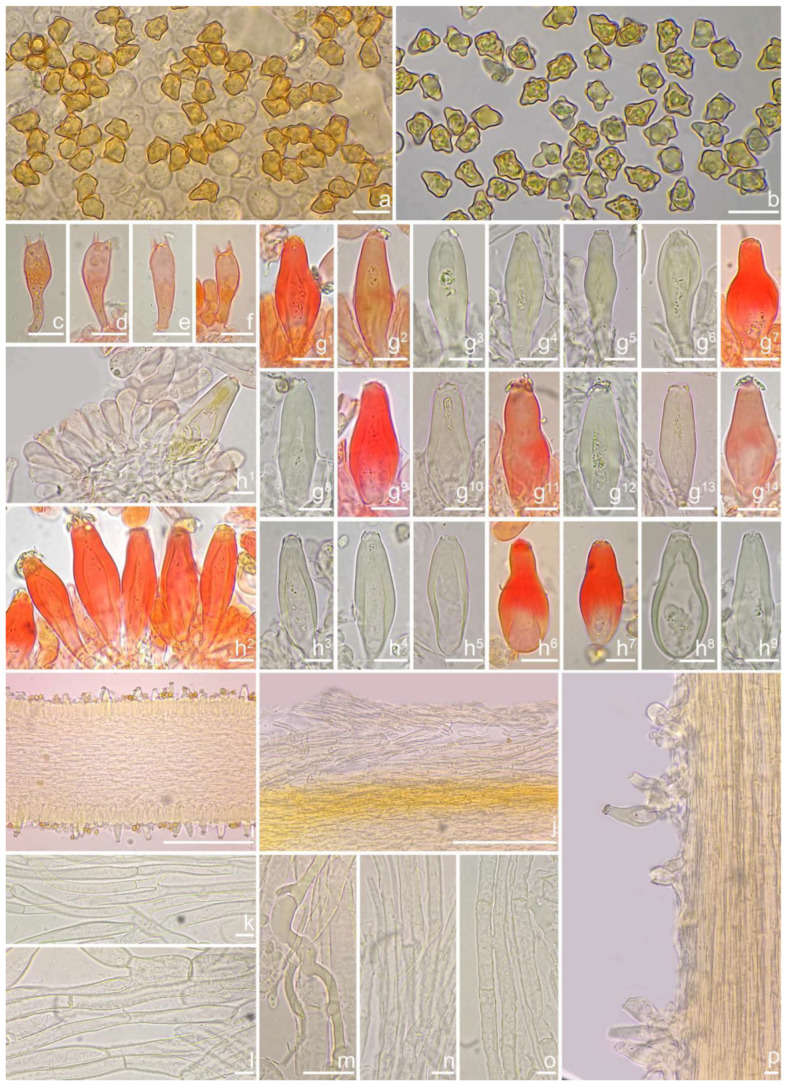
Microscopic features of *Inocybe umbratica* (FCAS3839). (**a**,**b**) Basidiospores. (**c**–**f**) Basidia. (**g^1^**–**g^14^**) Pleurocystidia. (**h^1^**–**h^9^**) Cheilocystidia. (**i**) Cross-section of lamellae. (**j**) Pileipellis. (**k**) Pileipellis upper layer hyphae. (**l**) Pileipellis lower layer hyphae. (**m**) Oleiferous hyphae. (**n**) Stipitipellis hyphae. (**o**) Stipe trama hyphae. (**p**) Stipitipellis with caulocystidia. Scale bars: (**a**–**h^9^**,**k**–**p**) 10 μm; (**i**,**j**) 100 μm. Photos by X. Chen.

**Figure 28:**
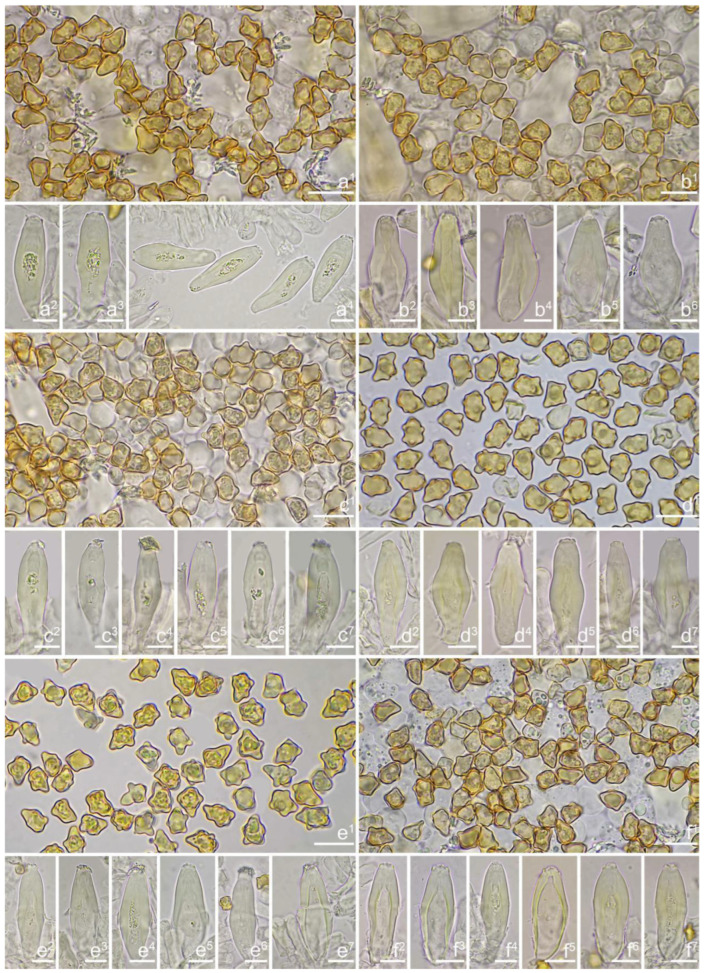
Basidiospores and hymenial cystidia of *Inocybe umbratica*. (**a^1^**–**a^4^**) FYG251 (FCAS3841); (**b^1^**–**b^6^**) fan3753 (FCAS3840); (**c^1^**–**c^7^**) FYG3995 (FCAS3842); (**d^1^**–**d^7^**) FYG4034 (FCAS3843); (**e^1^**–**e^7^**) FYG3780 (FCAS3839); (**f^1^**–**f^7^**) FYG8538 (FCAS4023). Scale bars: 10 μm. Photos by X. Chen.

Figure 26, Figure 27 and Figure 28

*Basidiomata* small-sized. *Pileus* 12–40 mm in diameter, conic at first, then campanulate to convex or applanate with a small umbo, margin incurved at first, depressed to straight upon maturity, finely longer than the lamellae; surface dry, initially glabrous, then silky smooth, at times fibrillose to appressed fibrillose to scaly with small squamules, split at the margin with age; white (1A1), ivory white (23A1), off-white (1B1), grayish-white (1C1), pale yellowish (242) when overmature. *Lamellae* 1.0–2.5 mm deep, adnexed, rather crowded, unequal in length, alternately distributed with 2–3 tiers of lamellulae, white (1A1) at first, becoming grayish-white (1C1), yellowish-white (1A2) to brownish (5B2) with age, edge finely crenulated. *Stipe* 23–76 × 1.2–5 mm, terete, equal with a slightly swollen but non-marginate base, solid, densely pruinose along the entire length; ivory white (23A1) at the apex or base, ivory white (23A1) to pale yellowish (3A4) at the middle. *Context* fleshy in pileus, white (1A1), 1.0–1.5 mm thick under the umbo, 0.3–0.7 mm thick at mid-radius; striate and shiny in stipe, white (1A1), fibrillose. *Odor* spermatic or earthy.

*Basidiospores* [100/6/6] (6.1) 6.5–7.5–8.5 (11) × (4.2) 4.7–5.5–6.1 (7.0) μm, Q = (1.12) 1.21–**1.41**–1.73 (1.83), Q_m_ ± SD = 1.41 ± 0.14, angular with 5–8 nodules, apiculus indistinct, pale yellowish to yellowish-brown, apices subconical, with intracellular circular to oblong oil inclusions. *Basidia* 17–29 × 7–10 μm, mostly claviform to broadly claviform, hyaline, generally 4-spored, seldom also 2-spored. *Pleurocystidia* 30–38–48 × 11–13–16 μm (*n* = 40), mostly fusiform to narrowly fusiform, occasionally broadly fusiform, apex usually crystallization, at the apices obtuse, lower part tapering with an obtuse or nearly rounded to subtruncate base, thick-walled, walls up to 5 μm thick, colorless to pale yellowish, extremely abundant. *Cheilocystidia* 30–35–46 × 12–15–19 μm (*n* = 40), resemble pleurocystidia, mostly fusiform, occasionally (sub)broadly claviform. *Cheiloparacystidia* 13–20 × 5–7 μm, clavate to ovoid, or oblong, hyaline, smooth, mixed with cheilocystidia. *Caulocystidia* 31–44–50 × 11–15–18 μm (*n* = 20), mostly fusiform to broadly fusiform, descending to stipe base, thick-walled, walls up to 3 μm thick, colorless, crystallization present at the apices. *Cauloparacystidia* 10–17 × 6–12 μm, pyriform, ovoid, clavate, or oblong, thin-walled, colorless, mixed with caulocystidia. *Hymenophoral trama* 70–130 μm thick, regularly arranged, colorless, composed of thin-walled, colorless, smooth, mostly cylindrical hyphae, 3–7 μm wide. *Pileipellis* divided into two layers, 100–155 μm wide, upper layer interwoven, colorless, hyphae cylindrical, rough, thin-walled, hyaline 3–7 μm wide; lower layer regularly arranged, pale yellowish to yellow–greenish, rough, hyphae 7–13 μm wide. *Pileal trama* regularly arranged, hyphae up to 15 μm in diameter, inflated, colorless, smooth, thin-walled. *Stipitipellis* a cutis composed of pale yellowish, cylindrical hyphae, thin-walled, 5–18 μm wide. *Oleiferous hyphae* 2–7 µm wide, colorless, diverticulate, mostly present in stipe trama, occasionally at the pileal trama. *Clamp connections* present and common in all tissues.

**Habitat**. In coniferous forests or mixed forests.

**Distribution**. Europe, China (Jilin, Heilongjiang, Nei Mongol), Japan, North America.

**Specimens examined**. China, Jilin Province: Yanbian Korean Autonomous Prefecture, Antu County, Erdaobaihe Town, Changbai Mountain Nature Reserve, in mixed forests dominated by *Populus*, *Betula*, *Picea*, *Abies* and *Pinus*, 2 August 2019, Y.G. Fan & W.J. Yu, FYG3780 (FCAS3839), fan3753 (FCAS3840), 28 August 2013, Y.G. Fan & W.J. Yu, FYG3995 (FCAS3842), in coniferous forests, 21 July 2013, Y.G. Fan & W.J. Yu, FYG4034 (FCAS3843), 42°17′57″ N, 128°7′39″ E, 17 August 2020, FYG251 (FCAS3841); Laoyeling Mountain, in coniferous forests, 6 August 2023, B. Wang, FYG8538 (FCAS4023); Dunhua City, Huangnihe River, Tuanbei Forest Farm, in *Larix* and *Picea* forests, 22 August 2015, Y.G. Fan & L.W. Qin, 2,015,086 (FCAS4060); Tonghua City, Dongchang District, Baijifeng National Forest Park, in *Abies*, *Acer* and *Populus* forests, 18 August 2015, Y.G. Fan, L.W. Qin & B. Wang, FYG2015065 (FCAS4061); Nei Mongol Autonomous Region: Hinggan League, Arxan City, Wuchagou, Niufentai, in *Picea* forests, 3 August 2022, T.Z. Liu & Y.M. Gao, CFSZ 24948.

**Remarks**. *Inocybe umbratica* was described from Europe (France) and has also been found in North America and East Asia [19,37,40,41,42]. It is characterized by whitish to grayish-white basidiomata, silky smooth to appressed scaly pileus, crowded lamellae, entirely pruinose stipes with a small bulb, and an ecology under coniferous or mixed forests. In China, *I. umbratica* has been reported from Jilin and Heilongjiang Provinces in northeastern China [106,107]. In our phylogenetic results, eight specimens labeled “*I. suaveolens*” including the a paratype (stz4800) and one specimen (ACAD14282) labeled “*I. abundans*” clustered together with our specimens. *Inocybe abundans* was a brownish species described from New York [108], ACAD14282 was apparently mislabeled. *Inocybe suaveolens* is a whitish species described in 1950 from Mt. Rainier National Park (northwestern America) [109] and is similar to *I. umbratica*, so we consider them as synonyms.

Key to 17 species of *Inocybe* sect. *Umbraticae*
1 Basidiomata white, silver–gray, earthy yellow or becoming yellowish-white when overmature21* Basidiomata dull yellow, dull orange, greenish-yellow, bright yellow, olive green, or reddish-brown92 Lamellae crowded to rather crowded32* Lamellae moderately crowded to subcrowded73 Occurring in North America only, lamellae rather broad, basidiospores elongate-nodulose < 5 μm in width*I. alabamensis*3* Occurring in North temperate region, lamellae narrow, basidiospores angular–nodulose generally > 5 μm in width44 Associated with conifers54* Associated with deciduous trees or shrubs65 Basidiomata small sized, white, pallid, in *Keteleeria* forests, basidiospores angular with indistinct nodules, known only in subtropical China*I. keteleeriicola*5* Basidiomata medium sized, silver white, basidiospores angular with 5–8 distinct nodules, under *Picea, Larix* or mixed forests, widespread in north temperate regions*I. umbratica* (=*I. suaveolens*)6 Basidia bearing four sterigmata, hymenial cystidia wall > 3 μm76* Basidia mostly bearing two sterigmata, hymenial cystidia wall < 3 μm at the middle, nearly thin-walled toward the base*I. dabaensis*7 Basidiospores 6.1–8.1 × 4.2–5.8 μm, Q_m_ = 1.42, hymenial cystidia > 17 μm in width*I. angustifolia*7* Basidiospores 7.2–9 × 4.9–6.1 μm, Q_m_ = 1.44, hymenial cystidia generally < 17 μm in width*I. ailaoensis*8 Pileus earthy yellow, basidiospores weakly angular with indistinct nodules.*I. lutosa*8* Pileus whitish, not completely yellow, basidiospores angular with prominent small nodules109 Pileus white with earthy yellow colored disk, odor spermatic, basidiospores with an average Q value of 1.36*I. luxiensis*9* Pileus white with fuliginosus colored disk, odor aromatic or sweet, basidiospores with an average Q value of 1.42*I. simaoensis*10 Basidiomata olive green or reddish-brown1110* Basidiomata yellow color, not as above1211 Pileus olive green toward the disk, stipe entirely pruinose, subtropical to tropical Asia*I. olivaceonigra*11* Pileus reddish-brown, stipe pruinose-furfuraceous, northern temperate*I. castanea*12 Pileus surface bearing copious silver velipellis*I. argenteoluta*12* Pileus without distinct velipellis1313 Endemic to Australia*I. subangustifolia*13* Occurring in northern hemisphere1414 Occurring in North America and Europe, pileus white to straw yellow, in humid habitats, in coniferous forests or *Salix*, *Alnus**I. paludinella*14* Occurring in East Asia, pileus dull yellow to greenish-yellow when young, under broad-leaved forests or mixed forests1515 Under *Betula* in north temperate China, hymenial cystidia with strikingly elongate neck*I. spectabilis*15* Under fagaceous trees in subtropical China, hymenial cystidia not as above1616 Basidiomata staining brick-red when bruised or overmature, hymenial cystidia lageniform*I. paludinelloides*16* Basidiomata not changing color when bruised, hymenial cystidia fusiform*I. danxiaensis*

## 5. Discussion

The “*I. umbratica–paludinella* clade” was first referenced in Horak et al. (2015) and recovered five species-level lineages [54], of which only two could be named, *I. angustifolia* for supposed Thai representatives of *I. angustifolia* (a type from Papua New Guinea) and a North American species, *I. alabamensis*. This clade of five species received strong support based only on the analysis of 28S (LSU) data. Matheny and Bougher (2017) added the Australian species *I. subangustifolia* to the group and referred for comparison to a New Zealand species, *I. straminea* (E. Horak) [51]. However, the inclusion of *I. straminea* has not been confirmed with molecular phylogenetic data. *Inocybe paludinella* has been allocated to *I.* sect. *Marginatae* or *I.* sect. *Petiginosae* on the basis of the morphology [18,44]. Nevertheless, the *I. umbratica–paludinella* group is fully supported by our three-gene phylogenetic analysis (Figure 1) and is a sister to the lineage containing the reddish-brown species *I. castanea*, which has been assigned to *I.* sect. *Petiginosa* [44,45]. The *I. castanea* lineage forms a strongly supported group with the *I. umbratica–paludinella* clade, which is phylogenetically distant from the *I. petiginosa* group. Considering the polyphyletic status of the morphologically defined sectional classification of sect. *Marginatae*, a new section, *I.* sect. *Umbraticae*, is proposed to accommodate species in the *I. umbratica–paludinella* group and the *I. castanea* lineage. We treated the reddish-brown species *I. castanea* as being included in *I.* sect. *Umbraticae* because of the following considerations: (1) the entirely pruinose–furfuraceous stipe, angular–nodulose basidiospore outline, abundant hymenial cystidia with obtuse to rounded bases, and bi-layered pileipellis, which are often encountered in this section; (2) its stable phylogenetic position as a sister to the *I. umbratica–paludinella* group; (3) if not included in sect. *Umbraticae*, the *I. castanea* lineage should also be treated as a new section but with only two species. In the summarized three-gene phylogeny of the genus *Inocybe*, the section *Umbraticae* is a sister to a clade formed by the *I. pingala* group and the *I. viscata* group. However, species in the *I. pingala* group typically exhibit a brownish appressed-fibrillose to finely squamulose pileus, entirely pruinose stipes with bulbose marginate bases, and, at times, a two-layered pileipellis [73,110,111], whereas species in the *I. viscata* group typically have a yellowish-brown appressed scaly to squarrose pileus, entirely pruinose stipes, basidiospores with broadly obtuse nodules, and sessile hymenial cystidia [51,71,77]. Furthermore, the stability of this topography and support values has been shown to be variable when some species are added to or deleted from the phylogeny. Further studies are, therefore, required to clarify the relationships between *I.* sect. *Umbraticae* and the *I. pingala* group and/or the *I. viscata* group.

The species in *I.* sect. *Umbraticae* typically exhibit a small, slender habit, white to dull yellowish basidiomata, narrow and crowded to rather close lamellae, and an entirely pruinose stipe with only a swollen but non-marginate base. Microscopically, they have angular–nodulose basidiospores with a prominent knob at the apices, abundant hymenial cystidia with walls > 2.5 μm and generally obtuse to rounded bases, and a two-layered pileipellis with the upper layer composed of thin-walled and more or less gelatinized hyphae. The lower layer hyphae of pileipellis in most species of *I.* sect. *Umbraticae*, in contrast to the other species in this genus, are finely encrusted or only rough. However, in *I. lutosa* (Figure 15) and *I. ailaoensis* (Figure 3), the inner walls of the sublayer hyphae are unevenly thickened, whereas those of *I. castanea* are typically inflated and encrusted with tawny pigments. The basidiospore outline, including the number and shape of nodules, can be used to distinguish certain species. However, the size and Q-values of basidiospores are often similar in most species. Hymenial cystidia are abundant, and their walls are conspicuously thickened in most species of sect. *Umbraticae*. The bases of hymenial and caulocystidia are usually obtuse and lack a discernible taper toward a pedicel, with the exception of *I. luxiensis* (Figure 17). Wall thickness and transparency may vary in different developmental stages, as observed in *I. ailaoensis* and *I. spectabilis*. Specimens that are just maturing have more transparent and thinner walls, while those that are more mature have thicker walls that are yellowish in color. Nevertheless, it is difficult to identify these whitish species in sect. *Umbraticae*, an accurate identification requires the integration of microscopic, macroscopic, ecological, and, probably, molecular data.

Currently, 17 species have been confirmed in sect. *Umbraticae*; *I. senkawaensis* and *I. straminea* were not included because of the lack of sequence data. In China, thirteen species of *I.* sect. *Umbraticae* have been reported, and nine of them were found in Yunnan Province, which is the most species-rich region in China. Three major subclades can be identified within the sect. *Umbraticae*: the *castanea* subclade, the *angustifolia* subclade, and the *umbratica* subclade (Figure 1). The *angustifolia* subclade is a sister to the *umbratica* subclade. The species within the *angustifolia* subclade exhibit lamellae that are crowded or rather crowded, with five species distributed from subtropical China to Australia. *Inocybe angustifolia* was reported as a widespread species in Southeast Asia and Australasia [103], but this species has not been confirmed to occur in Australia [51]. *Inocybe subangustifolia* is notable for its dull yellow color and is the only confirmed southern hemisphere species in sect. *Umbraticae*. This species, or a close relative, may occur in South Korea, based on an ITS search in GenBank [51]. *Inocybe keteleeriicola* and *I. dabaensis* are both white species that occur in subtropical China under *Keteleeria* and fagaceous trees, respectively.

The *umbratica* subclade is the most species-rich group, with 12 species occurring from northern temperate to subtropical regions. However, certain species can extend into tropical regions of Southeast Asia. *Inocybe umbratica* is a common and widespread species in northern temperate regions, where it shares high similarities in microfeatures with *I. paludinella* [37]. These two species can be distinguished by the straw-yellow basidiomata of *I. paludinella*. *Inocybe olivaceonigra* is an impressive olive-green species that is phylogenetically a sister to *I. umbratica,* as demonstrated by Fan and Bau (2013) [53]. These two species, in turn, are sisters to the lineage unifying *I. danxiaensis* and *I. simaoensis*. *Inocybe argenteolutea* was initially considered as a forma of *I. grammata* when it was first discovered by Gulden and Lange (1971), with only one specimen. Vauras (1997) formally described it as a new species and provided morphological comparisons to *I. grammata* and *I. paludinella* [105]. However, *I. argenteolutea* was subsequently assigned to *I.* sect. *Petiginosae* by Jacboson (2008) [45]. This species is phylogenetically related to the North American species *I. alabamensis*, which was previously considered as a synonym of *I. paludinella* [51]. Indeed, the yellowish species *I. paludinella* is phylogenetically a sister to the lineage united by *I. argenteolutea* and *I. alabamensis*. In addition, two other closely related species are *I. spectabilis* and *I. paludinelloides*, which also have dull yellow-tinged basidiomata. The former is only found in southwestern China, while the latter occurs in northeastern China. *Inocybe ailaoensis*, *I. lutosa*, and *I. luxiensis* form a strongly supported lineage and are sisters to the remaining eight species, which also form a strongly supported lineage in this subclade. These three species exhibit white to earthy yellow basidiomata, particularly after rain or under overmature conditions.

The *castanea* subclade encompasses the northern temperate species *I. castanea* and an undescribed phylogenetic taxon that is endemic to North America. Three phylogenetic lineages were identified in *I. castanea* (Figure 6). Lineage 1 contains 29 samples that originated from central to northern Europe, the Far East of Russia, Japan, and China, with one exception: PBM1981 from New York. Lineage 3 comprises 19 samples that originated from central to northern North America, the Far East of Russia, and China. However, lineage 2 now includes seven samples that all reported from North America. It is noteworthy that two Yunnan (Southwestern China) collections, FYG2015149 and Huang-893, grouped into Lineage 1 and Lineage 3, respectively. Similarly, the twelve specimens collected from northeast China grouped into Lineage 1 or Lineage 2 but with no observable morphological (Figure 7) or ecological differences between the two lineages. Consequently, it can be postulated that cryptic speciation may be occurring in this species. Further studies are required to elucidate the genetic structure of these two sympatric phylogenetic lineages.

## Figures and Tables

**Figure 4:**
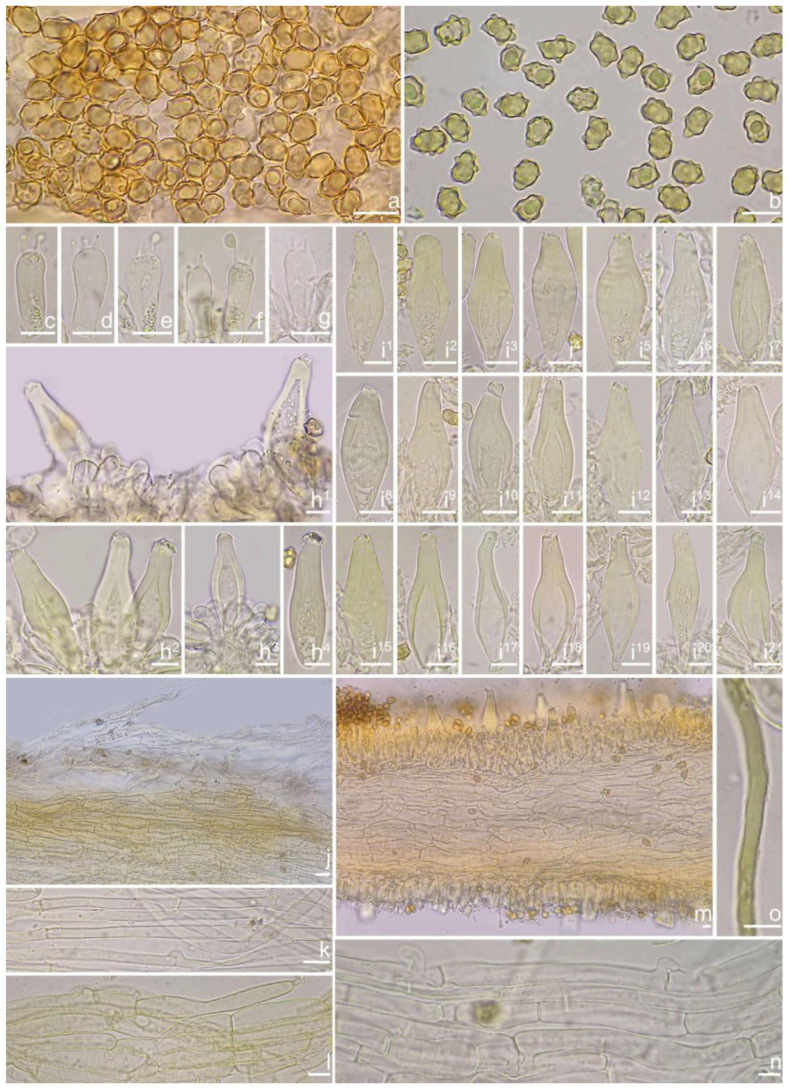
Microscopic features of *Inocybe angustifolia* (FCAS4010). (**a**,**b**) Basidiospores. (**c**–**g**) Basidia. (**h^1^**–**h^4^**) Cheilocystidia and cheiloparacystidia. (**i^1^**–**i^21^**) Pleurocystidia. (**j**) Pileipellis. (**k**) Pileipellis upper layer hyphae. (**l**) Pileipellis lower layer hyphae. (**m**) Cross-section of lamellae. (**n**) Hymenophoral trama hyphae. (**o**) Oleiferous hyphae. Scale bars: 10 μm. Photos by X. Chen.

**Figure 5:**
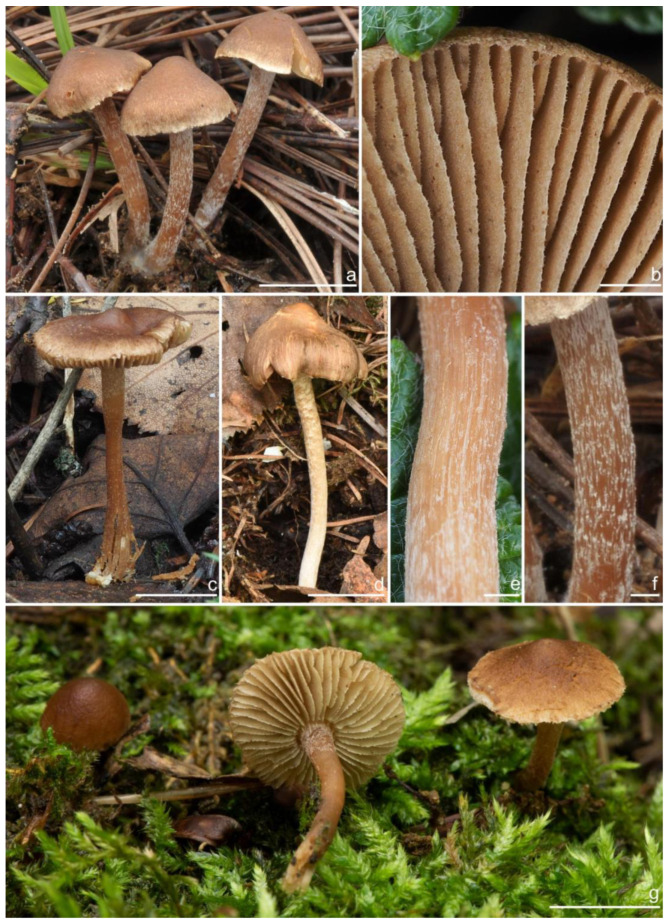
Basidiomata of *Inocybe castanea* (**a**,**f**) FYG2015149 (FCAS3852); (**b**,**e**) FYG544-2020 (FCAS3854); (**c**) FYG10-2020 (FCAS3853); (**d**) FYG1677 (FCAS3857); (**g**) FYG1803 (FCAS3856). Scale bars: (**a**–**g**) 10 mm. Photos by Y.-G. Fan.

**Table 1:** List of *Inocybaceae* taxa used in the molecular analyses.

Species	Sample No.	Locality	Habitat/Host Plants	GenBank Accession No.			References
				ITS	nLSU	RPB2	
fungal sp.	M13C4	USA	*Pinus ponderosa*	MZ017767	MZ017767		[64]
fungal sp.	M11A 6	USA	*Pinus ponderosa*	MZ017766	MZ017766		[64]
fungal sp.	ARIZ: PM093E	USA	*Pinus ponderosa*	MG761311			[65]
fungal sp.	ARIZ: PM333C	USA	*Pinus ponderosa*	MG761465			[65]
fungal sp.	C35A 9	USA	*Pinus ponderosa*	MZ017193	MZ017193		[64]
*Inocybe abdita*	STU-SMNS STU-F-0901691	Germany		NR_185439	OP164062		[66]
*I. abundans*	ACAD 14282	Canada		MG489948			Direct sub.
*I. acuta*	DB24-8-15-7	Finland		MG136902	MG136997		[67]
*I. acutata*	FYG 4322	China		OR755906			[60]
***I. ailaoensis***	**FYG2015385**	**China**	***Fagaceae* forests**	**PP217739**	**PP230780**	**PP238456**	**This study**
***I. ailaoensis***	**FYG2015386**	**China**	***Fagaceae* forests**	**PP217749**	**PP230781**	**PP238457**	**This study**
***I. ailaoensis***	**FYG2015389**	**China**	***Fagaceae* forests**	**PP993813**			**This study**
***I. ailaoensis***	**YZ20240504-S6**	**China**	**mixed forests**	**PP993797**	**PP993841**	**PQ001012**	**This study**
***I. ailaoensis***	**YZ20240504-S 18**	**China**	**mixed forests**	**PP993799**			**This study**
***I. ailaoensis***	**YZ20240504-S 19**	**China**	**mixed forests**	**PP993800**			**This study**
***I. ailaoensis***	**YZ20240504-S 22**	**China**	**mixed forests**	**PP993798**	**PP993842**	**PQ001013**	**This study**
***I. ailaoensis***	**20230801-36**	**China**	**broad-leaved forests**	**PP993823**		**PQ001021**	**This study**
***I. ailaoensis***	**HMLD 298**	**China**	**under forest land**	**PP993830**			**This study**
***I. ailaoensis***	**20230810-5**	**China**	***Fagaceae* forest**	**PP993824**			**This study**
***I. ailaoensis***	**100802-6**	**China**	**broad-leaved forests**	**PP993831**			**This study**
***I. ailaoensis***	**100807-4**	**China**	**coniferous forests**	**PP993833**			**This study**
***I. ailaoensis***	**100829-10**	**China**	**mixed forests**	**PP993822**			**This study**
***I. ailaoensis***	**120819**	**China**		**PP993825**			**This study**
***I. ailaoensis***	**20200831-96**	**China**	**mixed forests/roadside**	**PP993826**			**This study**
***I. ailaoensis***	**20210719-5**	**China**	**broad-leaved forests**	**PP993827**	**PP993854**	**PQ001022**	**This study**
***I. ailaoensis***	**20210719-11**	**China**	**broad-leaved forests**	**PP993828**	**PP993855**	**PQ001023**	**This study**
***I. ailaoensis***	**20200831-100**	**China**	**mixed forests**	**PP993829**	**PP993856**	**PQ001024**	**This study**
*I. alabamensis*	PBM 1892	USA		―	AY536280	AY536281	[46]
*I. alienospora*	PBM 3743	Australia		KP171104	KM197209	KM245970	[51]
*I. alienospora*	REH 9667	Australia		KP171105	KM197210	KM245971	[51]
*I. alloumbrina*	TENN 067007 (PBM 3775)	Australia		KJ778848	KJ801158	KM245974	[51]
*I. angustifolia*	ZT 10149	Thailand	*Castanopsis*, *Quercus*, *Lithocarpus*, and *Pinus*	GQ892990	GQ892944		[54]
*I. angustifolia*	DED 8043	Thailand	near *Castanopsis*		EU569851		[54]
*I. angustifolia*	DED 8146	Thailand	*Dipterocarpus* spp.	GQ892989	GQ892943	MH577421	[54]
*I. angustifolia*	DED 8139	Thailand	*Castanopsis*, and *Dipterocarpus*	GQ892988	GQ892942	MH577422	[54]
***I. angustifolia***	**FYG10227**	**China**	***Fagaceae* forests**	**PP993812**	**PP993850**	**PQ001017**	**This study**
*I. angustifolia*	DED 8139	Thailand		GQ892988	GQ892942	MH577422	[54]
*I. aprica*	FYG7640	China		OR755901	OR760197	OR775210	[60]
*I. arctica*	JV 2238	Norway		KY033843	KY033843		[68]
*I. argenteolutea*	EL 9906	Sweden		FN550889	FN550889		[48]
*I. argenteolutea*	21478			JF908188			[69]
*I. assimilata*	M 0020105			KM873366			[70]
*I. asterospora*	EL 100-14	Sweden		MN296110	MN296110		Direct sub.
*I. aurea*	EL 14206	Sweden		FN550877		FN550877	[48]
*I. aurescens*	FYG2015387	China		OR755913	OR760276	OR775213	[60]
*I. aurescens*	FYG2871	China		OR755902		OR775212	[60]
*I. ayangannae*	MCA 1232	Guyana			AY239018	AY337364	[47]
*I. babruka*	CAL: 1344	India		KY440086	KY549116	KY553237	[71]
*I. beninensis*	HLA 0390	Benin		MN096196	MN097888		[59]
*I. beninensis*	HLA 0467	Benin		MT994602			[59]
*I. brevisquamulosa*	ZT 10102	Thailand		NR_153123	GQ892974		[54]
*I. cacaocolor*	PBM 3790	Australia		KJ778845	KJ756464	KJ756422	[51]
*I. calida*	2000603-024			HQ604372	HQ604372		Direct sub.
*I. calida*	TAA 185175	Estonia		AM882760	AM882760		[72]
*I. calocephala*	PBM 3600	Australia			NG057234	KJ756413	[51]
*I. calospora*	JFA 12539	Sweden			AY038313	AY337365	[73]
*I. carpinicola*	FYG6307	China	*Carpinus* forests	OP207874	OP207868	OP227086	[60]
*I. castanea*	Bau 20140711	China	Coniferous forests	KU366499			Direct sub.
*I. castanea*	PBM 1981	USA			MT241842	MH577513	Direct sub.
*I. castanea*	JV 12211	Finland			MT241841	MH577512	Direct sub.
***I. castanea***	**FYG2015149**	**China**	***Pinus* forests**	**PP217767**			**This study**
***I. castanea***	**FYG10-2020**	**China**	**Coniferous forests**	**PP217750**	**PP230782**		**This study**
***I. castanea***	**FYG544-2020**	**China**	**Alpine tundra**	**PP217751**			**This study**
***I. castanea***	**GN 1412**	**China**	**mixed forests**	**PP217752**	**PP230783**	**PP238463**	**This study**
***I. castanea***	**FYG1803**	**China**	**coniferous forests**	**PP217753**	**PP230784**		**This study**
***I. castanea***	**FYG1677**	**China**	**coniferous forests**	**PP217754**	**PP230785**		**This study**
***I. castanea***	**FYG14-2020**	**China**	**coniferous forests**	**PP217755**	**PP230786**	**PP238458**	**This study**
***I. castanea***	**FYG3741**	**China**	**coniferous forests**	**PP217756**			**This study**
***I. castanea***	**GN1400**	**China**	**coniferous forest**	**PP217757**	**PP230787**	**PP238464**	**This study**
***I. castanea***	**Huang 893**	**China**	***Fagaceae* and *Pinus* forests**	**PP217758**	**PP230788**	**PP238466**	**This study**
***I. castanea***	**FYG1326**	**China**	**coniferous forests**	**PP217759**	**PP230789**		**This study**
***I. castanea***	**FYG1327**	**China**	**coniferous forests**	**PP217760**	**PP230790**	**PP238459**	**This study**
***I. castanea***	**FYG1662**	**China**	**coniferous forests**	**PP217761**	**PP230791**		**This study**
***I. castanea***	**FYG1673**	**China**	**coniferous forests**	**PP217740**	**PP230792**		**This study**
***I. castanea***	**FYG 3-2020**	**China**	**coniferous forests**	**PP217762**	**PP230793**		**This study**
***I. castanea***	**CFSZ 25181**	**China**	***Larix forests***	**PP993836**			**This study**
***I. castanea***	**CFSZ 25182**	**China**	***Larix forests***	**PP993837**			**This study**
***I. castanea***	**CFSZ 25200**	**China**	***Larix forests***	**PP993839**			**This study**
***I. castanea***	**CFSZ 25177**	**China**	***Larix forests***	**PP993835**			**This study**
***I. castanea***	**CFSZ 25187**	**China**	***Larix forests***	**PP993838**			**This study**
***I. castanea***	**FYG8683**	**China**	**coniferous forest**	**PP993817**			**This study**
***I. castanea***	**FYG8636**	**China**	**coniferous forest**	**PP993818**			**This study**
***I. castanea***	**FYG8692**	**China**	**coniferous forest**	**PP993816**			**This study**
***I. castanea***	**FYG1272**	**China**	**coniferous forest**	**PP993820**			**This study**
***I. castanea***	**FYG8672**	**China**	**coniferous forest**	**PP993819**			**This study**
*I. casuarinoides*	FYG8122	China	*Casuarina* forests	OR755899	OR759975	OR775204	[60]
*I. cerasphora*	BSI 01/184	Chile			AY380370	AY337367	[46]
*I.* cf. *ambigua*	EL 8105	Finland		FN550812	AM882796		[48]
*I.* cf. *nematoloma*	EL 128-16	Sweden		MH310768			Direct sub.
*I.* cf. *xanthomelas*	PAM 10082807	France		KP641651	KP171099		[51]
*I.* cf. *xanthomelas*	PAM 10082808	France		KP641652	KP171100		[51]
*I.* cf. *xanthomelas*	PAM 10082305	France		KP641653	KP171101		[51]
*I. chalcoceps*	TENN: 068946	Australia			NG_057228		[51]
*I. chondroderma*	PBM 1776	USA		GU949579	JN974967	MH249789	[74]
*I. conspicuospora*	PC 96042	Zambia			EU555471	EU555470	[47]
*I. curvipes*	EL 6703	Sweden		AM882813		AM882813	[72]
***I. dabaensis***	**YZ2023102844**	**China**	**fagaceous trees and *Pinus***	**PP993809**	**PP993852**	**PQ001020**	**This study**
***I. dabaensis***	**YZ2023102844** **-1**	**China**	**fagaceous trees and *Pinus***	**PP993810**	**PP993848**	**PQ001018**	**This study**
***I. dabaensis***	**YZ2023102844** **-2**	**China**	**fagaceous trees and *Pinus***	**PP993811**	**PP993849**	**PQ001019**	**This study**
***I. danxiaensis***	**FYG4389**	**China**	***Fagaceae* forest**	**PP217735**	**PP230774**		**This study**
***I. danxiaensis***	**FYG4392**	**China**	***Fagaceae* forest**	**PP217745**	**PP230775**	**PP238452**	**This study**
*I. diabolica*	EL 9006	Sweden		FN550896	FN550896		[48]
*I. dunensis*	EL 22906	France		FN550888	FN550888		[48]
*I. eburnea*	PBM 3367	Australia		KJ778851	KJ801162	KJ756424	[51]
*I. egenula*	EL 12206	Sweden		FN550884		FN550884	[48]
*I. elata*	HMJAU 37797/HMJAU 01	China		MG744559	KY773232		[75]
*I. emergens*	NLB 896	Australia			KJ729915	KJ729943	[51]
*I. epidendron*	TH 9186	Guyana		JN168725	EU569840		[47]
*I. ericetorum*	TURA 177504	Finland		NR_119994			[76]
*I. favrei*	EL 5706	Sweden		FN550886		FN550886	[48]
*I. fibrosoides*	SS 2990	Sweden		AM882827		AM882827	[74]
*I. flavipes*	MR 00383	Togo		MN096197	MN097889	MW080915	[59]
*I. flavipes*	HLA 0363	Benin		MT994601			[59]
*I. flavoalbida*	PBM 3768	Australia		KJ729873	KJ729901	KJ729932	[51]
*I. flavosquamulosa*	TBGT 10743	India		KT329450	NG_228743		[77]
*I. flavosquamulosa*	CAL 1353	India		KY440087	KY549117		[71]
*I. flavosquamulosa*	CAL 1355	India		KY440088	KY549118		[71]
*I. floccosistipitata*	CAL 1256	India		KY440089	KY549119		[71]
*I. fulvilubrica*	PBM 3352	Australia			JQ085922		[51]
*I. fuscicothurmata*	PBM 3980	North Carolina		MF487844	KY990485	MF416408	[60]
*I. fuscobrunnea*	MR 00378	Burkina Faso		MN096201	MN097893	MW219733	[59]
*I. fuscobrunnea*	HLA 0567	Ivory Coast		MT994603			[59]
*I. giacomi*	JV 21543	Finland		MK153656	MK153656		[78]
*I. glaucodisca*	PC 96081	Zambia			EU569853		[47]
*I. grammata*	EL 102B06	Sweden		FN550885		FN550885	[48]
*I. haikouensis*	FYG9868	China		OR975602	OR975620	PP366983	[51]
*I. heteromorpha*	FYG5769	China		OR755900	OR759987	OR775207	[51]
*I. hopeae*	OR 1665	Thailand			ON831503	ON553692	[79]
*I. hydrocybiformis*	CAL 1376	India		KY440090	KY549120	KY553240	[71]
*I. inodora*	EL 2405	Norway		AM882834		AM882834	[72]
*I. insulana*	CAL 1258	India		KY440092	KY549122		[71]
*I. jacobi*	2000608-010			HQ604374	HQ604374		Direct sub.
*I. jacobi*	EL 21606	France		FN550883		FN550883	[48]
*I. juji*	123	China		OR975596	OR975614	PP356982	[60]
*I. kapila*	CAL 1346	India		KY440093	KY549123		[71]
***I. keteleeriicola***	**FYG2884**	**China**	***Keteleeria* trees**	**PP217732**	**PP230769**	**PP238448**	**This study**
***I. keteleeriicola***	**FYG2918**	**China**	***Keteleeria* trees**	**PP217742**	**PP230770**	**PP238449**	**This study**
***I. keteleeriicola***	**FYG2917**	**China**	***Keteleeria* trees**	**PP993805**	**PP993846**	**PQ00101**	**This study**
*I. kurkuriya*	CAL 1352	India		KY440095	KY549125	KY553245	[71]
*I. kuruvensis*	K(M) 191734	India		KM924522	KM924517	KY553246	[71]
*I. lacera*	PBM 2541	USA		KP171144	JN974993	KM245991	[51,80]
*I. lacunarum*	JV 12244	Finland		KT958908	KT958908		[81]
*I. lanuginosa*	PBM 3023	USA		HQ232480	KP170923	KM245992	Direct sub.
*I. lasseri*	MCA 1971	Guyana			EU569857	EU569856	[47]
*I. lasseroides*	PBM 3749	Australia		KP171145	KP170924	KM245993	[51]
*I. lasseroides*	PBM 3750	Australia		KP171146	KP170925		[51]
*I. leptophylla*	BK 7-Sept-97-19 (UTC)				AY038320		[73]
*I. leptospermi*	PBM 3080	New Zealand		KP308760	JN974983	KJ811594	[51]
*I. leucophaea*	96095 (PC)	Zambia			EU569860		[47]
*I. lilacinosquamosa*	MCA 1464	Guyana			AY380386	AY337389	[46]
*I. lineata*	DED 8048	Thailand			GQ892958	KM245999	[54]
***I. lutosa***	**FYG2015298**	**China**	**fagaceous trees**	**PP993814**	**PP993851**		**This study**
***I. lutosa***	**FYG2015290**	**China**	**fagaceous trees**	**PP993815**			**This study**
***I. lutosa***	**FYG2015298a**	**China**	**fagaceous trees**	**PP993801**	**PP993843**	**PQ001014**	**This study**
***I. lutosa***	**FYG2015298b**	**China**	**fagaceous trees**	**PP993802**	**PP993844**		**This study**
***I. lutosa***	**FYG2015298c**	**China**	**fagaceous trees**	**PP993803**	**PP993845**	**PQ001015**	**This study**
***I. luxiensis***	**FYG2857**	**China**	***Fagaceae* and *Pinaceae* forests**	**PP217733**	**PP230771**	**PP238450**	**This study**
***I. luxiensis***	**NJ 3961**	**China**	***Fagaceae* forests**	**PP217743**	**PP230772**	**PP238462**	**This study**
***I. luxiensis***	**20230815-9**	**China**	**broad-leaved forests**	**PP993832**	**PP993857**	**PQ001025**	**This study**
*I. melanopus*	PBM 3975	Tennessee			MH220276	MH249807	[82]
*I. mixtilis*	ARAN-Fungi 4711	Spain		MH500842	MH500842	MH496022	[83]
*I. multicoronata*	DG 1818 A	Canada		MH578007	MH539763		Direct sub.
*I muthangensis*	K(M) 191735	India		NR160440	NG_064381	KY553247	[84]
*I. napipes*	PBM 2376	Norway			AY239024	AY337390	[85]
*I. nitidiuscula*	G 49	Estonia		AJ534934	AJ534934		[86]
*I. nothomixtilis*	AH 46558, MC 0003	Spain, Italy		MT384015		MH496025	[83]
*I. oblectabilis*	BJ 920908	Sweden		AM882831	AM882831		[74]
*I. obtusiuscula*	PAM 02081710	France		HQ586869	HQ641112		Direct sub.
*I. occulta*	AH 36443	Spain		NR_160564		MH496017	[83]
*I. olivaceonigra*	T. Bau & Y.G. Fan 2011131	China	*Fagaceae* forests	JX025775			[53]
*I. olivaceonigra*	T. Bau 2011131d	Chin	*Fagaceae* forests	JX025776			[53]
***I. olivaceonigra***	**FYG2015350**	**China**	***Fagaceae* forests**	**PP217746**	**PP230777**	**PP238454**	**This study**
***I. olivaceonigra***	**FYG2015351**	**China**	***Fagaceae* forests**	**PP217736**	**PP230776**	**PP238453**	**This study**
*I. pallidiangulata*	MR 00377	Burkina Faso		MN096202	MN097894	MW219732	[59]
*I. pallidiangulata*	MR 00379	Burkina Faso		MZ605434			[59]
*I. pallidicremea*	PBM 2448	USA		HQ201357	HQ201357	MF416425	[1]
*I. paludinella*	4873	Russia		MH930190			Direct sub.
*I. paludinella*	1991210-011			HQ604370			Direct sub.
*I. paludinella*	9774	Italy		JF908135			[69]
*I. paludinella* f. *citrophylla*	HRL 0428	Canada		KX897418			Direct sub.
***I. paludinelloides***	**YGF2011143**	**China**	***Castanopsis* and *Pinus***	**MG938541**	**MG825002**		**This study**
***I. paludinelloides***	**FYG10551**	**China**	***Castanopsis* and *Pinus***	**PQ358434**			**This study**
***I. paludinelloides***	**Tang 3013**	**China**	**Fagaceous trees mixed with *Ericaceae* or *Pinus***	**PP217741**	**PP230794**	**PP238467**	**This study**
***I. paludinelloides***	**Huang 559**	**China**	**fagaceous trees mixed with *Ericaceae* or *Pinus***	**PP217763**	**PP230795**	**PP238465**	**This study**
*I. papilliformis*	CAL 1372	India		KY440096	KY549126		[71]
*I. parvibulbosa*	DED 8021	Thailand		GQ892999	GQ892954	KM555134	[54]
*I. peppa*	NJ 4118	China	fagaceous trees	OR975591	OR975610	PP356984	[60]
*I. peppa*	NJ 4117	China	fagaceous trees	OR975592	OR975611	PP356980	[60]
*I. perlucida*	DB 20-8-16-33	Germany		MN803157	MN803157		[87]
*I. perlucida*	PBM 4328	USA		MT228849	MT228849		Direct sub.
*I. persicinipes*	PBM 2197	Australia		KF977215	EU600837	EU600836	[47,51]
*I. petiginosa*	2050510-012			HQ604373	HQ604373		Direct sub.
*I. petiginosa*	EL 6304	Sweden		AM882708		AM882708	[72]
*I. pileosulcata*	CAL 1362	India		KY440098	KY549128		[71]
*I. pingala*	CAL 1345	India		KY440100	KY549130		[71]
*I. pluppiana*	SMNS-STU-F-0901254	Netherlands		MN512327	MN512327		[87]
*I. praetervisa*	UBC: F19334	Canada		HQ604401	HQ604401		Direct sub.
*I. praetervisa*	SF 229598	Italy		KT203792			[88]
*I. pseudoasterospora*	STU:SMNS-STU-F-0901288	Italy		MN803152	MN803152		[87]
*I. pseudoasterospora*	a			MZ615409	MZ615409		Direct sub.
*I. pulchella*	MCA 1122	Guyana			EU600842		[47]
*I. relicina*	JV 10258	Finland		AY038324	AY038324	AY333778	[73]
*I. sapinea*	BJ 910825	Sweden		AM882797			[72]
*I. serrata*	PBM 3235	Australia		KP636810	KP171012	KM555111	[51]
***I. simaoensis***	**FYG2015395**	**China**	***Fagaceae* forests**	**PP217734**			**This study**
***I. simaoensis***	**FYG2858**	**China**		**PP217744**			**This study**
*I. snigdha*	CAL 1350	India		KY440105	KY549135	KY553250	[71]
*I. soluta*	EL 2904	Sweden		AM882755		AM882755	[72]
*I.* sp.	YM 3065	Japan	*Carpinus* sp.	AB848498			[89]
*I.* sp.	ZJ 0002SGS01			KU836546			Direct sub.
*I.* sp.	iNAT: 131262315	USA		OQ023949			Direct sub.
*I.* sp.	iNAT: 131258791	USA		OQ023943			Direct sub.
*I.* sp.	iNat: 131262115	USA		OQ389434			Direct sub.
*I.* sp.	21560			JF908227			[69]
*I.* sp.	MES-3836	USA		ON383409			Direct sub.
*I.* sp.	CWH-asv-0214		old *Picea sitchensis* and *Tsuga heterophylla*	OQ410765			Direct sub.
*I.* sp.	DED 8161	Thailand	under *Pinus kesiya* montane primary forests	GQ892985	GQ892939	MH577448	[54]
*I.* sp.	LM 5581	Hungary	*Quercus petraea*	KM576450			Direct sub.
*I.* sp.	iNat 135448009	Canada		OQ389439			Direct sub.
*I.* sp.	EMF 19	China		JF273522			Direct sub.
*I.* sp.	SFC20220920-B 534	South Korea	Sea sand	OP597927			[90]
*I.* sp.	DED 8147	Thailand	*Dipterocarpus* spp.		GQ892940		[54]
*I.* sp.	DED 8050	Thailand	near *Castanopsis* (*Fagaceae*)		EU569852		[47]
*I.* sp.	MR 00219	Australia		KF830031	KF808343	KF830049	[51]
*I.* sp.	TO-2011	Italy		JF908197	JF908197		[69]
*I.* sp.	FYG1146b	China	*Populus*	OR759138	OR760463	OR775215	[60]
*I.* sp.	130822 MFBPL 0312	China		MW554479	MW554479		Direct sub.
***I. spectabilis***	**FYG2342**	**China**	**Birch forest mixed with *Populus davidiana***	**PP217738**	**PP230779**	**PP238455**	**This study**
***I. spectabilis***	**FYG2015067**	**China**	***Betula*** **, *Pinus*, *Acer***	**PP217766**	**PP230798**	**PP238461**	**This study**
***I. spectabilis***	**FYG2015067a**	**China**	***Betula*** **, *Pinus*, *Acer***	**PP993804**			**This study**
*I. spiniformis*	PBM 3748	Australia		KP636868	KP171064	KM656103	[51]
*I. stellata*	ZT 10097	Thailand		GQ893008	GQ892963		[54]
*I. stellatospora*	EL 3004	Sweden		AM882747		AM882747	[72]
*I. suaveolens*	PK 4491			HQ604201	HQ604201		Direct sub.
*I. suaveolens*	PK 4812			HQ604204	HQ604204		Direct sub.
*I. suaveolens*	PK 4401			HQ604202	HQ604202		Direct sub.
*I. suaveolens*	PK 4996			HQ604205	HQ604205		Direct sub.
*I. suaveolens*	PK 4434			HQ604197	HQ604197		Direct sub.
*I. suaveolens*	PK 3788			HQ604196	HQ604196		Direct sub.
*I. suaveolens*	PK 3646			HQ604195	HQ604195		Direct sub.
*I. suaveolens*	WTU: Stz 4800	USA	under conifers	HQ222010			Direct sub.
*I. suaveolens*	PK 3890			HQ604200	HQ604200		Direct sub.
*I. subangustifolia*	NLB 983	Australia	mixed *Eucalyptus* forests	KP636871	KP171068	KM656113	[51]
*I. subangustifolia*	REH 9319	Australia	under *Eucalyptus*	KP636870	KP171067	KM656112	[51]
*I. subangustifolia*	BRI-794269	Australia		KP636870	NG_057268	KM656112	[51]
*I. subcarpta*	EL 8905	Finland		AM882754	AM882754	―	[74]
*I. subferruginea*	PERTH: 08074437	Australia		KP636874	KP171070	KM656115	[51]
*I. subfibrosoides*	MES-2512	Chile	*Nothofagus pumilio* forests	MT367480	MT367480	MT374786	[91]
*I. subfibrosoides*	MES-543	Chile	under *Nothofagus*	KP636879	KP171073	KM656117	[51]
*I. sylvicola*	TENN: 065735	Australia		NR_153163	NG_057199	KM656126	[51]
*I. thailandica*	DED 8049	Thailand		GQ893013	GQ892968	KM656129	[54]
*I. torresiae*	PBM 2157/E 6978	Australia			EU600874	EU600873	[54]
*I. torresiae*	PBM 3779	Australia		KP641635	KP171088	KM656132	[51]
*I. torresiae*	PBM 3722	Australia		KP641634	KP171087	KM656131	[51]
*I. tubarioides*	PBM 2550	USA		MK429956	AY732211	EU307855	[1]
*I. umbratica*	HMJAU 63754	China		OR364571			Direct sub.
*I. umbratica*	PBM 2552	USA			AY732209	EU307844	Direct sub.
*I. umbratica*	4806	Russia		MH930179			Direct sub.
*I. umbratica*	BJ 920804	Sweden		AM882799			[72]
*I. umbratica*	SJ 03020	Sweden		AM882798	AM882798		[72]
*I. umbratica*	AJ 118			GQ994981	―		Direct sub.
*I. umbratica*	1991204-024			HQ604477	HQ604477		Direct sub.
***I. umbratica***	**FYG3780**	**China**	***Populus*** **, *Betula*, *Picea*, *Abies*, and *Pinus***	**PP217737**			**This study**
***I. umbratica***	**FYG3753**	**China**	**coniferous forests**	**PP217747**			**This study**
***I. umbratica***	**FYG251**	**China**	**coniferous forests**	**PP217748**	**PP230778**		**This study**
***I. umbratica***	**FYG3995**	**China**	**coniferous forests**	**PP217764**	**PP230796**	**PP238460**	**This study**
***I. umbratica***	**FYG4034**	**China**	**coniferous forests**	**PP217765**	**PP230797**		**This study**
***I. umbratica***	**FYG8538**	**China**	**coniferous forests**	**PP993808**			**This study**
***I. umbratica***	**FYG2015086**	**China**	**coniferous forests**	**PP993807**			**This study**
***I. umbratica***	**FYG2015065**	**China**	**coniferous forests**	**PP993806**	**PP993847**		**This study**
***I. umbratica***	**FYG1880**	**China**	**coniferous forests**	**PP993821**			**This study**
***I. umbratica***	**CFSZ 24948**	**China**	***Picea* forests**	**PP993834**			**This study**
*I. viraktha*	CAL 1357	India		KY440107	KY549137		[71]
*I. viscata*	PBM 3445	Australia		KP641649	JQ313570		[51]
*I. viscata*	PBM 3213	Australia		KP641648	KP171097	MH618211	[51]
*I. viscata*	TENN-066121	Australia		KP641647	KP171096	MH577490	[51]
*I. xanthomelas*	PAM 08082901	France		HQ586856	HQ641097		Direct sub.
*I. xerophytica*	GUA 242	British Virgin Islands			EU600880		[47]
*I. parvifulva*	TENN-067015	Australia		NR152367			[51]
*Nothocybe distincta*	CAL 1310	India		KX171343	NG_057278	KX171345	[1]
*Nothocybe distincta*	ZT 9250	India			EU604546	EU600904	[47]
uncultured fungus	mOTU 20	China	*Pinus*	MN549484			Direct sub.
uncultured fungus	766 I 4	Portugal	*Quercus rotundifolia*	FJ897196	FJ897196		[92]
uncultured fungus	402 TKB mateba	Japan		LC806713			Direct sub.
uncultured fungus	T-b	South Korea		AB506085			[93]
uncultured fungus	P 09007	South Korea		AB587748			[94]
uncultured *Inocybe*	ECM 121	China		JQ991746			Direct sub.
uncultured *Inocybe*	Ea 234	Poland	*Epipogium aphyllum*	KX867492			[95]
uncultured *Inocybe*	SzYM 1125	Russia	*Larix gmelinii*	LC547570			[96]
uncultured *Inocybe*	sYM 1408	Russia	*Larix cajanderi*	LC574385			[97]
uncultured *Inocybe*	d1cII 001	Italy	*Quercus ruber*	HF565071			Direct sub.
uncultured *Inocybe*	szYM 1273	Russia	*Betula* sp.	LC547568			[96]
uncultured *Inocybe*	UVIC 48	Canada	*Pseudotsuga menziesii*	KT272127			[98]
uncultured *Inocybe*	Morph SrS			JQ791138			[99]
uncultured *Inocybe*	Ino 6	Thailand		AB854674	AB854674		[100]

Note: taxa and their sequences generated in this study are in bold.

## Data Availability

The original contributions presented in the study are included in the article, further inquiries can be directed to the corresponding author.
